# Supplementary material for: Altered microRNA Transcriptome in Cultured Human Liver Cells upon Infection with Ebola Virus
Source: Int J Mol Sci. 2021 Apr 6;22(7):3792. doi: 10.3390/ijms22073792 (PMC8038836; doi:10.3390/ijms22073792)
Supplement: Supplementary file 1 [file ijms-22-03792-s001.zip › Supplementary_File/C_ GO_Analysis_Results/16-30nt_go_Mayinga-24h-Huh7_vs_Control-24h-Huh7_up.mature_mirna_targets/CC_result(Human).html]

| GO.ID | Term | Ontology | Count | Pop.Hits | List.Total | Pop.Total | Fold.Enrichment | Pvalue | FDR | Enrichment.Score | Gene.Ratio | GENES |
| --- | --- | --- | --- | --- | --- | --- | --- | --- | --- | --- | --- | --- |
| GO:0044424 | intracellular part | Cellular component | 855 | 14395 | 970 | 18698 | 1.14492718333614 | 8.24014533583644e-20 | 7.0782848434835e-17 | 19.084065128352 | 0.881443298969072 | TOPORS//WWP2//FBXL5//ANKIB1//RNF2//IER5//PPP2CA//STRN//ZFC3H1//GNAQ//CDK6//CDKN1A//CCNC//CCND2//WFS1//RANBP9//IQCJ-SCHIP1//TOB1//ADAM10//CALCOCO2//IRF9//CORO2B//DLC1//LYPLA1//CEBPB//TACC2//CDC42EP3//TRIM3//EXOC5//PTGES3//MAP3K2//TOB2//IQGAP2//ZNF268//WASF3//ADCY3//BTG3//STMN2//PTPN21//FZD10//AKAP11//DUSP10//EGLN3//KLHL2//PCMTD1//FAM129A//ARAP2//FGD4//SLAIN1//FRMD6//NAA30//LYPD6//CPEB2//PTPDC1//CRK//HECTD2//SESN3//RBFOX3//CTNND2//PPP4R2//FGD5//RDH10//DGKH//GADD45A//DDX6//DCP2//LCA5//DNMT3B//DUSP1//DUSP2//DUSP5//E2F5//EDN1//EGR2//EPHA2//EPHA4//EPHA5//EPHA7//ERG//ESR1//ETS1//CCNY//NAPEPLD//KCTD20//ZNRF2//CPEB3//DZIP1//WDR47//MAPRE3//WDFY3//KLHDC10//PDZD2//LRCH1//OTUD3//FMR1//ZFPM2//CD2AP//FLRT1//CNOT6L//NR5A2//CNEP1R1//DSTYK//BAMBI//ZDHHC5//CLIC4//NSMF//APPL1//SACS//RANBP6//ARFIP1//SESN1//GLRB//GOLGA3//GOLGA4//NKIRAS1//MKNK2//GRB2//CNOT7//PSAT1//MDFIC//SCHIP1//NRBF2//ST6GALNAC6//NRG1//HMGB3//HPRT1//AGFG1//AGFG2//HSBP1//SPOPL//NANOS1//IDH1//IGF1//IMPDH1//IRF1//AR//JAK1//RBM44//PTAR1//KDR//IPO5//RHOC//RND3//ARHGAP1//ARHGAP6//MAF//MBNL1//DNAJB9//MEF2D//MEOX2//MET//ATXN3//MYO1B//MSN//PPP1R12A//NCK1//NEFH//NEUROD1//NF2//NFE2//ACO1//NME1//NPAT//NR4A2//MINK1//DCAF8//MEMO1//PAIP2//CDK17//ERGIC2//TAOK3//LGSN//PIAS4//ZFR//RAB23//CMPK1//ARID4B//PFKP//PFN2//PIK3CB//PKD2//PLEK//ERRFI1//SNTG1//ZFAND6//POU4F1//EGLN1//NDFIP2//PPP1R12C//ASPN//MTMR10//PXK//RALGPS2//ARMC1//UBE2W//FBXW7//PRKAA1//CDC37L1//RUFY2//PRKAR2B//CMTR2//LMO3//MAPK1//SLC2A4RG//PAK6//PTEN//PTHLH//RIMKLB//HECW2//TWF1//MIER1//PTPN4//RAP2C//RBM25//RRAGD//RARB//PLEKHA1//RGS3//RNF4//ATXN1//MOAP1//MAP2K4//CPEB1//AIDA//SKP1//UBE2Z//BMPR2//CAPRIN2//MTMR9//SNAP25//BNIP3//SNX2//SOS1//SOX4//SOX11//SPOCK1//STAT3//HSPA13//BTG1//ACTC1//TGFB1I1//TSC1//TSG101//UBE2I//UGP2//VBP1//PTP4A1//BTG2//AHNAK//ZNF655//BAG6//NPEPL1//VASH2//UBA5//DOCK5//UXS1//CPEB4//CALM2//DDHD1//FXR1//SNN//BTBD10//NSMAF//PHYHIPL//PPP1R15B//MASTL//PPFIA1//USP45//DGKE//UNK//BHLHE40//CDC14A//RUNX3//URI1//PER2//SQSTM1//FUBP3//RPS6KA4//UBE3B//CCNG2//C5ORF30//CCT6A//ZNF804A//RPS6KA5//KLF4//TRIP10//HAUS8//SLIT2//ZFAND4//RAB28//PLAA//HAND1//QKI//FEZ2//FNIP1//SOCS5//SLK//GIT2//MFAP3L//G3BP2//FOXF1//FOXF2//GATA6//JUN//NHLH2//NPAS2//PBX3//POU3F2//TRERF1//RBL2//SOX17//ZEB1//TEAD1//NR4A3//TCF7L1//HAND2//CLOCK//PRKACB//PPP3R1//BAG5//TRAPPC8//CNOT6//SNX5//HMGA2//CCNE2//EPS15//KLHL14//PRDM16//EDEM1//NEFM//LBR//MICU3//SLC25A32//COX7A2L//BCL2L11//ACSL1//SLC44A1//GJA1//MTX3//PMAIP1//MIEF1//JARID2//DPY30//KMT2B//REV3L//GDF11//OSBPL5//KLF6//VPS37A//DENND1B//S1PR1//CC2D1B//STX2//MYCBP2//CIC//RAB30//GLRA1//GRHL1//FOXN2//MIA3//NOVA1//ACOX1//UGT1A10//UGT1A8//UGT1A7//UGT1A6//UGT1A5//UGT1A9//UGT1A4//UGT1A1//UGT1A3//PPARG//CTSA//C7ORF43//SYBU//SPHK2//PMEPA1//RBBP8//SHOX//SLC2A4//SLC14A1//TFAP2A//LDLRAD4//EVI5//FYCO1//CLIP4//CALD1//AP1S2//BTAF1//ZFYVE9//PPT2//ITM2B//PLEKHF2//ULK2//ATG16L1//DEK//ACOT7//FAM210A//CCDC58//DYNLL2//E2F1//ELK3//PIKFYVE//PPM1E//RAB38//GLRX//GLUD2//SPRYD4//ZADH2//TCAIM//COX8C//BOLA3//CHCHD10//MMP2//P2RY1//DNAJC27//PON2//DUS2//PPP6C//BCAT2//ACBD3//TFAP2C//YWHAG//ZNF217//DCTPP1//APOO//DNAJC30//FAM110B//HIPK3//FAM13A//STX6//SEC23A//POLR3G//DCTN6//GNB5//PDE10A//COPS8//CKAP4//RAPGEF4//DNAJB4//KLF12//FCHO2//TADA1//TAGAP//CLCN5//NEDD1//CNN3//OSR1//MTPN//UBXN2B//CRKL//CRY2//SYT6//SH3D19//DENND5B//SPRED1//DCX//DYNC1LI2//DPYSL2//RAB12//RASGEF1A//RAB3GAP1//FOXC1//RAB21//GOLGA8A//PALLD//CDK19//FLI1//FLII//EFR3A//RGL1//UBXN4//DNAJC16//DOCK9//LCLAT1//GAB1//PAN3//DNAJB5//TMEM87A//NECAP1//TES//STEAP2//KLHL3//LATS2//GJB2//SERP1//RASSF3//FAM126B//OSTM1//MYLIP//GSTA2//GTF2E1//EPN1//MNX1//KRT27//NUP43//ITGAV//KIF5A//USP27X//MAN1A1//MAP3K5//NR3C2//MPP1//MZT1//MTRR//NSF//ARHGEF4//SAR1B//RLIM//RAB9B//YTHDF2//LIMA1//PEX5L//CHMP3//BRWD1//CC2D1A//CENPQ//ELMOD1//CDCA7L//NAGK//NPLOC4//MAP7D1//ENAH//DCAF6//MDM1//FEM1C//SLAIN2//RANBP10//ZBTB4//MID1IP1//RAD21//RAP1B//ARID4A//RBBP7//REST//RHEB//RPL15//RRM2//CLIP1//SRSF2//ARHGEF28//AKTIP//NABP1//SOS2//STAT4//BTF3//TGFBR2//THOP1//THRA//TMF1//TMOD1//ZFYVE21//CENPO//RNF128//CEP97//PPCS//TBC1D17//SNIP1//CCDC6//FOSL1//SBF2//FAM160A2//FBXO30//KBTBD8//GLYR1//NUDCD1//PIK3R3//RHPN2//CDC23//WASL//LMLN//LONRF1//CAMK2N2//ARHGAP12//KIF23//CEP41//PPM1F//USP6NL//JOSD1//GFPT2//RTN3//CDS1//CRTAP//COL1A2//TBC1D20//VKORC1L1//TMEM64//SMIM14//SULF1//ZDHHC1//ITGA5//FIBIN//SHISA2//LAMA3//LRP6//FAM19A1//ASPH//DERL2//IER3IP1//TMX3//TMED9//DNAJB12//TMEM100//RGMA//PHTF2//STIM2//RTN1//MSMO1//ELOVL4//ELOVL7//CAMLG//B3GNT5//B3GALT2//ORMDL3//RGMB//STK17B//FUT9//SCRG1//ESCO2//MMD//LDLR//C11ORF24//FAM20C//PLEKHA3//GNPTG//SGCE//CCDC126//PKDCC//MMGT1//CXCL14//LAPTM4A//DSC2//RGS17//DLL1//NTF3//CHIC1//ANP32E//CNIH1//GRIA1//MCFD2//USP3//SERINC3//INHBB//RASD1//STK33//CALN1//FASTK//PABPC5//ANO8//WNT7A//KDELC1//OGN//HS3ST1//SERPINI1//GNS//KAT2B//DR1//KLHL15//TMOD3//NPNT//ARRDC3//TBC1D12//FRY//EPS8//MARCKS//SNAPC1//E2F3//C6ORF120//TTR//TMEM135//ACBD5//TSHZ1//CDK8//HMG20A//ZBTB18//KHDRBS3//ZMYND11//TCERG1//ZNF597//RNF38//CREBRF//ZNF782//NKX2-3//ZNF800//GLIS3//ARX//DMWD//DRD1//ZNF367//EMX2//EN1//ZBTB9//FHL2//FOXJ3//MYT1L//LHX6//ATAD2//PURG//SERTAD3//H2AFZ//HIVEP2//ZBTB41//ZNF568//LHX1//MDM4//MLLT6//MYB//MYBL1//NAP1L3//NEUROG1//NFIA//NFIB//OTX2//SYCP3//PAX9//ZBTB7A//RBM47//CCNJ//RSBN1//ESRP1//SOBP//ZNF532//BTBD7//ZNFX1//ZNF630//ZNF287//ZNF512B//TSHZ3//ZBTB26//DMRTA1//NKX2-4//GMNC//RFX7//BCL11B//TCF21//TEF//KLF10//ZNF2//ZNF3//ZNF131//BHLHE41//TBL1XR1//BRD3//SGPP1//ARID5B//KIAA1109//PCGF5//MSANTD4//ZIC5//KLF7//RTCA//ZBTB47//PHF14//ST18//MAN2A1//PSD//LPAR1//ANKRD50//ARRDC4//ADRB1//RAB22A//TNF//PGRMC2//NUP35//RNF180//NLN//MARCH4//FAM91A1//C16ORF70//EZH1//TDRD5//FBXO28//MTBP//YPEL2//ZMAT3//CETN1//CEP128//CFL2//TMEM63B//SESTD1//BAHD1//KLF2//SMARCD2//BRMS1L//PDIK1L//ELAVL2//KDM2A//USP24//MORC3//ST6GALNAC3//HBP1//HOXA3//HOXD1//ZBTB34//KLF3//CTDSPL2//BNC2//INTS8//BDP1//REXO1//USP28//SCN1A//TGIF1//ADAM12//LCOR//CBFB//WDR20//COX7A2//KIF13A//REEP3//LRP8//HS3ST5//ST8SIA5//ST8SIA3//CHST7//XYLT2//ST3GAL3//ST6GAL2//CHST1//ST3GAL5//DSEL//HS2ST1//HS3ST3A1//CASD1//PIGA//ARL6IP1//GJC1//PPM1L//SPTSSA//SLC35D1//JKAMP//SCD5//GSG1//CLCN6//LAMTOR3//SLC32A1//SLC17A7//SLC17A6//GPRC5C//FZD6//NHLRC2//TGFB2//VEGFB//EPC2//LHX8//MCOLN2//GPR137B//VLDLR//CYBRD1//LHFPL2//ENPP4//PLEKHB2//FAM118B//CHRM2//IMMP2L// |
| GO:0005622 | intracellular | Cellular component | 863 | 14673 | 970 | 18698 | 1.13374477703279 | 1.38018835027968e-18 | 5.92790896445123e-16 | 17.8600616426528 | 0.889690721649484 | TRERF1//ZNF217//TBL1XR1//TADA1//KAT2B//BDP1//GOLGA8A//CNIH1//ADAM10//STX6//RTN3//SEC23A//FUT9//SERINC3//CLCN5//PIKFYVE//RAB12//HS3ST5//ST6GALNAC3//GJA1//ARFIP1//RAB30//GOLGA3//GOLGA4//GRIA1//ST8SIA5//ST6GALNAC6//MIA3//RND3//MAN1A1//MAN2A1//NSF//ST8SIA3//NDFIP2//TMED9//PPP6C//SYBU//CHST7//PMEPA1//MARCH4//RHEB//XYLT2//ACBD3//ST3GAL3//PLEKHA3//TMF1//APOO//B3GNT5//GNPTG//ST6GAL2//CHST1//B3GALT2//ST3GAL5//AP1S2//MCFD2//DSEL//MMGT1//ITM2B//HS2ST1//EDEM1//HS3ST3A1//EXOC5//TOPORS//WWP2//FBXL5//ANKIB1//RNF2//IER5//PPP2CA//STRN//NCK1//PPP1R15B//ZFC3H1//RPL15//ZBTB18//JUN//HMGA2//NEDD1//GNAQ//CDK6//CDKN1A//CCNC//CCND2//CCNY//TRAPPC8//PLEKHF2//SQSTM1//ULK2//CALCOCO2//ATG16L1//WFS1//PIGA//FMR1//SYCP3//CENPQ//RAD21//DYNC1LI2//FBXO28//MTBP//NUP43//PPP1R12A//CLIP1//DCTN6//CENPO//CEBPB//PTGES3//DPY30//NABP1//ESCO2//BAHD1//MAF//RBL2//H2AFZ//GLYR1//KLF2//POLR3G//E2F1//ESR1//IRF1//AR//MEF2D//POU4F1//SMARCD2//STAT3//RUNX3//KLF4//HAND2//USP3//DDX6//UBE2I//PCGF5//ANP32E//VPS37A//TSG101//CHMP3//FRY//CETN1//CEP128//LATS2//CALM2//CDC14A//HAUS8//MZT1//DCP2//PAN3//CNOT7//YTHDF2//CPEB1//KLF6//E2F5//SESN1//NFIB//CC2D1A//CDCA7L//PAK6//BTBD10//LHX8//BCL2L11//DYNLL2//KRT27//NME1//LIMA1//PALLD//DEK//RAB38//LDLR//PON2//CTSA//ARRDC3//RRAGD//SNX2//FYCO1//TRIP10//PPT2//LYPLA1//ACOT7//FAM210A//CCDC58//ELK3//ACSL1//PPM1E//SLC44A1//CLIC4//SACS//GLRX//GLUD2//SPRYD4//ZADH2//TCAIM//IDH1//COX8C//JARID2//BOLA3//CHCHD10//MMP2//NEFH//ACO1//P2RY1//DCAF8//ACOX1//DNAJC27//PMAIP1//MIEF1//DUS2//ARMC1//PPP3R1//MAPK1//PTEN//BCAT2//MOAP1//CAPRIN2//BNIP3//SOX4//TFAP2C//YWHAG//DCTPP1//UXS1//SLC25A32//DNAJC30//PHYHIPL//URI1//FAM110B//COX7A2L//BAG5//RANBP9//HIPK3//FAM13A//IRF9//DLC1//TACC2//CDC42EP3//GNB5//MAP3K2//TOB2//IQGAP2//ZNF268//PDE10A//COPS8//CKAP4//RAPGEF4//DNAJB4//AKAP11//DUSP10//EGLN3//KLHL2//KLF12//OSBPL5//FCHO2//FAM129A//ARAP2//TAGAP//FGD4//NAA30//CNN3//OSR1//MTPN//UBXN2B//CRK//CRKL//CRY2//HECTD2//SYT6//SH3D19//DENND5B//SPRED1//DENND1B//DCX//DPYSL2//EPS15//RASGEF1A//NAPEPLD//ZNRF2//DZIP1//RAB3GAP1//FOXC1//WDFY3//RAB21//CDK19//FLI1//FLII//EFR3A//RGL1//UBXN4//OTUD3//DNAJC16//DOCK9//CNOT6L//LCLAT1//GAB1//CNEP1R1//DSTYK//DNAJB5//TMEM87A//NECAP1//APPL1//TES//STEAP2//KLHL3//GJB2//SNX5//SERP1//RASSF3//FAM126B//GRB2//OSTM1//MYLIP//GSTA2//GTF2E1//GRHL1//EPN1//PSAT1//MNX1//HPRT1//AGFG1//AGFG2//HSBP1//IMPDH1//ITGAV//JAK1//KIF5A//RHOC//USP27X//ARHGAP1//ARHGAP6//MBNL1//MAP3K5//ATXN3//NR3C2//MPP1//MSN//MTRR//NF2//NPAS2//MINK1//ARHGEF4//MEMO1//SAR1B//RLIM//RAB9B//PEX5L//RAB23//CMPK1//PFKP//PIK3CB//PLEK//BRWD1//ERRFI1//EGLN1//PPARG//MTMR10//PXK//FBXW7//ELMOD1//NAGK//PRKAA1//CDC37L1//NPLOC4//PRKACB//MAP7D1//ENAH//PRKAR2B//DCAF6//SPHK2//MDM1//FEM1C//PTHLH//CNOT6//RIMKLB//TWF1//KLHL14//SLAIN2//RANBP10//ZBTB4//RAP2C//MID1IP1//RAP1B//ARID4A//RBBP7//PLEKHA1//REST//RGS3//RRM2//ATXN1//PRDM16//MAP2K4//SRSF2//ARHGEF28//AKTIP//SKP1//SLC2A4//UBE2Z//MTMR9//SNAP25//SOS1//SOS2//STAT4//HSPA13//BTF3//ZEB1//ACTC1//TFAP2A//TGFBR2//THOP1//THRA//TMOD1//TSC1//UGP2//VBP1//EVI5//BTG2//AHNAK//ZFYVE21//BAG6//RNF128//CEP97//PPCS//TBC1D17//SNIP1//UBA5//CALD1//DOCK5//CCDC6//FOSL1//DDHD1//FXR1//SBF2//TCF7L1//FAM160A2//FBXO30//NSMAF//KBTBD8//NUDCD1//PPFIA1//PIK3R3//DGKE//RHPN2//CDC23//WASL//LMLN//RPS6KA4//CCT6A//CCNE2//LONRF1//ZFYVE9//CAMK2N2//ARHGAP12//KIF23//CEP41//CLOCK//PPM1F//SOCS5//USP6NL//GIT2//G3BP2//JOSD1//GFPT2//TMEM135//ACBD5//TOB1//TSHZ1//CDK8//HMG20A//KHDRBS3//ZMYND11//TCERG1//BTG3//CPEB2//PTPDC1//SESN3//ZNF597//RBFOX3//CTNND2//PPP4R2//RNF38//CREBRF//ZNF782//NKX2-3//GADD45A//ZNF800//GLIS3//ARX//DMWD//DNMT3B//DR1//DRD1//DUSP1//DUSP2//DUSP5//ZNF367//EGR2//CC2D1B//EMX2//EN1//ERG//ETS1//ZBTB9//FHL2//CPEB3//FOXJ3//FOXF1//FOXF2//PDZD2//MYT1L//MYCBP2//CIC//ZFPM2//NR5A2//NSMF//GATA6//LHX6//RGS17//MKNK2//ATAD2//PURG//SERTAD3//MDFIC//HIVEP2//HMGB3//FOXN2//SPOPL//ZBTB41//ZNF568//KDR//IPO5//LHX1//MDM4//MEOX2//MLLT6//MYB//MYBL1//NAP1L3//NEUROD1//NEUROG1//NFIA//NFE2//NHLH2//NOVA1//NPAT//NR4A2//OTX2//PAX9//PBX3//CDK17//ERGIC2//ZBTB7A//TAOK3//PIAS4//RASD1//ZFR//ARID4B//SNTG1//RBM47//POU3F2//CCNJ//RSBN1//ESRP1//SOBP//ZNF532//UBE2W//RUFY2//BTBD7//CMTR2//SLC2A4RG//PHTF2//ZNFX1//ZNF630//ZNF287//ZNF512B//TSHZ3//ZBTB26//MIER1//RARB//RBBP8//REV3L//RNF4//DMRTA1//SOX17//NKX2-4//GMNC//RFX7//BCL11B//SNAPC1//SOX11//BTG1//TCF21//TEAD1//TEF//KLF10//ZNF2//ZNF3//ZNF131//PTP4A1//ZNF655//BHLHE41//NPEPL1//NR4A3//BRD3//KLHL15//CPEB4//SGPP1//ARID5B//KIAA1109//MSANTD4//MASTL//USP45//ZIC5//BHLHE40//KLF7//RTCA//PER2//FUBP3//ZNF804A//RPS6KA5//STK17B//ZBTB47//ZFAND4//PLAA//HAND1//QKI//PHF14//ST18//KMT2B//MFAP3L//PGRMC2//NUP35//RNF180//LBR//IQCJ-SCHIP1//CORO2B//TRIM3//WASF3//ADCY3//STMN2//PTPN21//FZD10//PCMTD1//SLAIN1//FRMD6//LYPD6//FGD5//RDH10//DGKH//LCA5//EDN1//EPHA2//EPHA4//EPHA5//EPHA7//KCTD20//WDR47//MAPRE3//KLHDC10//LRCH1//CD2AP//FLRT1//BAMBI//ZDHHC5//RANBP6//GLRB//NKIRAS1//SCHIP1//NRBF2//NRG1//NANOS1//IGF1//RBM44//PTAR1//DNAJB9//MET//MYO1B//PAIP2//LGSN//PFN2//PKD2//ZFAND6//PPP1R12C//ASPN//RALGPS2//LMO3//HECW2//PTPN4//RBM25//AIDA//BMPR2//SPOCK1//TGFB1I1//VASH2//SNN//UNK//UBE3B//CCNG2//C5ORF30//SLIT2//RAB28//FEZ2//FNIP1//SLK//GDF11//PDIK1L//E2F3//S1PR1//ELAVL2//EZH1//KDM2A//USP24//MORC3//HBP1//HOXA3//HOXD1//ZBTB34//KLF3//CTDSPL2//BNC2//INTS8//REXO1//USP28//SCN1A//ZMAT3//SHOX//TGIF1//LDLRAD4//ADAM12//LCOR//CBFB//BTAF1//WDR20//YPEL2//SLC14A1//MTX3//MICU3//COX7A2//NLN//FASTK//PABPC5//CLCN6//MMD//MCOLN2//GPR137B//VLDLR//CYBRD1//LAMTOR3//LAPTM4A//LPAR1//ANKRD50//ARRDC4//ADRB1//LRP6//DERL2//RAB22A//TBC1D12//CDS1//CRTAP//COL1A2//TBC1D20//VKORC1L1//TMEM64//SMIM14//SULF1//GLRA1//ZDHHC1//ITGA5//FIBIN//SHISA2//LAMA3//FAM19A1//ASPH//IER3IP1//TMX3//UGT1A10//UGT1A8//UGT1A7//UGT1A6//UGT1A9//UGT1A4//UGT1A1//UGT1A3//DNAJB12//TMEM100//RGMA//STIM2//RTN1//MSMO1//ELOVL4//ELOVL7//CAMLG//ORMDL3//ARL6IP1//FAM20C//ANO8//WNT7A//KDELC1//GJC1//PPM1L//SPTSSA//REEP3//SLC35D1//JKAMP//UGT1A5//SCD5//GSG1//STX2//C7ORF43//CLIP4//RGMB//SCRG1//C11ORF24//SGCE//CCDC126//PKDCC//CXCL14//OGN//HS3ST1//FAM91A1//C16ORF70//MARCKS//KIF13A//TMOD3//LRP8//NEFM//EPS8//NTF3//FAM118B//CFL2//TMEM63B//CASD1//SLC32A1//SLC17A7//SLC17A6//NPNT//GPRC5C//FZD6//CHRM2//LHFPL2//NHLRC2//TGFB2//VEGFB//DSC2//DLL1//CHIC1//CALN1//EPC2//TDRD5//SERPINI1//GNS//C6ORF120//TTR//IMMP2L//SESTD1//INHBB//STK33//TNF//PLEKHB2//BRMS1L//PSD//ENPP4//EREG//ARL5A//IL2//TREM1//ARL15//PTGER4//CCL2//LONRF3// |
| GO:0043229 | intracellular organelle | Cellular component | 765 | 12604 | 970 | 18698 | 1.16997467666949 | 3.64547102329207e-16 | 1.0438198696693e-13 | 15.4382463494858 | 0.788659793814433 | GDF11//CALCOCO2//OSBPL5//ARAP2//KLF6//VPS37A//DENND1B//DNMT3B//S1PR1//EGR2//CC2D1B//STX2//EPS15//MYCBP2//CIC//APPL1//GJA1//RAB30//GLRA1//GOLGA4//GRHL1//AGFG1//AGFG2//FOXN2//MIA3//RHOC//RND3//MEF2D//NOVA1//ACOX1//ERGIC2//UGT1A10//UGT1A8//UGT1A7//UGT1A6//UGT1A5//UGT1A9//NDFIP2//UGT1A4//UGT1A1//UGT1A3//PPARG//CTSA//C7ORF43//SYBU//SPHK2//PMEPA1//RBBP8//SHOX//SLC2A4//SLC14A1//HSPA13//TFAP2A//VBP1//LDLRAD4//EVI5//BAG6//FYCO1//CLIP4//UBA5//CALD1//RUNX3//SQSTM1//AP1S2//BTAF1//TRIP10//ZFYVE9//PPT2//ITM2B//CLOCK//GIT2//BCL2L11//LYPLA1//ACOT7//FAM210A//CCDC58//DYNLL2//DDX6//E2F1//ELK3//PIKFYVE//ACSL1//PPM1E//SLC44A1//RAB38//CLIC4//SACS//GLRX//GLUD2//SPRYD4//ZADH2//TCAIM//IDH1//COX8C//JARID2//BOLA3//CHCHD10//MMP2//NEFH//ACO1//P2RY1//DCAF8//DNAJC27//PMAIP1//PON2//MIEF1//DUS2//PPP2CA//ARMC1//PPP3R1//PPP6C//MAPK1//PTEN//BCAT2//MOAP1//ACBD3//CAPRIN2//BNIP3//SOX4//TFAP2C//YWHAG//ZNF217//DCTPP1//APOO//UXS1//SLC25A32//DNAJC30//PHYHIPL//URI1//FAM110B//COX7A2L//BAG5//RANBP9//TOB1//TSHZ1//ADAM10//CDK6//TOPORS//CDK8//CDKN1A//HMG20A//IRF9//DLC1//ZBTB18//CEBPB//POLR3G//KHDRBS3//GNB5//PTGES3//TOB2//ZMYND11//ZNF268//TCERG1//COPS8//BTG3//WWP2//DUSP10//EGLN3//TADA1//NAA30//OSR1//CPEB2//MTPN//UBXN2B//PTPDC1//CRK//CRY2//SESN3//ZNF597//RBFOX3//CTNND2//PPP4R2//RNF38//CREBRF//ESCO2//ZNF782//NKX2-3//SPRED1//GADD45A//ZNF800//GLIS3//ARX//DMWD//DR1//DRD1//DUSP1//DUSP2//DUSP5//E2F5//ZNF367//ZFC3H1//EMX2//EN1//ERG//ESR1//ETS1//CCNY//ZBTB9//FHL2//CPEB3//DZIP1//FOXJ3//FOXF1//FOXF2//FOXC1//WDFY3//PALLD//PDZD2//MYT1L//CDK19//FLI1//FMR1//ZFPM2//CNOT6L//NR5A2//DNAJB5//NSMF//TES//GATA6//LHX6//LATS2//RGS17//SESN1//GOLGA3//MKNK2//GRB2//ATAD2//GTF2E1//CNOT7//EPN1//PURG//SERTAD3//MDFIC//H2AFZ//HIVEP2//MNX1//HMGB3//HSBP1//SPOPL//ZBTB41//IMPDH1//IRF1//AR//JAK1//JUN//ZNF568//KDR//IPO5//USP27X//LHX1//MAF//MBNL1//MDM4//MEOX2//ATXN3//MLLT6//MSN//MYB//MYBL1//NAP1L3//NCK1//NEUROD1//NEUROG1//NF2//NFIA//NFE2//NFIB//NHLH2//NME1//NPAS2//NPAT//NR4A2//OTX2//SYCP3//PAX9//PBX3//MEMO1//RLIM//IER5//CDK17//ZBTB7A//TAOK3//YTHDF2//PIAS4//RASD1//ZFR//CMPK1//ARID4B//PFKP//PIK3CB//BRWD1//ERRFI1//SNTG1//RBM47//POU3F2//POU4F1//EGLN1//CCNJ//RSBN1//ESRP1//CC2D1A//SOBP//ZNF532//UBE2W//CDCA7L//PRKAA1//NPLOC4//RUFY2//BTBD7//CMTR2//TRERF1//DCAF6//SLC2A4RG//MDM1//PAK6//PHTF2//ZNFX1//ZNF630//ZNF287//CNOT6//ZNF512B//RANBP10//TSHZ3//ZBTB4//ZBTB26//MIER1//MID1IP1//RRAGD//RARB//ARID4A//RBBP7//PLEKHA1//RBL2//REST//REV3L//RNF2//RNF4//RPL15//ATXN1//DMRTA1//PRDM16//MAP2K4//SRSF2//SOX17//NKX2-4//CPEB1//GMNC//NABP1//RFX7//BCL11B//SKP1//UBE2Z//SNAPC1//SOX11//STAT3//BTF3//ZEB1//BTG1//TCF21//TEAD1//TEF//THRA//KLF10//TMF1//TSC1//UBE2I//UGP2//ZNF2//ZNF3//ZNF131//PTP4A1//BTG2//AHNAK//ZNF655//DEK//BHLHE41//NPEPL1//TBL1XR1//SNIP1//NR4A3//BRD3//KLHL15//CPEB4//CALM2//FOSL1//FXR1//HMGA2//SGPP1//ANP32E//TCF7L1//ARID5B//KIAA1109//BTBD10//PCGF5//MSANTD4//DPY30//MASTL//USP45//DGKE//ZIC5//BHLHE40//CDC14A//KLF7//RTCA//KAT2B//PER2//CCNC//FUBP3//CCND2//WASL//RPS6KA4//ACBD5//ZNF804A//RPS6KA5//STK17B//ZBTB47//ZFAND4//PLAA//CAMK2N2//HAND1//QKI//KIF23//PHF14//ST18//KMT2B//MFAP3L//USP3//RTN3//CDS1//CRTAP//CKAP4//COL1A2//TBC1D20//FGD5//VKORC1L1//TMEM64//SMIM14//EPHA4//UBXN4//SULF1//LCLAT1//SERP1//ZDHHC1//ITGA5//FIBIN//SHISA2//LAMA3//LRP6//FAM19A1//MAN1A1//DNAJB9//ASPH//DERL2//IER3IP1//PKD2//TMX3//TMED9//DNAJB12//TMEM100//RGMA//KLHL14//STIM2//RTN1//MSMO1//ELOVL4//WFS1//RNF128//PLEKHF2//ELOVL7//CAMLG//B3GNT5//PPP1R15B//B3GALT2//ORMDL3//EDEM1//GJB2//RGMB//STX6//TRIM3//FUT9//ADCY3//STMN2//SCRG1//CLCN5//FGD4//RAB12//RAB3GAP1//GOLGA8A//MMD//LDLR//MINK1//C11ORF24//FAM20C//PTHLH//PLEKHA3//KBTBD8//GNPTG//GLYR1//SGCE//CCDC126//PKDCC//MMGT1//CXCL14//LAPTM4A//MAN2A1//DYNC1LI2//FBXO28//MTBP//NUP43//PPP1R12A//CLIP1//RAD21//AKAP11//YPEL2//FBXW7//ZMAT3//TSG101//RDH10//RAP1B//TMEM135//LMLN//CETN1//NEDD1//CEP128//CEP41//MZT1//MAP7D1//CDC42EP3//WASF3//PTPN21//SLAIN1//FRMD6//DCX//KLHL3//MYLIP//TMOD3//PFN2//ENAH//PTPN4//SNAP25//TGFB1I1//TMOD1//CCDC6//DSC2//TRAPPC8//FLRT1//DLL1//NRBF2//NTF3//CHMP3//CHIC1//USP6NL//DCTN6//CENPO//TWF1//NPNT//ARRDC3//SNX2//TBC1D12//RAB23//ATG16L1//TBC1D17//LHX8//EPHA5//HECW2//CORO2B//CFL2//IQGAP2//KLHL2//CNN3//CD2AP//ARHGAP6//MARCKS//LIMA1//CENPQ//TMEM63B//RAB22A//TACC2//SLAIN2//MPP1//NEFM//SESTD1//CRKL//DGKH//LPAR1//RAB21//STEAP2//SNX5//ANKRD50//ARRDC4//ADRB1//MYO1B//EDN1//RAB9B//FCHO2//ITGAV//GRIA1//TNF//IGF1//ARHGAP1//DCP2//PAN3//TDRD5//PEX5L//CLCN6//ZNRF2//MCOLN2//GNAQ//OSTM1//NSF//RHEB//GPR137B//VLDLR//CYBRD1//LAMTOR3//GNS//OGN//CNIH1//MCFD2//SEC23A//SAR1B//SERPINI1//WNT7A//FRY//HAUS8//SLC32A1//SYT6//SLC17A7//SLC17A6//C16ORF70//ACTC1//LCA5//FLII//PXK//CEP97//DPYSL2//REEP3//WDR47//RASSF3//KIF5A//KIF13A//CCT6A//LRP8//CHRM2//LHFPL2//NHLRC2//TGFB2//VEGFB//C6ORF120//TTR//CDC37L1//KRT27//PLEKHB2//RAP2C//ENPP4//BDP1//SERINC3//HS3ST5//ST6GALNAC3//ARFIP1//ST8SIA5//ST6GALNAC6//ST8SIA3//CHST7//MARCH4//XYLT2//ST3GAL3//ST6GAL2//CHST1//ST3GAL5//DSEL//HS2ST1//HS3ST3A1//PIGA//BAHD1//KLF2//SMARCD2//KLF4//HAND2//PGRMC2//NUP35//CNEP1R1//RNF180//LBR//MAP3K2//DNAJB4//FZD10//KLF12//PDIK1L//SH3D19//E2F3//ELAVL2//EZH1//KDM2A//KLHDC10//USP24//MORC3//HBP1//NRG1//HOXA3//HOXD1//ZBTB34//NR3C2//KLF3//CTDSPL2//BNC2//ELMOD1//INTS8//PRKACB//FEM1C//REXO1//USP28//RGS3//SCN1A//BMPR2//STAT4//TGIF1//ADAM12//LCOR//NUDCD1//CBFB//CDC23//CCNE2//WDR20//RBM25//MTX3//SNN//MICU3//PRKAR2B//COX7A2//NLN//THOP1//FASTK//PABPC5//SBF2//ARL6IP1//ANO8//KDELC1//GJC1//PPM1L//SPTSSA//SLC35D1//JKAMP//SCD5//GSG1//HS3ST1//FAM91A1//ZFYVE21//FAM118B//HIPK3//NECAP1//CASD1//GPRC5C//FZD6//ULK2//RANBP6//TMEM87A//CALN1//EPC2//RAB28//MAPRE3//IMMP2L//BRMS1L//PSD//MAP3K5// |
| GO:0043231 | intracellular membrane-bounded organelle | Cellular component | 676 | 10864 | 970 | 18698 | 1.19944506020072 | 1.41162796210106e-14 | 3.03147104861203e-12 | 13.8502797475451 | 0.696907216494845 | BCL2L11//LYPLA1//ACOT7//FAM210A//CCDC58//DYNLL2//DDX6//E2F1//ELK3//PIKFYVE//ACSL1//PPM1E//SLC44A1//RAB38//CLIC4//SACS//GJA1//GLRX//GLUD2//SPRYD4//ZADH2//TCAIM//IDH1//COX8C//JARID2//BOLA3//CHCHD10//MMP2//NEFH//ACO1//P2RY1//DCAF8//ACOX1//DNAJC27//PMAIP1//PON2//MIEF1//NDFIP2//DUS2//PPP2CA//ARMC1//PPP3R1//PPP6C//MAPK1//PTEN//BCAT2//MOAP1//ACBD3//CAPRIN2//BNIP3//SOX4//TFAP2C//YWHAG//ZNF217//DCTPP1//APOO//UXS1//SLC25A32//DNAJC30//PHYHIPL//URI1//FAM110B//COX7A2L//BAG5//RANBP9//TOB1//TSHZ1//ADAM10//CDK6//TOPORS//CDK8//CALCOCO2//CDKN1A//HMG20A//IRF9//DLC1//ZBTB18//CEBPB//POLR3G//KHDRBS3//GNB5//PTGES3//TOB2//ZMYND11//ZNF268//TCERG1//COPS8//BTG3//WWP2//DUSP10//EGLN3//TADA1//NAA30//OSR1//KLF6//CPEB2//MTPN//UBXN2B//PTPDC1//CRK//CRY2//SESN3//ZNF597//RBFOX3//CTNND2//PPP4R2//RNF38//CREBRF//ESCO2//ZNF782//NKX2-3//SPRED1//GADD45A//ZNF800//GLIS3//ARX//DMWD//DNMT3B//DR1//DRD1//DUSP1//DUSP2//DUSP5//E2F5//ZNF367//EGR2//ZFC3H1//CC2D1B//EMX2//EN1//ERG//ESR1//ETS1//CCNY//ZBTB9//FHL2//CPEB3//DZIP1//FOXJ3//FOXF1//FOXF2//FOXC1//WDFY3//PALLD//PDZD2//MYT1L//MYCBP2//CDK19//FLI1//CIC//FMR1//ZFPM2//CNOT6L//NR5A2//DNAJB5//NSMF//APPL1//TES//GATA6//LHX6//LATS2//RGS17//SESN1//GOLGA3//MKNK2//GRB2//ATAD2//GTF2E1//GRHL1//CNOT7//EPN1//PURG//SERTAD3//MDFIC//H2AFZ//HIVEP2//MNX1//HMGB3//AGFG2//HSBP1//FOXN2//SPOPL//ZBTB41//IMPDH1//IRF1//AR//JAK1//JUN//ZNF568//KDR//IPO5//RHOC//USP27X//LHX1//MAF//MBNL1//MDM4//MEF2D//MEOX2//ATXN3//MLLT6//MSN//MYB//MYBL1//NAP1L3//NCK1//NEUROD1//NEUROG1//NF2//NFIA//NFE2//NFIB//NHLH2//NME1//NOVA1//NPAS2//NPAT//NR4A2//OTX2//SYCP3//PAX9//PBX3//MEMO1//RLIM//IER5//CDK17//ERGIC2//ZBTB7A//TAOK3//YTHDF2//PIAS4//RASD1//ZFR//CMPK1//ARID4B//PFKP//PIK3CB//BRWD1//ERRFI1//SNTG1//RBM47//POU3F2//POU4F1//EGLN1//CCNJ//RSBN1//PPARG//ESRP1//CC2D1A//SOBP//ZNF532//UBE2W//CDCA7L//PRKAA1//NPLOC4//RUFY2//BTBD7//CMTR2//TRERF1//DCAF6//SLC2A4RG//MDM1//PAK6//PHTF2//ZNFX1//ZNF630//ZNF287//CNOT6//ZNF512B//RANBP10//TSHZ3//ZBTB4//ZBTB26//MIER1//MID1IP1//RRAGD//RARB//ARID4A//RBBP7//RBBP8//PLEKHA1//RBL2//REST//REV3L//RNF2//RNF4//RPL15//ATXN1//DMRTA1//PRDM16//MAP2K4//SRSF2//SOX17//NKX2-4//CPEB1//GMNC//NABP1//RFX7//BCL11B//SKP1//UBE2Z//SNAPC1//SOX11//STAT3//HSPA13//BTF3//ZEB1//BTG1//TCF21//TEAD1//TEF//TFAP2A//THRA//KLF10//TMF1//TSC1//UBE2I//UGP2//VBP1//ZNF2//ZNF3//ZNF131//PTP4A1//EVI5//BTG2//AHNAK//ZNF655//DEK//BAG6//BHLHE41//NPEPL1//TBL1XR1//SNIP1//UBA5//NR4A3//BRD3//KLHL15//CPEB4//CALM2//FOSL1//FXR1//HMGA2//SGPP1//ANP32E//TCF7L1//ARID5B//KIAA1109//BTBD10//PCGF5//MSANTD4//DPY30//MASTL//USP45//DGKE//ZIC5//BHLHE40//CDC14A//KLF7//RTCA//RUNX3//KAT2B//PER2//CCNC//FUBP3//CCND2//WASL//RPS6KA4//ACBD5//ZNF804A//RPS6KA5//STK17B//ZBTB47//ZFAND4//PLAA//CAMK2N2//HAND1//QKI//KIF23//CLOCK//PHF14//ST18//KMT2B//MFAP3L//USP3//RTN3//CDS1//CRTAP//CKAP4//COL1A2//TBC1D20//FGD5//VKORC1L1//TMEM64//SMIM14//EPHA4//UBXN4//SULF1//LCLAT1//SERP1//GLRA1//ZDHHC1//ITGA5//FIBIN//SHISA2//LAMA3//LRP6//FAM19A1//MAN1A1//DNAJB9//ASPH//DERL2//IER3IP1//PKD2//TMX3//UGT1A10//UGT1A8//UGT1A7//UGT1A6//UGT1A9//UGT1A4//UGT1A1//UGT1A3//TMED9//CTSA//DNAJB12//TMEM100//RGMA//KLHL14//STIM2//RTN1//MSMO1//ELOVL4//WFS1//RNF128//PLEKHF2//ELOVL7//CAMLG//B3GNT5//PPP1R15B//B3GALT2//SQSTM1//ORMDL3//EDEM1//GJB2//RGMB//STX6//TRIM3//FUT9//ADCY3//STMN2//SCRG1//CLCN5//FGD4//RAB12//RAB3GAP1//GOLGA8A//MMD//GOLGA4//LDLR//MINK1//C11ORF24//FAM20C//PTHLH//PLEKHA3//FYCO1//KBTBD8//GNPTG//GLYR1//AP1S2//SGCE//CCDC126//PKDCC//TRIP10//MMGT1//ITM2B//CXCL14//LAPTM4A//RAB30//MAN2A1//TBC1D12//NRBF2//RAB23//ATG16L1//TBC1D17//EPHA5//AKAP11//TMEM135//DYNC1LI2//ARRDC3//SNX2//PPT2//LHX8//PEX5L//MARCKS//CLCN6//ZNRF2//MCOLN2//GNAQ//OSTM1//NSF//SPHK2//RHEB//GPR137B//VLDLR//CYBRD1//LAMTOR3//ZFYVE9//GNS//OGN//EDN1//CNIH1//GRIA1//MCFD2//RAP1B//C6ORF120//TTR//MTX3//SNN//MICU3//PRKAR2B//COX7A2//NLN//THOP1//FASTK//PABPC5//IMMP2L//SBF2//BDP1//KLF2//SMARCD2//KLF4//HAND2//RAD21//PGRMC2//NUP35//CNEP1R1//RNF180//NUP43//LBR//CLIP1//AGFG1//GDF11//TACC2//MAP3K2//DNAJB4//FZD10//KLF12//VPS37A//CRKL//PDIK1L//SH3D19//DCP2//E2F3//S1PR1//ELAVL2//EZH1//BAHD1//KDM2A//KLHDC10//FLII//USP24//MORC3//ST6GALNAC3//HBP1//NRG1//HOXA3//HOXD1//ZBTB34//NR3C2//PPP1R12A//KLF3//CTDSPL2//BNC2//CENPQ//FBXW7//ELMOD1//INTS8//PRKACB//FEM1C//REXO1//USP28//PTPN4//RGS3//SCN1A//ZMAT3//SHOX//BMPR2//STAT4//TGIF1//LDLRAD4//CENPO//ADAM12//LCOR//NUDCD1//CBFB//CDC23//BTAF1//CCNE2//WDR20//GIT2//RBM25//YPEL2//SLC14A1//TSG101//HAUS8//TMOD1//FAM118B//CFL2//TGFB1I1//HIPK3//DENND1B//MPP1//RANBP6//EPC2//BRMS1L//PIGA//ARL6IP1//MIA3//ANO8//WNT7A//KDELC1//GJC1//SEC23A//OSBPL5//PPM1L//RDH10//SPTSSA//REEP3//RAB21//SLC35D1//FLRT1//SAR1B//JKAMP//UGT1A5//XYLT2//SCD5//GSG1//SYT6//MAP3K5//SERINC3//HS3ST5//ARFIP1//ST8SIA5//ST6GALNAC6//RND3//ST8SIA3//SYBU//CHST7//PMEPA1//MARCH4//ST3GAL3//ST6GAL2//CHST1//ST3GAL5//DSEL//HS2ST1//HS3ST3A1//HS3ST1//FAM91A1//SNAP25//C16ORF70//STEAP2//CASD1//TMEM87A//MYO1B//KIF13A//CALN1//USP6NL//TRAPPC8//ARAP2//STX2//EPS15//C7ORF43//SLC2A4//CLIP4//CALD1// |
| GO:0043226 | organelle | Cellular component | 802 | 13597 | 970 | 18698 | 1.13698488675109 | 5.42462032250552e-14 | 9.31949771406448e-12 | 13.2656306533525 | 0.82680412371134 | ADCY3//DYNLL2//LCA5//PKD2//CEP41//NAPEPLD//DRD1//GDF11//CALCOCO2//OSBPL5//ARAP2//KLF6//VPS37A//DENND1B//DNMT3B//S1PR1//EGR2//CC2D1B//STX2//EPS15//MYCBP2//CIC//APPL1//GJA1//RAB30//GLRA1//GOLGA4//GRHL1//AGFG1//AGFG2//FOXN2//MIA3//RHOC//RND3//MEF2D//NOVA1//ACOX1//ERGIC2//UGT1A10//UGT1A8//UGT1A7//UGT1A6//UGT1A5//UGT1A9//NDFIP2//UGT1A4//UGT1A1//UGT1A3//PPARG//CTSA//C7ORF43//SYBU//SPHK2//PMEPA1//RBBP8//SHOX//SLC2A4//SLC14A1//HSPA13//TFAP2A//VBP1//LDLRAD4//EVI5//BAG6//FYCO1//CLIP4//UBA5//CALD1//RUNX3//SQSTM1//AP1S2//BTAF1//TRIP10//ZFYVE9//PPT2//ITM2B//CLOCK//GIT2//SLC6A14//EPS8//MSN//GPRC5C//SNAP25//AHNAK//CALM2//GPM6A//OGN//ARRDC4//ADAM10//CNN3//EPHA4//CPEB3//FMR1//NSMF//NPTN//GRIA1//NEFH//NSF//P2RY1//MINK1//PSD//CPEB1//BMPR2//BNIP3//SOS1//SPOCK1//STRN//LRP8//CPEB4//FXR1//BCL2L11//LYPLA1//ACOT7//FAM210A//CCDC58//DDX6//E2F1//ELK3//PIKFYVE//ACSL1//PPM1E//SLC44A1//RAB38//CLIC4//SACS//GLRX//GLUD2//SPRYD4//ZADH2//TCAIM//IDH1//COX8C//JARID2//BOLA3//CHCHD10//MMP2//ACO1//DCAF8//DNAJC27//PMAIP1//PON2//MIEF1//DUS2//PPP2CA//ARMC1//PPP3R1//PPP6C//MAPK1//PTEN//BCAT2//MOAP1//ACBD3//CAPRIN2//SOX4//TFAP2C//YWHAG//ZNF217//DCTPP1//APOO//UXS1//SLC25A32//DNAJC30//PHYHIPL//URI1//FAM110B//COX7A2L//BAG5//AKAP11//TMEM135//ACBD5//RANBP9//TOB1//TSHZ1//CDK6//TOPORS//CDK8//CDKN1A//HMG20A//IRF9//DLC1//ZBTB18//CEBPB//POLR3G//KHDRBS3//GNB5//PTGES3//TOB2//ZMYND11//ZNF268//TCERG1//COPS8//BTG3//WWP2//DUSP10//EGLN3//TADA1//NAA30//OSR1//CPEB2//MTPN//UBXN2B//PTPDC1//CRK//CRY2//SESN3//ZNF597//RBFOX3//CTNND2//PPP4R2//RNF38//CREBRF//ESCO2//ZNF782//NKX2-3//SPRED1//GADD45A//ZNF800//GLIS3//ARX//DMWD//DR1//DUSP1//DUSP2//DUSP5//E2F5//ZNF367//ZFC3H1//EMX2//EN1//ERG//ESR1//ETS1//CCNY//ZBTB9//FHL2//DZIP1//FOXJ3//FOXF1//FOXF2//FOXC1//WDFY3//PALLD//PDZD2//MYT1L//CDK19//FLI1//ZFPM2//CNOT6L//NR5A2//DNAJB5//TES//GATA6//LHX6//LATS2//RGS17//SESN1//GOLGA3//MKNK2//GRB2//ATAD2//GTF2E1//CNOT7//EPN1//PURG//SERTAD3//MDFIC//H2AFZ//HIVEP2//MNX1//HMGB3//HSBP1//SPOPL//ZBTB41//IMPDH1//IRF1//AR//JAK1//JUN//ZNF568//KDR//IPO5//USP27X//LHX1//MAF//MBNL1//MDM4//MEOX2//ATXN3//MLLT6//MYB//MYBL1//NAP1L3//NCK1//NEUROD1//NEUROG1//NF2//NFIA//NFE2//NFIB//NHLH2//NME1//NPAS2//NPAT//NR4A2//OTX2//SYCP3//PAX9//PBX3//MEMO1//RLIM//IER5//CDK17//ZBTB7A//TAOK3//YTHDF2//PIAS4//RASD1//ZFR//CMPK1//ARID4B//PFKP//PIK3CB//BRWD1//ERRFI1//SNTG1//RBM47//POU3F2//POU4F1//EGLN1//CCNJ//RSBN1//ESRP1//CC2D1A//SOBP//ZNF532//UBE2W//CDCA7L//PRKAA1//NPLOC4//RUFY2//BTBD7//CMTR2//TRERF1//DCAF6//SLC2A4RG//MDM1//PAK6//PHTF2//ZNFX1//ZNF630//ZNF287//CNOT6//ZNF512B//RANBP10//TSHZ3//ZBTB4//ZBTB26//MIER1//MID1IP1//RRAGD//RARB//ARID4A//RBBP7//PLEKHA1//RBL2//REST//REV3L//RNF2//RNF4//RPL15//ATXN1//DMRTA1//PRDM16//MAP2K4//SRSF2//SOX17//NKX2-4//GMNC//NABP1//RFX7//BCL11B//SKP1//UBE2Z//SNAPC1//SOX11//STAT3//BTF3//ZEB1//BTG1//TCF21//TEAD1//TEF//THRA//KLF10//TMF1//TSC1//UBE2I//UGP2//ZNF2//ZNF3//ZNF131//PTP4A1//BTG2//ZNF655//DEK//BHLHE41//NPEPL1//TBL1XR1//SNIP1//NR4A3//BRD3//KLHL15//FOSL1//HMGA2//SGPP1//ANP32E//TCF7L1//ARID5B//KIAA1109//BTBD10//PCGF5//MSANTD4//DPY30//MASTL//USP45//DGKE//ZIC5//BHLHE40//CDC14A//KLF7//RTCA//KAT2B//PER2//CCNC//FUBP3//CCND2//WASL//RPS6KA4//ZNF804A//RPS6KA5//STK17B//ZBTB47//ZFAND4//PLAA//CAMK2N2//HAND1//QKI//KIF23//PHF14//ST18//KMT2B//MFAP3L//USP3//RAB12//TBC1D12//NRBF2//RAB23//ATG16L1//PLEKHF2//TBC1D17//RTN3//CDS1//CRTAP//CKAP4//COL1A2//TBC1D20//FGD5//VKORC1L1//TMEM64//SMIM14//UBXN4//SULF1//LCLAT1//SERP1//ZDHHC1//ITGA5//FIBIN//SHISA2//LAMA3//LRP6//FAM19A1//MAN1A1//DNAJB9//ASPH//DERL2//IER3IP1//TMX3//TMED9//DNAJB12//TMEM100//RGMA//KLHL14//STIM2//RTN1//MSMO1//ELOVL4//WFS1//RNF128//ELOVL7//CAMLG//B3GNT5//PPP1R15B//B3GALT2//ORMDL3//EDEM1//EPHA5//GJB2//RGMB//STX6//TRIM3//FUT9//STMN2//SCRG1//CLCN5//FGD4//RAB3GAP1//GOLGA8A//MMD//LDLR//C11ORF24//FAM20C//PTHLH//PLEKHA3//KBTBD8//GNPTG//GLYR1//SGCE//CCDC126//PKDCC//MMGT1//CXCL14//LAPTM4A//MAN2A1//DSC2//TRAPPC8//FLRT1//DLL1//NTF3//CHMP3//CHIC1//USP6NL//CFL2//IQGAP2//WASF3//DNAJB4//FAM129A//COX7A2//CRKL//PPM1L//GPR180//DPYSL2//EFNA1//F3//ENPP4//RAB21//EFR3A//CD2AP//NPNT//PCOLCE2//GNAQ//GNS//SLC46A3//CD274//GSTA2//PSAT1//HPRT1//KRT27//ITGAV//ITGB8//C6ORF120//ARHGAP1//MARCKS//MYO1B//CLDN11//PFN2//SERPINI1//ARL15//UEVLD//TRPV6//NAGK//PRKACB//PRKAR2B//PLSCR4//RAB22A//RAP2C//PTPRG//RAP1B//RHEB//BMP3//SNX2//ACTC1//TSG101//TTR//WNT7A//PPCS//CYBRD1//PDCD1LG2//HDHD2//LAMTOR3//SLC4A4//ADAM9//CCT6A//SLIT2//SLK//DYNC1LI2//FBXO28//MTBP//NUP43//PPP1R12A//CLIP1//RAD21//YPEL2//FBXW7//ZMAT3//RDH10//LMLN//CETN1//NEDD1//CEP128//MZT1//MAP7D1//CDC42EP3//PTPN21//SLAIN1//FRMD6//DCX//KLHL3//MYLIP//TMOD3//ENAH//PTPN4//TGFB1I1//TMOD1//CCDC6//DCTN6//CENPO//CORO2B//KLHL2//ARHGAP6//LIMA1//CENPQ//TMEM63B//TWF1//TACC2//SLAIN2//MPP1//DCP2//NEFM//SESTD1//HECW2//DGKH//LPAR1//STEAP2//SNX5//ANKRD50//ARRDC3//EDN1//RAB9B//LHX8//ADRB1//TNF//PEX5L//IGF1//FCHO2//PAN3//TDRD5//CLCN6//ZNRF2//MCOLN2//OSTM1//GPR137B//VLDLR//CNIH1//MCFD2//SEC23A//SAR1B//FRY//HAUS8//SLC32A1//SYT6//SLC17A7//SLC17A6//C16ORF70//FLII//PXK//CEP97//REEP3//WDR47//RASSF3//KIF5A//KIF13A//CHRM2//LHFPL2//NHLRC2//TGFB2//VEGFB//CDC37L1//PLEKHB2//BDP1//SERINC3//HS3ST5//ST6GALNAC3//ARFIP1//ST8SIA5//ST6GALNAC6//ST8SIA3//CHST7//MARCH4//XYLT2//ST3GAL3//ST6GAL2//CHST1//ST3GAL5//DSEL//HS2ST1//HS3ST3A1//PIGA//BAHD1//KLF2//SMARCD2//KLF4//HAND2//PGRMC2//NUP35//CNEP1R1//RNF180//LBR//MAP3K2//FZD10//KLF12//PDIK1L//SH3D19//E2F3//ELAVL2//EZH1//KDM2A//KLHDC10//USP24//MORC3//HBP1//NRG1//HOXA3//HOXD1//ZBTB34//NR3C2//KLF3//CTDSPL2//BNC2//ELMOD1//INTS8//FEM1C//REXO1//USP28//RGS3//SCN1A//STAT4//TGIF1//ADAM12//LCOR//NUDCD1//CBFB//CDC23//CCNE2//WDR20//RBM25//MTX3//SNN//MICU3//NLN//THOP1//FASTK//PABPC5//SBF2//ARL6IP1//ANO8//KDELC1//GJC1//SPTSSA//SLC35D1//JKAMP//SCD5//GSG1//HS3ST1//FAM91A1//ZFYVE21//FAM118B//HIPK3//NECAP1//CASD1//FZD6//ULK2//RANBP6//TMEM87A//CALN1//EPC2//RAB28//MAPRE3//TMEM17//C5ORF30//IMMP2L//BRMS1L//MAP3K5// |
| GO:0005737 | cytoplasm | Cellular component | 706 | 11544 | 970 | 18698 | 1.17888598352516 | 7.03548431137433e-14 | 1.00724683724509e-11 | 13.1527060010696 | 0.727835051546392 | SPOCK1//FRY//EPS8//RHOC//RND3//MARCKS//TSC1//TRIP10//PKD2//KIF5A//MAPK1//MAP2K4//RAB21//SYBU//RASD1//DDX6//ATG16L1//FMR1//FXR1//GOLGA8A//CNIH1//ADAM10//STX6//RTN3//SEC23A//FUT9//SERINC3//CLCN5//PIKFYVE//RAB12//HS3ST5//ST6GALNAC3//GJA1//ARFIP1//RAB30//GOLGA3//GOLGA4//GRIA1//ST8SIA5//ST6GALNAC6//MIA3//MAN1A1//MAN2A1//NSF//ST8SIA3//NDFIP2//TMED9//PPP6C//CHST7//PMEPA1//MARCH4//RHEB//XYLT2//ACBD3//ST3GAL3//PLEKHA3//TMF1//APOO//B3GNT5//GNPTG//ST6GAL2//CHST1//B3GALT2//ST3GAL5//AP1S2//MCFD2//DSEL//MMGT1//ITM2B//HS2ST1//EDEM1//HS3ST3A1//EXOC5//NCK1//PPP1R15B//RPL15//NEDD1//CCNY//TRAPPC8//PLEKHF2//SQSTM1//ULK2//CALCOCO2//PIGA//VPS37A//TSG101//CHMP3//TOPORS//DCP2//PAN3//CNOT7//YTHDF2//CPEB1//PPP1R12A//DEK//DYNC1LI2//RAB38//LDLR//PON2//CTSA//ARRDC3//RRAGD//SNX2//FYCO1//PPT2//BCL2L11//LYPLA1//ACOT7//FAM210A//CCDC58//DYNLL2//E2F1//ELK3//ACSL1//PPM1E//SLC44A1//CLIC4//SACS//GLRX//GLUD2//SPRYD4//ZADH2//TCAIM//IDH1//COX8C//JARID2//BOLA3//CHCHD10//MMP2//NEFH//ACO1//P2RY1//DCAF8//ACOX1//DNAJC27//PMAIP1//MIEF1//DUS2//PPP2CA//ARMC1//PPP3R1//PTEN//BCAT2//MOAP1//CAPRIN2//BNIP3//SOX4//TFAP2C//YWHAG//ZNF217//DCTPP1//UXS1//SLC25A32//DNAJC30//PHYHIPL//URI1//FAM110B//COX7A2L//BAG5//RANBP9//HIPK3//FAM13A//CDK6//CDKN1A//IRF9//DLC1//TACC2//CDC42EP3//POLR3G//DCTN6//GNB5//PTGES3//MAP3K2//TOB2//IQGAP2//ZNF268//PDE10A//COPS8//CKAP4//WWP2//RAPGEF4//DNAJB4//AKAP11//DUSP10//EGLN3//KLHL2//KLF12//OSBPL5//FCHO2//FAM129A//ARAP2//TADA1//TAGAP//FGD4//NAA30//CNN3//OSR1//KLF6//MTPN//UBXN2B//CRK//CRKL//CRY2//HECTD2//SYT6//SH3D19//DENND5B//SPRED1//DENND1B//DCX//DPYSL2//EPS15//ESR1//RASGEF1A//NAPEPLD//ZNRF2//DZIP1//RAB3GAP1//FOXC1//WDFY3//PALLD//CDK19//FLI1//FLII//EFR3A//RGL1//UBXN4//OTUD3//DNAJC16//DOCK9//CNOT6L//LCLAT1//GAB1//CNEP1R1//DSTYK//DNAJB5//TMEM87A//NECAP1//APPL1//TES//STEAP2//FBXL5//KLHL3//LATS2//GJB2//SNX5//SERP1//SESN1//RASSF3//FAM126B//GRB2//OSTM1//MYLIP//GSTA2//GTF2E1//GRHL1//EPN1//PSAT1//MNX1//HPRT1//AGFG1//AGFG2//HSBP1//KRT27//NUP43//IMPDH1//IRF1//AR//ITGAV//JAK1//JUN//USP27X//ARHGAP1//ARHGAP6//MBNL1//MAP3K5//ATXN3//NR3C2//MPP1//MZT1//MSN//MTRR//NF2//NME1//NPAS2//MINK1//ARHGEF4//MEMO1//SAR1B//RLIM//RAB9B//LIMA1//PEX5L//RAB23//CMPK1//PFKP//PIK3CB//PLEK//BRWD1//ERRFI1//EGLN1//PPARG//CC2D1A//MTMR10//PXK//CENPQ//FBXW7//ELMOD1//CDCA7L//NAGK//PRKAA1//CDC37L1//NPLOC4//PRKACB//MAP7D1//ENAH//PRKAR2B//TRERF1//DCAF6//SPHK2//MDM1//FEM1C//PTHLH//CNOT6//RIMKLB//TWF1//KLHL14//SLAIN2//RANBP10//ZBTB4//RAP2C//MID1IP1//RAD21//RAP1B//ARID4A//RBBP7//PLEKHA1//RBL2//REST//RGS3//RRM2//CLIP1//ATXN1//PRDM16//SRSF2//ARHGEF28//AKTIP//NABP1//SKP1//SLC2A4//UBE2Z//MTMR9//SNAP25//SOS1//SOS2//STAT3//STAT4//HSPA13//BTF3//ZEB1//ACTC1//TFAP2A//TGFBR2//THOP1//THRA//TMOD1//UBE2I//UGP2//VBP1//EVI5//BTG2//AHNAK//ZFYVE21//BAG6//CENPO//RNF128//CEP97//PPCS//TBC1D17//SNIP1//UBA5//CALD1//DOCK5//CCDC6//FOSL1//DDHD1//SBF2//TCF7L1//FAM160A2//FBXO30//NSMAF//KBTBD8//GLYR1//NUDCD1//PPFIA1//PIK3R3//DGKE//RHPN2//CDC14A//RUNX3//CDC23//CCND2//WASL//LMLN//RPS6KA4//CCT6A//CCNE2//LONRF1//HAUS8//ZFYVE9//CAMK2N2//ARHGAP12//KIF23//CEP41//CLOCK//PPM1F//SOCS5//USP6NL//GIT2//G3BP2//JOSD1//GFPT2//TMEM135//ACBD5//EPHA4//MTX3//SNN//MICU3//COX7A2//NLN//FASTK//PABPC5//CLCN6//MMD//MCOLN2//GNAQ//GPR137B//VLDLR//CYBRD1//LAMTOR3//LAPTM4A//STMN2//DGKH//S1PR1//LPAR1//KDR//RUFY2//ANKRD50//ARRDC4//TRIM3//FGD5//ADRB1//LRP6//MYO1B//DERL2//RAB22A//PTP4A1//TBC1D12//NRBF2//CDS1//CRTAP//COL1A2//TBC1D20//VKORC1L1//TMEM64//SMIM14//PDZD2//SULF1//GLRA1//ZDHHC1//ITGA5//FIBIN//SHISA2//LAMA3//FAM19A1//DNAJB9//ASPH//IER3IP1//TMX3//UGT1A10//UGT1A8//UGT1A7//UGT1A6//UGT1A9//UGT1A4//UGT1A1//UGT1A3//DNAJB12//TMEM100//RGMA//PHTF2//STIM2//RTN1//MSMO1//ELOVL4//WFS1//ELOVL7//CPEB4//CAMLG//ORMDL3//ARL6IP1//FAM20C//ANO8//WNT7A//KDELC1//GJC1//PPM1L//RDH10//SPTSSA//DRD1//REEP3//SLC35D1//FLRT1//RNF180//ERGIC2//JKAMP//UGT1A5//SCD5//SGPP1//GSG1//EPHA5//RGMB//STK17B//ADCY3//SCRG1//ESCO2//C11ORF24//DPY30//SGCE//CCDC126//PKDCC//CXCL14//OGN//HS3ST1//FAM91A1//C16ORF70//EDN1//CD2AP//CETN1//CEP128//TMOD3//NEFM//STX2//NTF3//KIF13A//CALM2//CFL2//FHL2//SCN1A//CASD1//LBR//SLC32A1//SLC17A7//SLC17A6//NPNT//GPRC5C//FZD6//CHRM2//NSMF//LHFPL2//IGF1//NHLRC2//TGFB2//VEGFB//DSC2//RGS17//DLL1//CHIC1//ANP32E//KAT2B//LDLRAD4//CALN1//TDRD5//SERPINI1//GNS//C6ORF120//TTR//USP3//NR4A3//IMMP2L//NANOS1//INHBB//STK33//PER2//SLK//TNF//PLEKHB2//PSD//ENPP4//IQCJ-SCHIP1//TOB1//CORO2B//CEBPB//WASF3//BTG3//PTPN21//FZD10//PCMTD1//SLAIN1//FRMD6//LYPD6//CPEB2//PTPDC1//SESN3//RBFOX3//CTNND2//PPP4R2//GADD45A//LCA5//DNMT3B//DUSP1//DUSP2//DUSP5//E2F5//EGR2//EPHA2//EPHA7//ERG//ETS1//KCTD20//CPEB3//WDR47//MAPRE3//KLHDC10//LRCH1//ZFPM2//NR5A2//BAMBI//ZDHHC5//RANBP6//GLRB//NKIRAS1//MKNK2//MDFIC//SCHIP1//NRG1//HMGB3//SPOPL//RBM44//PTAR1//IPO5//MAF//MEF2D//MEOX2//MET//NEUROD1//NFE2//NPAT//NR4A2//PAIP2//IER5//CDK17//TAOK3//LGSN//PIAS4//ZFR//ARID4B//PFN2//SNTG1//ANKIB1//ZFAND6//POU4F1//PPP1R12C//ASPN//RALGPS2//UBE2W//CMTR2//LMO3//SLC2A4RG//PAK6//HECW2//MIER1//PTPN4//RBM25//RARB//RNF4//AIDA//BMPR2//SOX11//STRN//BTG1//TGFB1I1//ZNF655//NPEPL1//VASH2//BTBD10//MASTL//USP45//UNK//BHLHE40//FUBP3//UBE3B//CCNG2//C5ORF30//ZNF804A//RPS6KA5//KLF4//SLIT2//ZFAND4//RAB28//PLAA//HAND1//QKI//FEZ2//FNIP1//MFAP3L// |
| GO:0043227 | membrane-bounded organelle | Cellular component | 754 | 12616 | 970 | 18698 | 1.15205466467062 | 3.26069283336747e-13 | 4.0013359198038e-11 | 12.486690111055 | 0.777319587628866 | GDF11//CALCOCO2//OSBPL5//ARAP2//KLF6//VPS37A//DENND1B//DNMT3B//S1PR1//EGR2//CC2D1B//STX2//EPS15//MYCBP2//CIC//APPL1//GJA1//RAB30//GLRA1//GOLGA4//GRHL1//AGFG1//AGFG2//FOXN2//MIA3//RHOC//RND3//MEF2D//NOVA1//ACOX1//ERGIC2//UGT1A10//UGT1A8//UGT1A7//UGT1A6//UGT1A5//UGT1A9//NDFIP2//UGT1A4//UGT1A1//UGT1A3//PPARG//CTSA//C7ORF43//SYBU//SPHK2//PMEPA1//RBBP8//SHOX//SLC2A4//SLC14A1//HSPA13//TFAP2A//VBP1//LDLRAD4//EVI5//BAG6//FYCO1//CLIP4//UBA5//CALD1//RUNX3//SQSTM1//AP1S2//BTAF1//TRIP10//ZFYVE9//PPT2//ITM2B//CLOCK//GIT2//RDH10//SLC6A14//EPS8//MSN//GPRC5C//SNAP25//AHNAK//CALM2//BCL2L11//LYPLA1//ACOT7//FAM210A//CCDC58//DYNLL2//DDX6//E2F1//ELK3//PIKFYVE//ACSL1//PPM1E//SLC44A1//RAB38//CLIC4//SACS//GLRX//GLUD2//SPRYD4//ZADH2//TCAIM//IDH1//COX8C//JARID2//BOLA3//CHCHD10//MMP2//NEFH//ACO1//P2RY1//DCAF8//DNAJC27//PMAIP1//PON2//MIEF1//DUS2//PPP2CA//ARMC1//PPP3R1//PPP6C//MAPK1//PTEN//BCAT2//MOAP1//ACBD3//CAPRIN2//BNIP3//SOX4//TFAP2C//YWHAG//ZNF217//DCTPP1//APOO//UXS1//SLC25A32//DNAJC30//PHYHIPL//URI1//FAM110B//COX7A2L//BAG5//RANBP9//TOB1//TSHZ1//ADAM10//CDK6//TOPORS//CDK8//CDKN1A//HMG20A//IRF9//DLC1//ZBTB18//CEBPB//POLR3G//KHDRBS3//GNB5//PTGES3//TOB2//ZMYND11//ZNF268//TCERG1//COPS8//BTG3//WWP2//DUSP10//EGLN3//TADA1//NAA30//OSR1//CPEB2//MTPN//UBXN2B//PTPDC1//CRK//CRY2//SESN3//ZNF597//RBFOX3//CTNND2//PPP4R2//RNF38//CREBRF//ESCO2//ZNF782//NKX2-3//SPRED1//GADD45A//ZNF800//GLIS3//ARX//DMWD//DR1//DRD1//DUSP1//DUSP2//DUSP5//E2F5//ZNF367//ZFC3H1//EMX2//EN1//ERG//ESR1//ETS1//CCNY//ZBTB9//FHL2//CPEB3//DZIP1//FOXJ3//FOXF1//FOXF2//FOXC1//WDFY3//PALLD//PDZD2//MYT1L//CDK19//FLI1//FMR1//ZFPM2//CNOT6L//NR5A2//DNAJB5//NSMF//TES//GATA6//LHX6//LATS2//RGS17//SESN1//GOLGA3//MKNK2//GRB2//ATAD2//GTF2E1//CNOT7//EPN1//PURG//SERTAD3//MDFIC//H2AFZ//HIVEP2//MNX1//HMGB3//HSBP1//SPOPL//ZBTB41//IMPDH1//IRF1//AR//JAK1//JUN//ZNF568//KDR//IPO5//USP27X//LHX1//MAF//MBNL1//MDM4//MEOX2//ATXN3//MLLT6//MYB//MYBL1//NAP1L3//NCK1//NEUROD1//NEUROG1//NF2//NFIA//NFE2//NFIB//NHLH2//NME1//NPAS2//NPAT//NR4A2//OTX2//SYCP3//PAX9//PBX3//MEMO1//RLIM//IER5//CDK17//ZBTB7A//TAOK3//YTHDF2//PIAS4//RASD1//ZFR//CMPK1//ARID4B//PFKP//PIK3CB//BRWD1//ERRFI1//SNTG1//RBM47//POU3F2//POU4F1//EGLN1//CCNJ//RSBN1//ESRP1//CC2D1A//SOBP//ZNF532//UBE2W//CDCA7L//PRKAA1//NPLOC4//RUFY2//BTBD7//CMTR2//TRERF1//DCAF6//SLC2A4RG//MDM1//PAK6//PHTF2//ZNFX1//ZNF630//ZNF287//CNOT6//ZNF512B//RANBP10//TSHZ3//ZBTB4//ZBTB26//MIER1//MID1IP1//RRAGD//RARB//ARID4A//RBBP7//PLEKHA1//RBL2//REST//REV3L//RNF2//RNF4//RPL15//ATXN1//DMRTA1//PRDM16//MAP2K4//SRSF2//SOX17//NKX2-4//CPEB1//GMNC//NABP1//RFX7//BCL11B//SKP1//UBE2Z//SNAPC1//SOX11//STAT3//BTF3//ZEB1//BTG1//TCF21//TEAD1//TEF//THRA//KLF10//TMF1//TSC1//UBE2I//UGP2//ZNF2//ZNF3//ZNF131//PTP4A1//BTG2//ZNF655//DEK//BHLHE41//NPEPL1//TBL1XR1//SNIP1//NR4A3//BRD3//KLHL15//CPEB4//FOSL1//FXR1//HMGA2//SGPP1//ANP32E//TCF7L1//ARID5B//KIAA1109//BTBD10//PCGF5//MSANTD4//DPY30//MASTL//USP45//DGKE//ZIC5//BHLHE40//CDC14A//KLF7//RTCA//KAT2B//PER2//CCNC//FUBP3//CCND2//WASL//RPS6KA4//ACBD5//ZNF804A//RPS6KA5//STK17B//ZBTB47//ZFAND4//PLAA//CAMK2N2//HAND1//QKI//KIF23//PHF14//ST18//KMT2B//MFAP3L//USP3//RTN3//CDS1//CRTAP//CKAP4//COL1A2//TBC1D20//FGD5//VKORC1L1//TMEM64//SMIM14//EPHA4//UBXN4//SULF1//LCLAT1//SERP1//ZDHHC1//ITGA5//FIBIN//SHISA2//LAMA3//LRP6//FAM19A1//MAN1A1//DNAJB9//ASPH//DERL2//IER3IP1//PKD2//TMX3//TMED9//DNAJB12//TMEM100//RGMA//KLHL14//STIM2//RTN1//MSMO1//ELOVL4//WFS1//RNF128//PLEKHF2//ELOVL7//CAMLG//B3GNT5//PPP1R15B//B3GALT2//ORMDL3//EDEM1//GJB2//RGMB//STX6//TRIM3//FUT9//ADCY3//STMN2//SCRG1//CLCN5//FGD4//RAB12//RAB3GAP1//GOLGA8A//MMD//LDLR//MINK1//C11ORF24//FAM20C//PTHLH//PLEKHA3//KBTBD8//GNPTG//GLYR1//SGCE//CCDC126//PKDCC//MMGT1//CXCL14//LAPTM4A//MAN2A1//GPM6A//OGN//ARRDC4//DYNC1LI2//ARRDC3//SNX2//TBC1D12//NRBF2//RAB23//ATG16L1//TBC1D17//LHX8//EPHA5//AKAP11//TMEM135//DSC2//TRAPPC8//FLRT1//DLL1//NTF3//CHMP3//CHIC1//USP6NL//CFL2//IQGAP2//WASF3//DNAJB4//FAM129A//COX7A2//CRKL//PPM1L//GPR180//DPYSL2//EFNA1//F3//NAPEPLD//ENPP4//RAB21//EFR3A//CD2AP//NPNT//PCOLCE2//GNAQ//GNS//SLC46A3//CD274//GSTA2//PSAT1//HPRT1//KRT27//ITGAV//ITGB8//C6ORF120//ARHGAP1//MARCKS//MYO1B//NSF//CLDN11//PFN2//SERPINI1//ARL15//UEVLD//TRPV6//NAGK//PRKACB//PRKAR2B//PLSCR4//RAB22A//RAP2C//PTPRG//RAP1B//RHEB//BMP3//ACTC1//TSG101//TTR//WNT7A//PPCS//CYBRD1//PDCD1LG2//HDHD2//LAMTOR3//SLC4A4//ADAM9//CCT6A//SLIT2//SLK//PEX5L//DGKH//LPAR1//STEAP2//SNX5//ANKRD50//CLIP1//EDN1//RAB9B//ADRB1//GRIA1//TNF//IGF1//FCHO2//CLCN6//ZNRF2//MCOLN2//OSTM1//GPR137B//VLDLR//CNIH1//MCFD2//SEC23A//SAR1B//SLC32A1//SYT6//SLC17A7//SLC17A6//C16ORF70//CHRM2//LHFPL2//NHLRC2//TGFB2//VEGFB//CDC37L1//PLEKHB2//SERINC3//HS3ST5//ST6GALNAC3//ARFIP1//ST8SIA5//ST6GALNAC6//ST8SIA3//CHST7//MARCH4//XYLT2//ST3GAL3//ST6GAL2//CHST1//ST3GAL5//DSEL//HS2ST1//HS3ST3A1//LBR//MTX3//SNN//MICU3//SBF2//KIF13A//ZFYVE21//FZD6//ULK2//NUP35//CNEP1R1//RANBP6//TMEM87A//CALN1//TMEM17//PGRMC2//RNF180//NUP43//FAM91A1//NLN//THOP1//FASTK//PABPC5//IMMP2L//BDP1//KLF2//SMARCD2//KLF4//HAND2//RAD21//TACC2//MAP3K2//FZD10//KLF12//PDIK1L//SH3D19//DCP2//E2F3//ELAVL2//EZH1//BAHD1//KDM2A//KLHDC10//FLII//USP24//MORC3//HBP1//NRG1//HOXA3//HOXD1//ZBTB34//NR3C2//PPP1R12A//KLF3//CTDSPL2//BNC2//CENPQ//FBXW7//ELMOD1//INTS8//FEM1C//REXO1//USP28//PTPN4//RGS3//SCN1A//ZMAT3//BMPR2//STAT4//TGIF1//CENPO//ADAM12//LCOR//NUDCD1//CBFB//CDC23//CCNE2//WDR20//RBM25//YPEL2//HAUS8//TMOD1//FAM118B//TGFB1I1//HIPK3//MPP1//EPC2//BRMS1L//PSD//PIGA//ARL6IP1//ANO8//KDELC1//GJC1//SPTSSA//REEP3//SLC35D1//JKAMP//SCD5//GSG1//MAP3K5//HS3ST1//CASD1//NECAP1// |
| GO:0044464 | cell part | Cellular component | 927 | 16886 | 970 | 18698 | 1.05822098706792 | 3.09920488992252e-10 | 3.18696455616285e-08 | 9.50874971139501 | 0.955670103092784 | RANBP9//GJC1//LHFPL2//ADAM10//STX6//RTN3//SEMA4B//TACC2//CDC42EP3//GNB5//IQGAP2//ADCY3//SERINC3//CKAP4//RAPGEF4//DNAJB4//AKAP11//SLC6A14//CHRM2//CHRM5//PALM2//FCHO2//FAM129A//CLCN5//FRMD6//CMKLR1//NUP35//LYPD6//RAET1E//CRK//SLC32A1//DYNLL2//SYT6//SH3D19//ADRB1//DGKH//SPRED1//ABHD3//DPYSL2//DRD1//DSC2//S1PR1//LPAR1//EFNA1//EFNA5//EPHA2//EPHA4//EPHA5//EPHA7//STX2//EPS8//EPS15//ESR1//F3//ACSL1//AMER2//CCNY//ZNRF2//NTNG1//SLITRK3//ENPP4//WDFY3//PALLD//LRCH1//EFR3A//SULF1//SLC44A1//LPAR3//CD2AP//RAB38//FLRT1//CADM2//SLC41A1//MCOLN2//SLC24A2//BAMBI//ZDHHC5//CLIC4//NECAP1//LRIG1//TES//STEAP2//RGS17//GJA1//GJB2//TNFRSF21//GLRA1//GLRB//GNAQ//GPM6A//CDH20//RASSF3//DLL1//FAM126B//RGMB//GRB2//GRIA1//MYLIP//CD274//EPN1//ST6GALNAC6//IGF1//ITGA6//AR//ITGA5//ITGAV//ITGB8//KCNA4//KCNJ3//KCNJ10//KDR//RHOC//RND3//LDLR//LRP6//MARCKS//MET//ATXN3//MMP2//MYO1B//ASPH//MSN//NCK1//NF2//NSF//CLDN11//P2RY1//ACOX1//RAB9B//TAOK3//LIMA1//LGSN//CHMP3//RASD1//RAB23//PIK3CB//PKD2//CHIC1//C11ORF24//KCNK10//TREM1//PON2//TMX3//CC2D1A//PXK//RALGPS2//XKR8//PPP2CA//C7ORF43//TMEM100//TMEM63B//TRPV6//PRKACB//LMBR1L//ENAH//PRKAR2B//MAPK1//PMEPA1//RGMA//SLC17A7//PLSCR4//PTEN//PTGER4//RAB22A//SLC24A3//DSCAML1//SLC12A5//NLN//ARRDC3//STIM2//ANO8//RAP2C//RAP1B//ENPP5//ARID4A//NTN4//PLEKHA1//RGS3//HCN2//MSMO1//SCN1A//ARHGEF28//ZMAT3//AKTIP//SLC2A4//SLC6A1//SLC6A6//SLC14A1//SLC20A1//BMPR2//CAPRIN2//SNAP25//SOS1//STAT3//HSPA13//TGFBR2//TNF//TRPC3//TSC1//TSG101//FAM155A//VLDLR//WNT7A//LRP8//AHNAK//ADIPOR2//CYBRD1//CALD1//DOCK5//ADAM12//PDCD1LG2//CALM2//SGPP1//FZD6//PCDH11Y//CALN1//RECK//TMEM25//DGKE//OR6A2//LAMTOR3//SLC4A4//SGCE//WASL//ZNF804A//ARRDC4//STK17B//MMGT1//SLIT2//RAB28//ORMDL3//ITM2B//USP6NL//MFAP3L//JOSD1//EREG//ARL5A//RNF180//IL2//MPP1//ARL15//PRKAA1//CCL2//STK33//TGFB1I1//LONRF3//B3GNT5//CDC23//LONRF1//LRP12//AP1S2//FZD10//NPTN//ADAM9//BCL2L11//PGRMC2//RAB12//RAB21//DOCK9//RAB30//DNAJC27//RTN1//EVI5//KDELC1//TBC1D17//FRY//TOPORS//EXOC5//MAPRE3//KIF13A//KIF23//SNX5//SNX2//SLK//NPLOC4//FMR1//AGFG1//TRIP10//NEFH//NME1//YWHAG//RDH10//NRG1//ACTC1//NEDD1//ACVR1//EDN1//INHBB//USP3//RAB3GAP1//PPP3R1//IER5//STRN//PPP1R15B//PPP4R2//CNEP1R1//PPP1R12A//STMN2//OTX2//TSHZ3//CPEB4//MZT1//PSD//MASTL//CNN3//KIF5A//SERPINI1//KLHL14//TGFB2//FXR1//WWP2//FBXL5//ANKIB1//RNF2//ZFC3H1//CDK6//CDKN1A//CCNC//CCND2//WFS1//IQCJ-SCHIP1//TOB1//CALCOCO2//IRF9//CORO2B//DLC1//LYPLA1//CEBPB//TRIM3//PTGES3//MAP3K2//TOB2//ZNF268//WASF3//BTG3//PTPN21//DUSP10//EGLN3//KLHL2//PCMTD1//ARAP2//FGD4//SLAIN1//NAA30//CPEB2//PTPDC1//HECTD2//SESN3//RBFOX3//CTNND2//FGD5//GADD45A//DDX6//DCP2//LCA5//DNMT3B//DUSP1//DUSP2//DUSP5//E2F5//EGR2//ERG//ETS1//NAPEPLD//KCTD20//CPEB3//DZIP1//WDR47//KLHDC10//PDZD2//OTUD3//ZFPM2//CNOT6L//NR5A2//DSTYK//NSMF//APPL1//SACS//RANBP6//ARFIP1//SESN1//GOLGA3//GOLGA4//NKIRAS1//MKNK2//CNOT7//PSAT1//MDFIC//SCHIP1//NRBF2//HMGB3//HPRT1//AGFG2//HSBP1//SPOPL//NANOS1//IDH1//IMPDH1//IRF1//JAK1//RBM44//PTAR1//IPO5//ARHGAP1//ARHGAP6//MAF//MBNL1//DNAJB9//MEF2D//MEOX2//NEUROD1//NFE2//ACO1//NPAT//NR4A2//MINK1//DCAF8//MEMO1//PAIP2//CDK17//ERGIC2//PIAS4//ZFR//CMPK1//ARID4B//PFKP//PFN2//PLEK//ERRFI1//SNTG1//ZFAND6//POU4F1//EGLN1//NDFIP2//PPP1R12C//ASPN//MTMR10//ARMC1//UBE2W//FBXW7//CDC37L1//RUFY2//CMTR2//LMO3//SLC2A4RG//PAK6//PTHLH//RIMKLB//HECW2//TWF1//MIER1//PTPN4//RBM25//RRAGD//RARB//RNF4//ATXN1//MOAP1//MAP2K4//CPEB1//AIDA//SKP1//UBE2Z//MTMR9//BNIP3//SOX4//SOX11//SPOCK1//BTG1//UBE2I//UGP2//VBP1//PTP4A1//BTG2//ZNF655//BAG6//NPEPL1//VASH2//UBA5//UXS1//DDHD1//SNN//BTBD10//NSMAF//PHYHIPL//PPFIA1//USP45//UNK//BHLHE40//CDC14A//RUNX3//URI1//PER2//SQSTM1//FUBP3//RPS6KA4//UBE3B//CCNG2//C5ORF30//CCT6A//RPS6KA5//KLF4//HAUS8//ZFAND4//PLAA//HAND1//QKI//FEZ2//FNIP1//SOCS5//GIT2//G3BP2//FOXF1//FOXF2//GATA6//JUN//NHLH2//NPAS2//PBX3//POU3F2//TRERF1//RBL2//SOX17//ZEB1//TEAD1//NR4A3//TCF7L1//HAND2//CLOCK//BAG5//TRAPPC8//CNOT6//HMGA2//ARHGAP12//MAP3K5//CD69//VANGL1//RNF38//NTF3//DCX//SLC17A6//BCL11B//FOSL1//C16ORF70//CETN1//PDE10A//DMWD//NEUROG1//SCRG1//NEFM//FLII//RRM2//KLHL3//KLHL15//KBTBD8//DCAF6//CCNE2//SNAPC1//E2F3//PPARG//THRA//PRDM16//EDEM1//MTPN//TMEM185A//NFIB//LBR//MICU3//SLC25A32//COX7A2L//APCDD1//MMD//GPR6//UGT1A1//GPRC5C//PRRG1//PTPRG//GPR137B//ST3GAL5//SLC16A6//ARHGEF4//UBXN4//NUP43//CLIP1//MTX3//PMAIP1//MIEF1//SYBU//JARID2//DPY30//KMT2B//EZH1//RBBP7//REV3L//CEP41//DYNC1LI2//FBXO28//MTBP//KAT2B//DEK//ACOT7//FAM210A//CCDC58//E2F1//ELK3//PIKFYVE//PPM1E//GLRX//GLUD2//SPRYD4//ZADH2//TCAIM//COX8C//BOLA3//CHCHD10//DUS2//PPP6C//BCAT2//ACBD3//TFAP2C//ZNF217//DCTPP1//APOO//DNAJC30//FAM110B//SERP1//RPL15//TSHZ1//CDK8//HMG20A//ZBTB18//POLR3G//KHDRBS3//ZMYND11//TCERG1//COPS8//TADA1//OSR1//KLF6//UBXN2B//CRY2//ZNF597//CREBRF//ESCO2//ZNF782//NKX2-3//ZNF800//GLIS3//ARX//DR1//ZNF367//CC2D1B//EMX2//EN1//ZBTB9//FHL2//FOXJ3//FOXC1//MYT1L//MYCBP2//CDK19//FLI1//CIC//DNAJB5//LHX6//LATS2//ATAD2//GTF2E1//GRHL1//PURG//SERTAD3//H2AFZ//HIVEP2//MNX1//FOXN2//ZBTB41//ZNF568//USP27X//LHX1//MDM4//MLLT6//MYB//MYBL1//NAP1L3//NFIA//NOVA1//SYCP3//PAX9//RLIM//ZBTB7A//YTHDF2//BRWD1//RBM47//CCNJ//RSBN1//ESRP1//SOBP//ZNF532//CDCA7L//BTBD7//MDM1//PHTF2//ZNFX1//ZNF630//ZNF287//ZNF512B//RANBP10//ZBTB4//ZBTB26//MID1IP1//RBBP8//REST//DMRTA1//SRSF2//NKX2-4//GMNC//NABP1//RFX7//BTF3//TCF21//TEF//TFAP2A//KLF10//TMF1//ZNF2//ZNF3//ZNF131//BHLHE41//TBL1XR1//SNIP1//BRD3//ANP32E//ARID5B//KIAA1109//PCGF5//MSANTD4//ZIC5//KLF7//RTCA//ACBD5//ZBTB47//CAMK2N2//PHF14//ST18//RAD21//YPEL2//CDS1//CRTAP//COL1A2//TBC1D20//VKORC1L1//TMEM64//SMIM14//LCLAT1//ZDHHC1//FIBIN//SHISA2//LAMA3//FAM19A1//MAN1A1//DERL2//IER3IP1//UGT1A10//UGT1A8//UGT1A7//UGT1A6//UGT1A9//UGT1A4//UGT1A3//TMED9//CTSA//DNAJB12//ELOVL4//RNF128//PLEKHF2//ELOVL7//CAMLG//B3GALT2//GDF11//OSBPL5//VPS37A//DENND1B//MIA3//UGT1A5//SPHK2//SHOX//LDLRAD4//FYCO1//CLIP4//BTAF1//ZFYVE9//PPT2//FUT9//GOLGA8A//FAM20C//PLEKHA3//GNPTG//GLYR1//CCDC126//PKDCC//CXCL14//LAPTM4A//MAN2A1//TMEM135//LMLN//CEP128//MAP7D1//TMOD3//TMOD1//CCDC6//ULK2//ATG16L1//HIPK3//FAM13A//SEC23A//DCTN6//KLF12//TAGAP//CRKL//DENND5B//RASGEF1A//RGL1//DNAJC16//GAB1//PAN3//TMEM87A//OSTM1//GSTA2//KRT27//NR3C2//MTRR//SAR1B//PEX5L//CENPQ//ELMOD1//NAGK//FEM1C//SLAIN2//RHEB//SOS2//STAT4//THOP1//ZFYVE21//CENPO//CEP97//PPCS//SBF2//FAM160A2//FBXO30//NUDCD1//PIK3R3//RHPN2//PPM1F//GFPT2//FASTK//PABPC5//OGN//HS3ST1//CNIH1//MCFD2//GNS//HS3ST5//ST6GALNAC3//ST8SIA5//ST8SIA3//CHST7//MARCH4//XYLT2//ST3GAL3//ST6GAL2//CHST1//DSEL//HS2ST1//HS3ST3A1//PIGA//ANKRD50//TBC1D12//PPM1L//SPTSSA//REEP3//SLC35D1//ARL6IP1//JKAMP//SCD5//GSG1//CFL2//NPNT//TDRD5//BAHD1//PDIK1L//ELAVL2//KDM2A//USP24//MORC3//HBP1//HOXA3//HOXD1//ZBTB34//KLF3//CTDSPL2//BNC2//INTS8//BDP1//REXO1//USP28//SMARCD2//TGIF1//LCOR//CBFB//WDR20//C6ORF120//TTR//TMEM17//EPC2//KLF2//LHX8//CLCN6//SESTD1//CASD1//FAM91A1//NHLRC2//VEGFB//COX7A2//BRMS1L//FAM118B//IMMP2L//PLEKHB2// |
| GO:0005623 | cell | Cellular component | 928 | 16915 | 970 | 18698 | 1.05754631252076 | 3.33907811472243e-10 | 3.18696455616285e-08 | 9.47637342089888 | 0.956701030927835 | RANBP9//GJC1//LHFPL2//ADAM10//STX6//RTN3//SEMA4B//TACC2//CDC42EP3//GNB5//IQGAP2//ADCY3//SERINC3//CKAP4//RAPGEF4//DNAJB4//AKAP11//SLC6A14//CHRM2//CHRM5//PALM2//FCHO2//FAM129A//CLCN5//FRMD6//CMKLR1//NUP35//LYPD6//RAET1E//CRK//SLC32A1//DYNLL2//SYT6//SH3D19//ADRB1//DGKH//SPRED1//ABHD3//DPYSL2//DRD1//DSC2//S1PR1//LPAR1//EFNA1//EFNA5//EPHA2//EPHA4//EPHA5//EPHA7//STX2//EPS8//EPS15//ESR1//F3//ACSL1//AMER2//CCNY//ZNRF2//NTNG1//SLITRK3//ENPP4//WDFY3//PALLD//LRCH1//EFR3A//SULF1//SLC44A1//LPAR3//CD2AP//RAB38//FLRT1//CADM2//SLC41A1//MCOLN2//SLC24A2//BAMBI//ZDHHC5//CLIC4//NECAP1//LRIG1//TES//STEAP2//RGS17//GJA1//GJB2//TNFRSF21//GLRA1//GLRB//GNAQ//GPM6A//CDH20//RASSF3//DLL1//FAM126B//RGMB//GRB2//GRIA1//MYLIP//CD274//EPN1//ST6GALNAC6//IGF1//ITGA6//AR//ITGA5//ITGAV//ITGB8//KCNA4//KCNJ3//KCNJ10//KDR//RHOC//RND3//LDLR//LRP6//MARCKS//MET//ATXN3//MMP2//MYO1B//ASPH//MSN//NCK1//NF2//NSF//CLDN11//P2RY1//ACOX1//RAB9B//TAOK3//LIMA1//LGSN//CHMP3//RASD1//RAB23//PIK3CB//PKD2//CHIC1//C11ORF24//KCNK10//TREM1//PON2//TMX3//CC2D1A//PXK//RALGPS2//XKR8//PPP2CA//C7ORF43//TMEM100//TMEM63B//TRPV6//PRKACB//LMBR1L//ENAH//PRKAR2B//MAPK1//PMEPA1//RGMA//SLC17A7//PLSCR4//PTEN//PTGER4//RAB22A//SLC24A3//DSCAML1//SLC12A5//NLN//ARRDC3//STIM2//ANO8//RAP2C//RAP1B//ENPP5//ARID4A//NTN4//PLEKHA1//RGS3//HCN2//MSMO1//SCN1A//ARHGEF28//ZMAT3//AKTIP//SLC2A4//SLC6A1//SLC6A6//SLC14A1//SLC20A1//BMPR2//CAPRIN2//SNAP25//SOS1//STAT3//HSPA13//TGFBR2//TNF//TRPC3//TSC1//TSG101//FAM155A//VLDLR//WNT7A//LRP8//AHNAK//ADIPOR2//CYBRD1//CALD1//DOCK5//ADAM12//PDCD1LG2//CALM2//SGPP1//FZD6//PCDH11Y//CALN1//RECK//TMEM25//DGKE//OR6A2//LAMTOR3//SLC4A4//SGCE//WASL//ZNF804A//ARRDC4//STK17B//MMGT1//SLIT2//RAB28//ORMDL3//ITM2B//USP6NL//MFAP3L//JOSD1//TRERF1//ZNF217//TBL1XR1//TADA1//KAT2B//BDP1//GOLGA8A//CNIH1//SEC23A//FUT9//PIKFYVE//RAB12//HS3ST5//ST6GALNAC3//ARFIP1//RAB30//GOLGA3//GOLGA4//ST8SIA5//MIA3//MAN1A1//MAN2A1//ST8SIA3//NDFIP2//TMED9//PPP6C//SYBU//CHST7//MARCH4//RHEB//XYLT2//ACBD3//ST3GAL3//PLEKHA3//TMF1//APOO//B3GNT5//GNPTG//ST6GAL2//CHST1//B3GALT2//ST3GAL5//AP1S2//MCFD2//DSEL//HS2ST1//EDEM1//HS3ST3A1//EXOC5//TOPORS//WWP2//FBXL5//ANKIB1//RNF2//IER5//STRN//PPP1R15B//ZFC3H1//RPL15//ZBTB18//JUN//HMGA2//NEDD1//EREG//ARL5A//RNF180//IL2//MPP1//ARL15//PRKAA1//CCL2//STK33//TGFB1I1//LONRF3//CDC23//LONRF1//CDK6//CDKN1A//CCNC//CCND2//TRAPPC8//PLEKHF2//SQSTM1//ULK2//CALCOCO2//ATG16L1//WFS1//PIGA//FMR1//SYCP3//CENPQ//RAD21//DYNC1LI2//FBXO28//MTBP//NUP43//PPP1R12A//CLIP1//DCTN6//CENPO//CEBPB//PTGES3//DPY30//NABP1//ESCO2//BAHD1//MAF//RBL2//H2AFZ//GLYR1//KLF2//POLR3G//E2F1//IRF1//MEF2D//POU4F1//SMARCD2//RUNX3//KLF4//HAND2//USP3//DDX6//UBE2I//PCGF5//ANP32E//VPS37A//FRY//CETN1//CEP128//LATS2//CDC14A//HAUS8//MZT1//DCP2//PAN3//CNOT7//YTHDF2//CPEB1//KLF6//E2F5//SESN1//NFIB//CDCA7L//PAK6//BTBD10//LHX8//BCL2L11//KRT27//NME1//KLHL2//FGD4//FGD5//SNX5//TRIP10//ARHGAP12//DEK//CTSA//RRAGD//SNX2//FYCO1//PPT2//LYPLA1//ACOT7//FAM210A//CCDC58//ELK3//PPM1E//SACS//GLRX//GLUD2//SPRYD4//ZADH2//TCAIM//IDH1//COX8C//JARID2//BOLA3//CHCHD10//NEFH//ACO1//DCAF8//DNAJC27//PMAIP1//MIEF1//DUS2//ARMC1//PPP3R1//BCAT2//MOAP1//BNIP3//SOX4//TFAP2C//YWHAG//DCTPP1//UXS1//SLC25A32//DNAJC30//PHYHIPL//URI1//FAM110B//COX7A2L//BAG5//HIPK3//FAM13A//IRF9//DLC1//MAP3K2//TOB2//ZNF268//PDE10A//COPS8//DUSP10//EGLN3//KLF12//OSBPL5//ARAP2//TAGAP//NAA30//CNN3//OSR1//MTPN//UBXN2B//CRKL//CRY2//HECTD2//DENND5B//DENND1B//DCX//RASGEF1A//NAPEPLD//DZIP1//RAB3GAP1//FOXC1//RAB21//CDK19//FLI1//FLII//RGL1//UBXN4//OTUD3//DNAJC16//DOCK9//CNOT6L//LCLAT1//GAB1//CNEP1R1//DSTYK//DNAJB5//TMEM87A//APPL1//KLHL3//SERP1//OSTM1//GSTA2//GTF2E1//GRHL1//PSAT1//MNX1//HPRT1//AGFG1//AGFG2//HSBP1//IMPDH1//JAK1//KIF5A//USP27X//ARHGAP1//ARHGAP6//MBNL1//MAP3K5//NR3C2//MTRR//NPAS2//MINK1//ARHGEF4//MEMO1//SAR1B//RLIM//PEX5L//CMPK1//PFKP//PLEK//BRWD1//ERRFI1//EGLN1//PPARG//MTMR10//FBXW7//ELMOD1//NAGK//CDC37L1//NPLOC4//MAP7D1//DCAF6//SPHK2//MDM1//FEM1C//PTHLH//CNOT6//RIMKLB//TWF1//KLHL14//SLAIN2//RANBP10//ZBTB4//MID1IP1//RBBP7//REST//RRM2//ATXN1//PRDM16//MAP2K4//SRSF2//SKP1//UBE2Z//MTMR9//SOS2//STAT4//BTF3//ZEB1//ACTC1//TFAP2A//THOP1//THRA//TMOD1//UGP2//VBP1//EVI5//BTG2//ZFYVE21//BAG6//RNF128//CEP97//PPCS//TBC1D17//SNIP1//UBA5//CCDC6//FOSL1//DDHD1//FXR1//SBF2//TCF7L1//FAM160A2//FBXO30//NSMAF//KBTBD8//NUDCD1//PPFIA1//PIK3R3//RHPN2//LMLN//RPS6KA4//CCT6A//CCNE2//ZFYVE9//CAMK2N2//KIF23//CEP41//CLOCK//PPM1F//SOCS5//GIT2//G3BP2//GFPT2//TMEM135//ACBD5//FZD10//APCDD1//MMD//GPR6//LRP12//NRG1//UGT1A1//GPRC5C//PRRG1//PTPRG//GPR137B//ACVR1//SLC16A6//CD69//TOB1//TSHZ1//CDK8//HMG20A//KHDRBS3//ZMYND11//TCERG1//BTG3//CPEB2//PTPDC1//SESN3//ZNF597//RBFOX3//CTNND2//PPP4R2//RNF38//CREBRF//ZNF782//NKX2-3//GADD45A//ZNF800//GLIS3//ARX//DMWD//DNMT3B//DR1//DUSP1//DUSP2//DUSP5//ZNF367//EGR2//CC2D1B//EMX2//EN1//ERG//ETS1//ZBTB9//FHL2//CPEB3//FOXJ3//FOXF1//FOXF2//PDZD2//MYT1L//MYCBP2//CIC//ZFPM2//NR5A2//NSMF//GATA6//LHX6//MKNK2//ATAD2//PURG//SERTAD3//MDFIC//HIVEP2//HMGB3//FOXN2//SPOPL//ZBTB41//ZNF568//IPO5//LHX1//MDM4//MEOX2//MLLT6//MYB//MYBL1//NAP1L3//NEUROD1//NEUROG1//NFIA//NFE2//NHLH2//NOVA1//NPAT//NR4A2//OTX2//PAX9//PBX3//CDK17//ERGIC2//ZBTB7A//PIAS4//ZFR//ARID4B//SNTG1//RBM47//POU3F2//CCNJ//RSBN1//ESRP1//SOBP//ZNF532//UBE2W//RUFY2//BTBD7//CMTR2//SLC2A4RG//PHTF2//ZNFX1//ZNF630//ZNF287//ZNF512B//TSHZ3//ZBTB26//MIER1//RARB//RBBP8//REV3L//RNF4//DMRTA1//SOX17//NKX2-4//GMNC//RFX7//BCL11B//SNAPC1//SOX11//BTG1//TCF21//TEAD1//TEF//KLF10//ZNF2//ZNF3//ZNF131//PTP4A1//ZNF655//BHLHE41//NPEPL1//NR4A3//BRD3//KLHL15//CPEB4//ARID5B//KIAA1109//MSANTD4//MASTL//USP45//ZIC5//BHLHE40//KLF7//RTCA//PER2//FUBP3//RPS6KA5//ZBTB47//ZFAND4//PLAA//HAND1//QKI//PHF14//ST18//KMT2B//PGRMC2//LBR//IQCJ-SCHIP1//CORO2B//TRIM3//WASF3//STMN2//PTPN21//PCMTD1//SLAIN1//RDH10//LCA5//EDN1//KCTD20//WDR47//MAPRE3//KLHDC10//RANBP6//NKIRAS1//SCHIP1//NRBF2//NANOS1//RBM44//PTAR1//DNAJB9//PAIP2//PFN2//ZFAND6//PPP1R12C//ASPN//LMO3//HECW2//PTPN4//RBM25//AIDA//SPOCK1//VASH2//SNN//UNK//UBE3B//CCNG2//C5ORF30//FEZ2//FNIP1//SLK//GDF11//PDIK1L//E2F3//ELAVL2//EZH1//KDM2A//USP24//MORC3//HBP1//HOXA3//HOXD1//ZBTB34//KLF3//CTDSPL2//BNC2//INTS8//REXO1//USP28//SHOX//TGIF1//LDLRAD4//LCOR//CBFB//BTAF1//WDR20//YPEL2//MTX3//MICU3//COX7A2//FASTK//PABPC5//CLCN6//LAPTM4A//ANKRD50//DERL2//TBC1D12//CDS1//CRTAP//COL1A2//TBC1D20//VKORC1L1//TMEM64//SMIM14//ZDHHC1//FIBIN//SHISA2//LAMA3//FAM19A1//IER3IP1//UGT1A10//UGT1A8//UGT1A7//UGT1A6//UGT1A9//UGT1A4//UGT1A3//DNAJB12//RTN1//ELOVL4//ELOVL7//CAMLG//ARL6IP1//FAM20C//KDELC1//PPM1L//SPTSSA//REEP3//SLC35D1//JKAMP//UGT1A5//SCD5//GSG1//CLIP4//SCRG1//CCDC126//PKDCC//CXCL14//OGN//HS3ST1//FAM91A1//C16ORF70//KIF13A//TMOD3//NEFM//NTF3//NPTN//ADAM9//PSD//FAM118B//CFL2//VANGL1//SLC17A6//CASD1//TGFB2//TMEM185A//NPNT//NHLRC2//VEGFB//EPC2//TDRD5//SERPINI1//GNS//C6ORF120//TTR//TMEM17//IMMP2L//SESTD1//INHBB//PLEKHB2//BRMS1L//CCL8// |
| GO:0044444 | cytoplasmic part | Cellular component | 588 | 9626 | 970 | 18698 | 1.17748366216069 | 2.61941975758853e-09 | 2.25008157176855e-07 | 8.5817949010413 | 0.606185567010309 | NCK1//PPP1R15B//CCNY//TRAPPC8//PLEKHF2//SQSTM1//ULK2//ATG16L1//GJA1//PPP1R12A//DEK//BCL2L11//LYPLA1//ACOT7//FAM210A//CCDC58//DYNLL2//DDX6//E2F1//ELK3//PIKFYVE//ACSL1//PPM1E//SLC44A1//RAB38//CLIC4//SACS//GLRX//GLUD2//SPRYD4//ZADH2//TCAIM//IDH1//COX8C//JARID2//BOLA3//CHCHD10//MMP2//NEFH//ACO1//P2RY1//DCAF8//ACOX1//DNAJC27//PMAIP1//PON2//MIEF1//NDFIP2//DUS2//PPP2CA//ARMC1//PPP3R1//PPP6C//MAPK1//PTEN//BCAT2//MOAP1//ACBD3//CAPRIN2//BNIP3//SOX4//TFAP2C//YWHAG//ZNF217//DCTPP1//APOO//UXS1//SLC25A32//DNAJC30//PHYHIPL//URI1//FAM110B//COX7A2L//BAG5//RANBP9//HIPK3//FAM13A//CDK6//STX6//CALCOCO2//CDKN1A//IRF9//DLC1//SEC23A//TACC2//CDC42EP3//POLR3G//EXOC5//DCTN6//GNB5//PTGES3//MAP3K2//TOB2//IQGAP2//ZNF268//PDE10A//COPS8//CKAP4//WWP2//RAPGEF4//DNAJB4//AKAP11//DUSP10//EGLN3//KLHL2//KLF12//OSBPL5//FCHO2//FAM129A//ARAP2//TADA1//TAGAP//CLCN5//NEDD1//FGD4//NAA30//CNN3//OSR1//KLF6//MTPN//VPS37A//UBXN2B//CRK//CRKL//CRY2//HECTD2//SYT6//SH3D19//DENND5B//SPRED1//DENND1B//DCX//DCP2//DYNC1LI2//DPYSL2//RAB12//EPS15//ESR1//RASGEF1A//NAPEPLD//ZNRF2//DZIP1//RAB3GAP1//FOXC1//WDFY3//RAB21//GOLGA8A//PALLD//CDK19//FLI1//FLII//EFR3A//RGL1//UBXN4//OTUD3//FMR1//DNAJC16//DOCK9//CNOT6L//LCLAT1//GAB1//CNEP1R1//PAN3//DSTYK//DNAJB5//TMEM87A//NECAP1//APPL1//TES//STEAP2//FBXL5//KLHL3//LATS2//GJB2//SNX5//SERP1//ARFIP1//SESN1//GOLGA3//GOLGA4//RASSF3//FAM126B//GRB2//OSTM1//MYLIP//GSTA2//GTF2E1//GRHL1//CNOT7//EPN1//PSAT1//MNX1//HPRT1//AGFG1//AGFG2//HSBP1//KRT27//NUP43//IMPDH1//IRF1//AR//ITGAV//JAK1//JUN//KIF5A//RHOC//USP27X//ARHGAP1//ARHGAP6//MAN1A1//MBNL1//MAP3K5//ATXN3//NR3C2//MPP1//MZT1//MSN//MTRR//NF2//NME1//NPAS2//NSF//MINK1//ARHGEF4//MEMO1//SAR1B//RLIM//RAB9B//YTHDF2//LIMA1//PEX5L//CHMP3//RAB23//CMPK1//PFKP//PIK3CB//PLEK//BRWD1//ERRFI1//EGLN1//PPARG//CC2D1A//MTMR10//PXK//CENPQ//FBXW7//ELMOD1//CDCA7L//NAGK//PRKAA1//CDC37L1//NPLOC4//PRKACB//MAP7D1//ENAH//PRKAR2B//TRERF1//DCAF6//SPHK2//MDM1//FEM1C//PTHLH//CNOT6//RIMKLB//TWF1//KLHL14//SLAIN2//RANBP10//ZBTB4//RAP2C//MID1IP1//RRAGD//RAD21//RAP1B//ARID4A//RBBP7//PLEKHA1//RBL2//REST//RGS3//RHEB//RPL15//RRM2//CLIP1//ATXN1//PRDM16//MAP2K4//SRSF2//ARHGEF28//AKTIP//CPEB1//NABP1//SKP1//SLC2A4//UBE2Z//MTMR9//SNAP25//SNX2//SOS1//SOS2//STAT3//STAT4//HSPA13//BTF3//ZEB1//ACTC1//TFAP2A//TGFBR2//THOP1//THRA//TMF1//TMOD1//TSC1//TSG101//UBE2I//UGP2//VBP1//EVI5//BTG2//AHNAK//ZFYVE21//BAG6//CENPO//RNF128//CEP97//PPCS//TBC1D17//SNIP1//UBA5//CALD1//DOCK5//CCDC6//FOSL1//DDHD1//FXR1//SBF2//TCF7L1//FAM160A2//FBXO30//NSMAF//KBTBD8//GLYR1//NUDCD1//PPFIA1//PIK3R3//DGKE//RHPN2//CDC14A//RUNX3//CDC23//AP1S2//CCND2//WASL//LMLN//RPS6KA4//CCT6A//CCNE2//LONRF1//TRIP10//HAUS8//ZFYVE9//CAMK2N2//ARHGAP12//KIF23//CEP41//CLOCK//PPM1F//SOCS5//USP6NL//GIT2//G3BP2//JOSD1//GFPT2//RTN3//CDS1//CRTAP//COL1A2//TBC1D20//FGD5//VKORC1L1//TMEM64//SMIM14//EPHA4//PDZD2//SULF1//GLRA1//ZDHHC1//ITGA5//KDR//FIBIN//SHISA2//LAMA3//LRP6//FAM19A1//DNAJB9//ASPH//DERL2//IER3IP1//PKD2//TMX3//UGT1A10//UGT1A8//UGT1A7//UGT1A6//UGT1A9//UGT1A4//UGT1A1//UGT1A3//TMED9//CTSA//DNAJB12//TMEM100//RGMA//PHTF2//STIM2//RTN1//MSMO1//ELOVL4//WFS1//PTP4A1//ELOVL7//CPEB4//CAMLG//B3GNT5//B3GALT2//ORMDL3//EDEM1//RGMB//STK17B//ADAM10//TRIM3//FUT9//ADCY3//STMN2//SCRG1//ESCO2//MMD//LDLR//ERGIC2//C11ORF24//FAM20C//PLEKHA3//FYCO1//GNPTG//DPY30//SGCE//CCDC126//PKDCC//MMGT1//ITM2B//CXCL14//LAPTM4A//NEFM//STX2//DSC2//FLRT1//RGS17//DLL1//NRBF2//RND3//NTF3//CHIC1//SYBU//ANP32E//CNIH1//GRIA1//MCFD2//USP3//SERINC3//EPHA5//CD2AP//NANOS1//INHBB//MYO1B//RASD1//STK33//CALN1//PER2//SLK//TMOD3//NPNT//TBC1D12//DGKH//S1PR1//LPAR1//RUFY2//ANKRD50//ARRDC3//ARRDC4//EDN1//TDRD5//TMEM135//ACBD5//NLN//FASTK//PABPC5//HS3ST5//ST6GALNAC3//RAB30//ST8SIA5//ST6GALNAC6//MIA3//MAN2A1//ST8SIA3//CHST7//PMEPA1//MARCH4//XYLT2//ST3GAL3//ST6GAL2//CHST1//ST3GAL5//DSEL//HS2ST1//HS3ST3A1//OGN//WNT7A//HS3ST1//PIGA//ANO8//KDELC1//GJC1//PPM1L//RDH10//SPTSSA//DRD1//REEP3//SLC35D1//ARL6IP1//RNF180//JKAMP//UGT1A5//SCD5//SGPP1//GSG1//GPRC5C//FZD6//SERPINI1//GNS//NSMF//CALM2//CFL2//FHL2//SCN1A//KAT2B//TOPORS//CETN1//CEP128//FRY//EPS8//MARCKS//KIF13A//PPT2//NR4A3//ADRB1//RAB22A//TNF//CLCN6//MCOLN2//GNAQ//GPR137B//VLDLR//CYBRD1//LAMTOR3//C6ORF120//TTR//IGF1//CHRM2//LHFPL2//SLC17A7//ENPP4//LBR//CASD1//MTX3//SNN//MICU3//COX7A2//FAM91A1//C16ORF70//SLC32A1//SLC17A6//LDLRAD4//PLEKHB2//PSD//IMMP2L//NHLRC2//TGFB2//VEGFB// |
| GO:0005829 | cytosol | Cellular component | 337 | 5022 | 970 | 18698 | 1.29353032225219 | 1.73531506519269e-08 | 1.35512331000047e-06 | 7.76062166286026 | 0.347422680412371 | RPL15//FYCO1//CCT6A//RRM2//ADCY3//SNAP25//TSC1//BAG6//BCL2L11//RANBP9//HIPK3//FAM13A//CDK6//STX6//CALCOCO2//CDKN1A//IRF9//DLC1//LYPLA1//SEC23A//TACC2//CDC42EP3//POLR3G//EXOC5//DCTN6//GNB5//PTGES3//MAP3K2//TOB2//IQGAP2//ZNF268//PDE10A//COPS8//CKAP4//WWP2//RAPGEF4//DNAJB4//AKAP11//DUSP10//EGLN3//KLHL2//KLF12//ACOT7//OSBPL5//FCHO2//FAM129A//ARAP2//TADA1//TAGAP//CLCN5//NEDD1//FGD4//NAA30//CNN3//OSR1//KLF6//MTPN//VPS37A//UBXN2B//CRK//CRKL//DYNLL2//CRY2//HECTD2//SYT6//SH3D19//DENND5B//SPRED1//DENND1B//DCX//DDX6//DCP2//DYNC1LI2//DPYSL2//PIKFYVE//RAB12//EPS15//ESR1//RASGEF1A//NAPEPLD//ZNRF2//DZIP1//TRAPPC8//RAB3GAP1//FOXC1//WDFY3//RAB21//GOLGA8A//PALLD//CDK19//FLI1//FLII//EFR3A//RGL1//UBXN4//OTUD3//FMR1//DNAJC16//DOCK9//RAB38//CNOT6L//LCLAT1//GAB1//CNEP1R1//PAN3//DSTYK//DNAJB5//CLIC4//TMEM87A//NECAP1//APPL1//TES//STEAP2//FBXL5//KLHL3//LATS2//GJA1//GJB2//SNX5//SERP1//ARFIP1//SESN1//GLRX//GOLGA3//GOLGA4//RASSF3//FAM126B//GRB2//OSTM1//MYLIP//GSTA2//GTF2E1//GRHL1//CNOT7//EPN1//PSAT1//MNX1//HPRT1//AGFG1//AGFG2//HSBP1//IDH1//KRT27//NUP43//IMPDH1//IRF1//AR//ITGAV//JAK1//JUN//KIF5A//RHOC//USP27X//ARHGAP1//ARHGAP6//MAN1A1//MBNL1//MAP3K5//ATXN3//NR3C2//MPP1//MZT1//MSN//MTRR//PPP1R12A//NCK1//NF2//ACO1//NME1//NPAS2//NSF//MINK1//ARHGEF4//DCAF8//MEMO1//SAR1B//RLIM//RAB9B//YTHDF2//LIMA1//PEX5L//CHMP3//RAB23//CMPK1//PFKP//PIK3CB//PLEK//PMAIP1//BRWD1//ERRFI1//EGLN1//PPARG//CC2D1A//MTMR10//PXK//DUS2//ATG16L1//PPP2CA//CENPQ//FBXW7//PPP3R1//PPP6C//ELMOD1//CDCA7L//NAGK//PRKAA1//CDC37L1//NPLOC4//PRKACB//MAP7D1//ENAH//PRKAR2B//TRERF1//DCAF6//MAPK1//SPHK2//MDM1//FEM1C//PTEN//PTHLH//CNOT6//RIMKLB//TWF1//KLHL14//SLAIN2//RANBP10//ZBTB4//RAP2C//MID1IP1//RRAGD//RAD21//RAP1B//ARID4A//RBBP7//PLEKHA1//RBL2//REST//RGS3//RHEB//CLIP1//ATXN1//PRDM16//MOAP1//MAP2K4//SRSF2//ARHGEF28//AKTIP//CPEB1//NABP1//SKP1//SLC2A4//UBE2Z//CAPRIN2//MTMR9//SNX2//SOS1//SOS2//STAT3//STAT4//HSPA13//BTF3//ZEB1//ACTC1//TFAP2A//TFAP2C//TGFBR2//THOP1//THRA//TMF1//TMOD1//TSG101//UBE2I//UGP2//VBP1//YWHAG//EVI5//BTG2//AHNAK//ZFYVE21//DCTPP1//APOO//CENPO//RNF128//CEP97//PPCS//TBC1D17//SNIP1//UBA5//CALD1//DOCK5//CCDC6//FOSL1//DDHD1//FXR1//SBF2//TCF7L1//FAM160A2//FBXO30//DNAJC30//NSMAF//KBTBD8//GLYR1//NUDCD1//PPFIA1//PIK3R3//DGKE//RHPN2//CDC14A//RUNX3//CDC23//URI1//SQSTM1//AP1S2//CCND2//WASL//LMLN//RPS6KA4//FAM110B//CCNE2//LONRF1//TRIP10//HAUS8//ZFYVE9//CAMK2N2//ARHGAP12//KIF23//BAG5//CEP41//CLOCK//PPM1F//SOCS5//ULK2//USP6NL//GIT2//G3BP2//JOSD1//GFPT2// |
| GO:0005634 | nucleus | Cellular component | 459 | 7267 | 970 | 18698 | 1.21753357573213 | 2.35984796780976e-08 | 1.68925783695715e-06 | 7.62711597536384 | 0.47319587628866 | LHX8//MARCKS//TRERF1//ZNF217//TBL1XR1//TADA1//KAT2B//BDP1//ZBTB18//JUN//HMGA2//NABP1//KLF2//CEBPB//POLR3G//E2F1//ESR1//H2AFZ//IRF1//AR//MEF2D//POU4F1//SMARCD2//STAT3//RUNX3//KLF4//HAND2//USP3//SYCP3//UBE2I//RAD21//ANP32E//KLF6//E2F5//SESN1//NFIB//CC2D1A//CDCA7L//PAK6//BTBD10//RNF2//PGRMC2//NUP35//WDFY3//UBXN4//CNEP1R1//NSMF//RNF180//NUP43//LBR//CLIP1//BNIP3//AGFG1//IPO5//CDK6//TOPORS//GDF11//STX6//CDK8//CDKN1A//IRF9//TACC2//KHDRBS3//PTGES3//MAP3K2//ZMYND11//TCERG1//COPS8//DNAJB4//FZD10//DUSP10//EGLN3//KLF12//ACOT7//VPS37A//PTPDC1//CRKL//PDIK1L//PPP4R2//RNF38//SH3D19//CREBRF//ESCO2//GADD45A//DCP2//DNMT3B//DUSP5//E2F3//S1PR1//ZNF367//EGR2//ELAVL2//CC2D1B//ELK3//ETS1//EZH1//FHL2//DZIP1//BAHD1//FOXC1//KDM2A//KLHDC10//FLII//CIC//FMR1//USP24//ZFPM2//SLC44A1//MORC3//NR5A2//ST6GALNAC3//GATA6//HBP1//GJA1//GOLGA3//MKNK2//GRB2//ATAD2//GTF2E1//GRHL1//NRBF2//NRG1//HIVEP2//HOXA3//HOXD1//HSBP1//JARID2//ZBTB34//MBNL1//MDM4//ATXN3//NR3C2//MYB//PPP1R12A//NEUROD1//NFIA//NFE2//NPAS2//NPAT//NR4A2//DCAF8//ACOX1//RLIM//KLF3//CTDSPL2//PIAS4//ARID4B//BRWD1//PPARG//CTSA//BNC2//ESRP1//CENPQ//UBE2W//FBXW7//PPP3R1//ELMOD1//PRKAA1//INTS8//NPLOC4//PRKACB//DCAF6//MAPK1//FEM1C//PTEN//PTHLH//REXO1//TSHZ3//USP28//ZBTB4//MIER1//PTPN4//RRAGD//RARB//ARID4A//RBBP7//RBBP8//PLEKHA1//RBL2//REST//REV3L//RGS3//RNF4//ATXN1//SCN1A//PRDM16//SRSF2//SOX17//ZMAT3//CPEB1//SHOX//SKP1//UBE2Z//BMPR2//SNAPC1//SOX4//STAT4//ZEB1//TEAD1//TFAP2A//TFAP2C//TGIF1//THRA//LDLRAD4//ZNF131//DEK//BAG6//CENPO//SNIP1//NR4A3//ADAM12//FOSL1//SGPP1//TCF7L1//ARID5B//PCGF5//LCOR//GLYR1//DPY30//MASTL//NUDCD1//USP45//CDC14A//RTCA//CBFB//CDC23//URI1//SQSTM1//CCNC//CCND2//RPS6KA4//BTAF1//CCNE2//ACBD5//WDR20//RPS6KA5//TRIP10//HAND1//KIF23//CLOCK//KMT2B//GIT2//DR1//RBM25//RHEB//AKAP11//DDX6//PPM1E//SERTAD3//MDFIC//MNX1//YPEL2//DNAJB9//NF2//NOVA1//ERGIC2//CMPK1//PIK3CB//SLC14A1//TSG101//ZNF655//FXR1//PER2//COX7A2L//HAUS8//TMOD1//FAM118B//CFL2//CLIC4//TGFB1I1//APPL1//CDK19//HIPK3//FLI1//CNOT7//BHLHE40//CKAP4//CRY2//DENND1B//MEOX2//MPP1//SLC2A4RG//TBC1D20//DUSP2//RANBP6//GNAQ//DNAJB12//EPC2//BRMS1L//RANBP9//TOB1//TSHZ1//ADAM10//CALCOCO2//HMG20A//DLC1//GNB5//TOB2//ZNF268//BTG3//WWP2//NAA30//OSR1//CPEB2//MTPN//UBXN2B//CRK//DYNLL2//SESN3//ZNF597//RBFOX3//CTNND2//ZNF782//NKX2-3//SPRED1//ZNF800//GLIS3//ARX//DMWD//DRD1//DUSP1//ZFC3H1//EMX2//EN1//ERG//CCNY//ZBTB9//CPEB3//FOXJ3//FOXF1//FOXF2//PALLD//PDZD2//MYT1L//MYCBP2//CNOT6L//DNAJB5//TES//SACS//LHX6//LATS2//RGS17//GLRX//SPRYD4//EPN1//PURG//HMGB3//AGFG2//FOXN2//SPOPL//ZBTB41//IMPDH1//JAK1//ZNF568//KDR//RHOC//USP27X//LHX1//CHCHD10//MAF//MLLT6//MMP2//MSN//MYBL1//NAP1L3//NCK1//NEUROG1//NHLH2//NME1//OTX2//PAX9//PBX3//MEMO1//DNAJC27//IER5//CDK17//ZBTB7A//TAOK3//YTHDF2//RASD1//ZFR//PFKP//PMAIP1//ERRFI1//SNTG1//PON2//RBM47//POU3F2//EGLN1//CCNJ//RSBN1//SOBP//PPP2CA//ARMC1//ZNF532//RUFY2//BTBD7//CMTR2//MDM1//PHTF2//ZNFX1//ZNF630//ZNF287//CNOT6//ZNF512B//RANBP10//ZBTB26//MID1IP1//RPL15//DMRTA1//MAP2K4//NKX2-4//GMNC//RFX7//BCL11B//CAPRIN2//SOX11//HSPA13//BTF3//BTG1//TCF21//TEF//KLF10//TMF1//TSC1//UGP2//VBP1//ZNF2//ZNF3//PTP4A1//EVI5//BTG2//AHNAK//DCTPP1//BHLHE41//NPEPL1//UBA5//BRD3//KLHL15//CPEB4//CALM2//KIAA1109//MSANTD4//DGKE//ZIC5//KLF7//FUBP3//WASL//ZNF804A//STK17B//ZBTB47//ZFAND4//PLAA//CAMK2N2//QKI//BAG5//PHF14//ST18//MFAP3L// |
| GO:0044446 | intracellular organelle part | Cellular component | 547 | 9080 | 970 | 18698 | 1.161247786003 | 3.16768057407124e-07 | 2.09310585625169e-05 | 6.49925861872157 | 0.563917525773196 | REV3L//SNX5//GOLGA3//PLEKHF2//PGRMC2//NUP35//WDFY3//UBXN4//CNEP1R1//NSMF//RNF180//NUP43//LBR//CLIP1//BNIP3//UBE2I//CHCHD10//NLN//THOP1//DYNC1LI2//FBXO28//MTBP//PPP1R12A//KAT2B//ESCO2//BAHD1//MAF//RAD21//RBL2//CCND2//H2AFZ//GLYR1//ZBTB18//JUN//HMGA2//AGFG1//IPO5//CDK6//TOPORS//GDF11//STX6//CDK8//CDKN1A//IRF9//CEBPB//TACC2//POLR3G//KHDRBS3//PTGES3//MAP3K2//ZMYND11//TCERG1//COPS8//DNAJB4//FZD10//DUSP10//EGLN3//KLF12//ACOT7//TADA1//VPS37A//PTPDC1//CRKL//PDIK1L//PPP4R2//RNF38//SH3D19//CREBRF//GADD45A//DCP2//DNMT3B//DUSP5//E2F1//E2F3//E2F5//S1PR1//ZNF367//EGR2//ELAVL2//CC2D1B//ELK3//ESR1//ETS1//EZH1//FHL2//DZIP1//FOXC1//KDM2A//KLHDC10//FLII//CIC//FMR1//USP24//ZFPM2//SLC44A1//MORC3//NR5A2//ST6GALNAC3//GATA6//HBP1//GJA1//MKNK2//GRB2//ATAD2//GTF2E1//GRHL1//NRBF2//NRG1//HIVEP2//HOXA3//HOXD1//HSBP1//IRF1//AR//JARID2//ZBTB34//MBNL1//MDM4//MEF2D//ATXN3//NR3C2//MYB//NEUROD1//NFIA//NFE2//NPAS2//NPAT//NR4A2//DCAF8//ACOX1//RLIM//KLF3//CTDSPL2//PIAS4//ARID4B//BRWD1//POU4F1//PPARG//CTSA//BNC2//ESRP1//CENPQ//UBE2W//FBXW7//PPP3R1//ELMOD1//PRKAA1//INTS8//NPLOC4//PRKACB//TRERF1//BDP1//DCAF6//MAPK1//FEM1C//PTEN//PTHLH//REXO1//TSHZ3//USP28//ZBTB4//MIER1//PTPN4//RRAGD//RARB//ARID4A//RBBP7//RBBP8//PLEKHA1//REST//RGS3//RNF2//RNF4//ATXN1//SCN1A//PRDM16//SRSF2//SOX17//ZMAT3//CPEB1//SHOX//NABP1//SKP1//UBE2Z//BMPR2//SMARCD2//SNAPC1//SOX4//STAT3//STAT4//ZEB1//TEAD1//TFAP2A//TFAP2C//TGIF1//THRA//LDLRAD4//ZNF131//ZNF217//DEK//BAG6//CENPO//TBL1XR1//SNIP1//NR4A3//ADAM12//FOSL1//SGPP1//TCF7L1//ARID5B//PCGF5//LCOR//DPY30//MASTL//NUDCD1//USP45//CDC14A//RTCA//RUNX3//CBFB//CDC23//URI1//SQSTM1//CCNC//RPS6KA4//BTAF1//CCNE2//ACBD5//WDR20//RPS6KA5//KLF4//TRIP10//HAND1//KIF23//CLOCK//KMT2B//GIT2//USP3//RBM25//RHEB//AKAP11//KLF6//DDX6//PPM1E//SERTAD3//MDFIC//MNX1//YPEL2//DNAJB9//NF2//NOVA1//ERGIC2//CMPK1//PIK3CB//CDCA7L//SLC14A1//TSG101//ZNF655//FXR1//PER2//COX7A2L//HAUS8//TMOD1//CFL2//CLIC4//TGFB1I1//TBC1D20//DUSP2//RANBP6//GNAQ//DNAJB12//FASTK//PABPC5//BCAT2//TMEM135//FRY//CETN1//NEDD1//CEP128//LATS2//PPP2CA//CALM2//PALLD//ATG16L1//LCA5//YTHDF2//CC2D1A//PXK//C7ORF43//EVI5//CEP97//FAM110B//MZT1//MAP7D1//PTP4A1//KBTBD8//TMOD3//RANBP9//DCX//LRP8//MTPN//DYNLL2//MYO1B//CALD1//WASL//DLC1//IQGAP2//CD2AP//PKD2//MARCKS//KIF5A//RAB28//CNIH1//ADAM10//RTN3//SEC23A//FUT9//SERINC3//CLCN5//PIKFYVE//RAB12//HS3ST5//ARFIP1//RAB30//GOLGA4//GRIA1//ST8SIA5//ST6GALNAC6//MIA3//RND3//MAN1A1//MAN2A1//NSF//ST8SIA3//NDFIP2//TMED9//PPP6C//SYBU//CHST7//PMEPA1//MARCH4//XYLT2//ACBD3//ST3GAL3//PLEKHA3//TMF1//APOO//B3GNT5//GNPTG//ST6GAL2//CHST1//B3GALT2//ST3GAL5//AP1S2//MCFD2//DSEL//MMGT1//ITM2B//HS2ST1//EDEM1//HS3ST3A1//OGN//WNT7A//HS3ST1//GOLGA8A//TRAPPC8//PIGA//DERL2//CRTAP//CKAP4//COL1A2//FAM20C//ANO8//WFS1//KDELC1//GJC1//CDS1//OSBPL5//PPM1L//VKORC1L1//RDH10//SPTSSA//DRD1//SMIM14//ACSL1//REEP3//RAB3GAP1//RAB21//SLC35D1//ARL6IP1//FLRT1//LCLAT1//SERP1//SHISA2//ASPH//IER3IP1//SAR1B//JKAMP//TMX3//UGT1A10//UGT1A8//UGT1A7//UGT1A6//UGT1A5//UGT1A9//UGT1A4//UGT1A1//UGT1A3//KLHL14//STIM2//MSMO1//SCD5//ELOVL7//GSG1//SYT6//MAP3K5//EPS15//ZNRF2//GPRC5C//SLC2A4//YWHAG//FZD6//ULK2//SERPINI1//IDH1//GNS//IMPDH1//SBF2//TMEM87A//UXS1//KIF13A//CALN1//USP6NL//DCTN6//KLF2//HAND2//CALCOCO2//CLCN6//MMD//MCOLN2//OSTM1//SPHK2//GPR137B//VLDLR//AHNAK//CYBRD1//LAMTOR3//ZFYVE9//LAPTM4A//C6ORF120//TTR//PPT2//PEX5L//EDN1//NME1//RAB23//PRKAR2B//MDM1//SLAIN2//CAPRIN2//CAMK2N2//CEP41//HECW2//BCL2L11//RPL15//NECAP1//RAB9B//ORMDL3//CHRM2//LDLR//RAB38//RAB22A//LHFPL2//RAP1B//ITGAV//SNAP25//SLC17A7//RAP2C//ENPP4//RTN1//ELOVL4//STEAP2//CASD1//PSD//EPHA4//MTX3//PMAIP1//MIEF1//MOAP1//SNN//MICU3//SLC25A32//SULF1//FAM91A1//C16ORF70//LIMA1//SYCP3//BRMS1L//SESN1//NFIB//PAK6//BTBD10//CDK19//HIPK3//FLI1//CNOT7//BHLHE40//COX7A2//KRT27//DPYSL2//WDR47//RASSF3//MID1IP1//CCT6A//ARHGAP6//TWF1//ACTC1//TSC1//MAPRE3//APPL1//ARHGAP1//SNX2//ZFYVE21//SLC32A1//SLC17A6//CHMP3//LRP6//PLEKHB2//FAM118B//CRY2//DENND1B//MEOX2//MPP1//SLC2A4RG//COX8C//IMMP2L//CDC37L1//IGF1//NHLRC2//TGFB2//VEGFB//NEFM//NEFH//ZNF804A//DR1//EPC2//ANP32E// |
| GO:0044422 | organelle part | Cellular component | 556 | 9321 | 970 | 18698 | 1.14983547847284 | 1.00424296322809e-06 | 6.16174789580664e-05 | 5.998161202705 | 0.57319587628866 | RDH10//EPS8//RHOC//MPP1//CDC14A//PIKFYVE//RAB21//APPL1//GRB2//NCK1//SLC2A4//TBC1D20//NUP35//DUSP2//WDFY3//CNEP1R1//NSMF//GATA6//RANBP6//GNAQ//IPO5//LBR//DNAJB12//CCND2//ATXN3//TMEM135//BNIP3//ITM2B//RAB38//PLEKHF2//DDX6//GNB5//ATG16L1//KIF5A//RAB28//TMEM17//C5ORF30//TOPORS//NEDD1//DZIP1//PKD2//CEP41//DRD1//EPS15//SQSTM1//DYNLL2//PRKACB//PRKAR2B//CEP128//REV3L//MMP2//ACTC1//TMOD1//CALM2//CFL2//FHL2//PALLD//PPP1R12A//SCN1A//NPNT//RPL15//KAT2B//AHNAK//FXR1//CTSA//GRIA1//IQGAP2//RAB9B//ORMDL3//ZNRF2//FLRT1//CLIC4//SNX5//GPRC5C//CLIP1//YWHAG//FZD6//AP1S2//ULK2//WNT7A//WASL//MICU3//STAT3//SLC25A32//COX7A2L//CNIH1//ADAM10//STX6//RTN3//SEC23A//FUT9//SERINC3//CLCN5//RAB12//HS3ST5//ST6GALNAC3//GJA1//ARFIP1//RAB30//GOLGA3//GOLGA4//ST8SIA5//ST6GALNAC6//MIA3//RND3//MAN1A1//MAN2A1//NSF//ST8SIA3//NDFIP2//TMED9//PPP6C//SYBU//CHST7//PMEPA1//MARCH4//RHEB//XYLT2//ACBD3//ST3GAL3//PLEKHA3//TMF1//APOO//B3GNT5//GNPTG//ST6GAL2//CHST1//B3GALT2//ST3GAL5//MCFD2//DSEL//MMGT1//HS2ST1//EDEM1//HS3ST3A1//BCL2L11//EPHA4//ACSL1//SLC44A1//MTX3//NME1//PMAIP1//MIEF1//MOAP1//SNN//SBF2//CLCN6//VPS37A//STEAP2//ARHGAP1//LDLR//MYO1B//RAB23//RAB22A//KIF13A//SNX2//TSG101//ZFYVE21//LAMTOR3//ASPH//ERGIC2//IER3IP1//CASD1//SLC32A1//SLC17A7//SLC17A6//PSD//PGRMC2//UBXN4//RNF180//NUP43//UBE2I//CHCHD10//NLN//THOP1//IDH1//IMPDH1//SERPINI1//SULF1//FAM91A1//SNAP25//C16ORF70//DPY30//RAB3GAP1//CETN1//TMOD3//FASTK//PABPC5//BCAT2//CRTAP//CKAP4//COL1A2//DNAJB9//FAM20C//ANO8//WFS1//KDELC1//OGN//HS3ST1//GNS//MAPK1//FMR1//SYCP3//PPP2CA//CENPQ//RAD21//DYNC1LI2//FBXO28//MTBP//DCTN6//CENPO//PTGES3//NABP1//ESCO2//BAHD1//MAF//RBL2//H2AFZ//GLYR1//KLF2//CEBPB//POLR3G//E2F1//ESR1//IRF1//AR//JUN//MEF2D//POU4F1//SMARCD2//RUNX3//KLF4//HAND2//USP3//RNF2//BRMS1L//TRERF1//ZNF217//TBL1XR1//ZBTB18//HMGA2//KLF6//E2F5//SESN1//NFIB//CC2D1A//CDCA7L//PAK6//BTBD10//AGFG1//CDK6//GDF11//CDK8//CDKN1A//IRF9//TACC2//KHDRBS3//MAP3K2//ZMYND11//TCERG1//COPS8//DNAJB4//FZD10//DUSP10//EGLN3//KLF12//ACOT7//TADA1//PTPDC1//CRKL//PDIK1L//PPP4R2//RNF38//SH3D19//CREBRF//GADD45A//DCP2//DNMT3B//DUSP5//E2F3//S1PR1//ZNF367//EGR2//ELAVL2//CC2D1B//ELK3//ETS1//EZH1//FOXC1//KDM2A//KLHDC10//FLII//CIC//USP24//ZFPM2//MORC3//NR5A2//HBP1//MKNK2//ATAD2//GTF2E1//GRHL1//NRBF2//NRG1//HIVEP2//HOXA3//HOXD1//HSBP1//JARID2//ZBTB34//MBNL1//MDM4//NR3C2//MYB//NEUROD1//NFIA//NFE2//NPAS2//NPAT//NR4A2//DCAF8//ACOX1//RLIM//KLF3//CTDSPL2//PIAS4//ARID4B//BRWD1//PPARG//BNC2//ESRP1//UBE2W//FBXW7//PPP3R1//ELMOD1//PRKAA1//INTS8//NPLOC4//BDP1//DCAF6//FEM1C//PTEN//PTHLH//REXO1//TSHZ3//USP28//ZBTB4//MIER1//PTPN4//RRAGD//RARB//ARID4A//RBBP7//RBBP8//PLEKHA1//REST//RGS3//RNF4//ATXN1//PRDM16//SRSF2//SOX17//ZMAT3//CPEB1//SHOX//SKP1//UBE2Z//BMPR2//SNAPC1//SOX4//STAT4//ZEB1//TEAD1//TFAP2A//TFAP2C//TGIF1//THRA//LDLRAD4//ZNF131//DEK//BAG6//SNIP1//NR4A3//ADAM12//FOSL1//SGPP1//TCF7L1//ARID5B//PCGF5//LCOR//MASTL//NUDCD1//USP45//RTCA//CBFB//CDC23//URI1//CCNC//RPS6KA4//BTAF1//CCNE2//ACBD5//WDR20//RPS6KA5//TRIP10//HAND1//KIF23//CLOCK//KMT2B//GIT2//RBM25//AKAP11//PPM1E//SERTAD3//MDFIC//MNX1//YPEL2//NF2//NOVA1//CMPK1//PIK3CB//SLC14A1//ZNF655//PER2//HAUS8//TGFB1I1//CDK19//HIPK3//FLI1//CNOT7//BHLHE40//COX7A2//FRY//LATS2//MZT1//KRT27//LIMA1//MARCKS//MDM1//SLAIN2//CAPRIN2//CEP97//CAMK2N2//LCA5//YTHDF2//PXK//C7ORF43//EVI5//FAM110B//MAP7D1//PTP4A1//KBTBD8//DCX//DPYSL2//REEP3//WDR47//RASSF3//MID1IP1//CCT6A//RANBP9//LRP8//ARHGAP6//TWF1//TSC1//MTPN//CALD1//DLC1//CD2AP//MAPRE3//HECW2//GOLGA8A//TMEM87A//SAR1B//UXS1//CALN1//USP6NL//TRAPPC8//PIGA//ARL6IP1//DERL2//GJC1//CDS1//OSBPL5//PPM1L//VKORC1L1//SPTSSA//SMIM14//SLC35D1//LCLAT1//SERP1//SHISA2//JKAMP//TMX3//UGT1A10//UGT1A8//UGT1A7//UGT1A6//UGT1A5//UGT1A9//UGT1A4//UGT1A1//UGT1A3//KLHL14//STIM2//MSMO1//SCD5//ELOVL7//GSG1//RTN1//ELOVL4//EDN1//SYT6//MAP3K5//NECAP1//CALCOCO2//C6ORF120//TTR//PPT2//PEX5L//NAPEPLD//CHRM2//LHFPL2//LRP6//ZFYVE9//MMD//MCOLN2//CHMP3//LAPTM4A//RAP1B//ITGAV//PLEKHB2//RAP2C//ENPP4//DR1//EPC2//NEFM//NEFH//OSTM1//SPHK2//GPR137B//VLDLR//CYBRD1//ZNF804A//FAM118B//CRY2//DENND1B//MEOX2//SLC2A4RG//CDC37L1//IGF1//NHLRC2//TGFB2//VEGFB//ANP32E//COX8C//IMMP2L// |
| GO:0031981 | nuclear lumen | Cellular component | 272 | 4071 | 970 | 18698 | 1.28792692593071 | 1.38126507971765e-06 | 7.91004468984974e-05 | 5.8597229676116 | 0.280412371134021 | ZBTB18//JUN//HMGA2//CDK6//TOPORS//GDF11//STX6//CDK8//CDKN1A//IRF9//CEBPB//TACC2//POLR3G//KHDRBS3//PTGES3//MAP3K2//ZMYND11//TCERG1//COPS8//DNAJB4//FZD10//DUSP10//EGLN3//KLF12//ACOT7//TADA1//NUP35//VPS37A//PTPDC1//CRKL//PDIK1L//PPP4R2//RNF38//SH3D19//CREBRF//ESCO2//GADD45A//DCP2//DNMT3B//DUSP5//E2F1//E2F3//E2F5//S1PR1//ZNF367//EGR2//ELAVL2//CC2D1B//ELK3//ESR1//ETS1//EZH1//FHL2//DZIP1//BAHD1//FOXC1//KDM2A//KLHDC10//FLII//CIC//FMR1//USP24//ZFPM2//SLC44A1//MORC3//NR5A2//ST6GALNAC3//NSMF//GATA6//HBP1//GJA1//GOLGA3//MKNK2//GRB2//ATAD2//GTF2E1//GRHL1//NRBF2//NRG1//HIVEP2//HOXA3//HOXD1//HSBP1//IRF1//AR//JARID2//ZBTB34//MBNL1//MDM4//MEF2D//ATXN3//NR3C2//MYB//PPP1R12A//NEUROD1//NFIA//NFE2//NPAS2//NPAT//NR4A2//DCAF8//ACOX1//RLIM//KLF3//CTDSPL2//PIAS4//ARID4B//BRWD1//POU4F1//PPARG//CTSA//BNC2//ESRP1//CENPQ//UBE2W//FBXW7//PPP3R1//ELMOD1//PRKAA1//INTS8//NPLOC4//PRKACB//TRERF1//BDP1//DCAF6//MAPK1//FEM1C//PTEN//PTHLH//REXO1//TSHZ3//USP28//ZBTB4//MIER1//PTPN4//RRAGD//RAD21//RARB//ARID4A//RBBP7//RBBP8//PLEKHA1//RBL2//REST//REV3L//RGS3//RNF2//RNF4//ATXN1//SCN1A//PRDM16//SRSF2//SOX17//ZMAT3//CPEB1//SHOX//NABP1//SKP1//UBE2Z//BMPR2//SMARCD2//SNAPC1//BNIP3//SOX4//STAT3//STAT4//ZEB1//TEAD1//TFAP2A//TFAP2C//TGIF1//THRA//UBE2I//LDLRAD4//ZNF131//ZNF217//DEK//BAG6//CENPO//TBL1XR1//SNIP1//NR4A3//ADAM12//FOSL1//SGPP1//TCF7L1//ARID5B//PCGF5//LCOR//GLYR1//DPY30//MASTL//NUDCD1//USP45//CDC14A//RTCA//RUNX3//CBFB//CDC23//URI1//KAT2B//SQSTM1//CCNC//CCND2//RPS6KA4//BTAF1//CCNE2//ACBD5//WDR20//RPS6KA5//KLF4//TRIP10//HAND1//KIF23//CLOCK//KMT2B//GIT2//USP3//AKAP11//KLF6//DDX6//PPM1E//WDFY3//SERTAD3//MDFIC//MNX1//IPO5//YPEL2//DNAJB9//NF2//NOVA1//ERGIC2//CMPK1//PIK3CB//CDCA7L//SLC14A1//TSG101//ZNF655//FXR1//PER2//COX7A2L//RANBP6//CFL2//CLIC4//TGFB1I1//KLF2//H2AFZ//HAND2//SYCP3//ANP32E//APPL1//BRMS1L//DR1//FAM118B//CDK19//HIPK3//FLI1//CNOT7//BHLHE40//CKAP4//CRY2//DENND1B//MEOX2//MPP1//SLC2A4RG//RBM25//EPC2//SESN1//NFIB//CC2D1A//PAK6//BTBD10// |
| GO:0031984 | organelle subcompartment | Cellular component | 128 | 1655 | 970 | 18698 | 1.4908549537484 | 2.44041000149271e-06 | 0.00013101951195514 | 5.61253720381398 | 0.131958762886598 | SULF1//RAB30//NSF//MARCH4//STX6//FAM91A1//RAB12//RAB21//RAB38//GOLGA4//SNAP25//C16ORF70//DPY30//RAB3GAP1//GOLGA8A//MAN2A1//ASPH//ARFIP1//MYO1B//KIF13A//CALN1//AP1S2//USP6NL//FUT9//TMEM87A//GOLGA3//SAR1B//ST3GAL3//UXS1//ST6GAL2//CNIH1//ADAM10//RTN3//SEC23A//SERINC3//CLCN5//PIKFYVE//HS3ST5//ST6GALNAC3//GJA1//GRIA1//ST8SIA5//ST6GALNAC6//MIA3//RND3//MAN1A1//ST8SIA3//NDFIP2//TMED9//PPP6C//SYBU//CHST7//PMEPA1//RHEB//XYLT2//ACBD3//PLEKHA3//TMF1//APOO//B3GNT5//GNPTG//CHST1//B3GALT2//ST3GAL5//MCFD2//DSEL//MMGT1//ITM2B//HS2ST1//EDEM1//HS3ST3A1//GJC1//CDS1//CKAP4//OSBPL5//TBC1D20//PPM1L//VKORC1L1//RDH10//SPTSSA//DRD1//SMIM14//ACSL1//REEP3//SLC35D1//UBXN4//ARL6IP1//FLRT1//LCLAT1//SERP1//RNF180//SHISA2//DNAJB9//ATXN3//NR3C2//DERL2//IER3IP1//ERGIC2//JKAMP//PIGA//PKD2//TMX3//UGT1A10//UGT1A8//UGT1A7//UGT1A6//UGT1A5//UGT1A9//UGT1A4//UGT1A1//UGT1A3//KLHL14//STIM2//MSMO1//WFS1//SCD5//ELOVL7//SGPP1//GSG1//NPLOC4//MAP3K5//STEAP2//CASD1//ORMDL3//LBR//DNAJB12//RTN1//ELOVL4// |
| GO:0000139 | Golgi membrane | Cellular component | 67 | 729 | 970 | 18698 | 1.77162049410999 | 3.80201284140565e-06 | 0.000186197557763015 | 5.41998642062623 | 0.0690721649484536 | FUT9//RAB21//GOLGA8A//TMEM87A//GOLGA3//SAR1B//ST3GAL3//UXS1//ST6GAL2//STX6//ARFIP1//MYO1B//KIF13A//CALN1//AP1S2//USP6NL//TBC1D20//STEAP2//IER3IP1//CASD1//CNIH1//ADAM10//RTN3//SEC23A//SERINC3//CLCN5//PIKFYVE//RAB12//HS3ST5//ST6GALNAC3//GJA1//RAB30//GOLGA4//GRIA1//ST8SIA5//ST6GALNAC6//MIA3//RND3//MAN1A1//MAN2A1//NSF//ST8SIA3//NDFIP2//TMED9//PPP6C//SYBU//CHST7//PMEPA1//MARCH4//RHEB//XYLT2//ACBD3//PLEKHA3//TMF1//APOO//B3GNT5//GNPTG//CHST1//B3GALT2//ST3GAL5//MCFD2//DSEL//MMGT1//ITM2B//HS2ST1//EDEM1//HS3ST3A1// |
| GO:0005654 | nucleoplasm | Cellular component | 235 | 3472 | 970 | 18698 | 1.30470271746876 | 3.9016950404357e-06 | 0.000186197557763015 | 5.40874667841452 | 0.242268041237113 | TRERF1//ZNF217//TBL1XR1//TADA1//KAT2B//URI1//POLR3G//GTF2E1//DR1//BAHD1//FMR1//NPAT//FAM118B//APPL1//RBBP7//CDK8//CDK19//CCNC//HIPK3//CDKN1A//CREBRF//FLI1//MKNK2//CNOT7//ESRP1//REXO1//USP28//ZBTB4//RNF2//SCN1A//BHLHE40//CDC14A//TOPORS//WDFY3//MORC3//NFE2//PIAS4//PTEN//RNF4//UBE2I//SQSTM1//CKAP4//DUSP10//CRY2//DENND1B//GADD45A//HBP1//AR//MEOX2//MPP1//NR4A2//PRKAA1//SLC2A4RG//RBM25//SRSF2//GLYR1//INTS8//EPC2//JARID2//DPY30//KMT2B//EZH1//ATXN1//BRMS1L//CDK6//GDF11//STX6//IRF9//CEBPB//TACC2//KHDRBS3//PTGES3//MAP3K2//ZMYND11//TCERG1//COPS8//DNAJB4//FZD10//EGLN3//KLF12//ACOT7//NUP35//VPS37A//PTPDC1//CRKL//PDIK1L//PPP4R2//RNF38//SH3D19//ESCO2//DCP2//DNMT3B//DUSP5//E2F1//E2F3//E2F5//S1PR1//ZNF367//EGR2//ELAVL2//CC2D1B//ELK3//ESR1//ETS1//FHL2//DZIP1//FOXC1//KDM2A//KLHDC10//FLII//CIC//USP24//ZFPM2//SLC44A1//NR5A2//ST6GALNAC3//NSMF//GATA6//GJA1//GOLGA3//GRB2//ATAD2//GRHL1//NRBF2//NRG1//HIVEP2//HOXA3//HOXD1//HSBP1//IRF1//JUN//ZBTB34//MBNL1//MDM4//MEF2D//ATXN3//NR3C2//MYB//PPP1R12A//NEUROD1//NFIA//NPAS2//DCAF8//ACOX1//RLIM//KLF3//CTDSPL2//ARID4B//BRWD1//POU4F1//PPARG//CTSA//BNC2//CENPQ//UBE2W//FBXW7//PPP3R1//ELMOD1//NPLOC4//PRKACB//BDP1//DCAF6//MAPK1//FEM1C//PTHLH//TSHZ3//MIER1//PTPN4//RRAGD//RAD21//RARB//ARID4A//RBBP8//PLEKHA1//RBL2//REST//REV3L//RGS3//PRDM16//SOX17//ZMAT3//CPEB1//SHOX//NABP1//SKP1//UBE2Z//BMPR2//SMARCD2//SNAPC1//BNIP3//SOX4//STAT3//STAT4//ZEB1//TEAD1//TFAP2A//TFAP2C//TGIF1//THRA//LDLRAD4//ZNF131//DEK//BAG6//CENPO//SNIP1//NR4A3//ADAM12//FOSL1//HMGA2//SGPP1//TCF7L1//ARID5B//PCGF5//LCOR//MASTL//NUDCD1//USP45//RTCA//RUNX3//CBFB//CDC23//CCND2//RPS6KA4//BTAF1//CCNE2//ACBD5//WDR20//RPS6KA5//KLF4//TRIP10//HAND1//KIF23//CLOCK//GIT2//USP3// |
| GO:0098791 | Golgi subcompartment | Cellular component | 73 | 819 | 970 | 18698 | 1.71815515526856 | 4.2023712416247e-06 | 0.00018999141560819 | 5.37650558423199 | 0.0752577319587629 | CNIH1//ADAM10//STX6//RTN3//SEC23A//FUT9//SERINC3//CLCN5//PIKFYVE//RAB12//HS3ST5//ST6GALNAC3//GJA1//ARFIP1//RAB30//GOLGA3//GOLGA4//GRIA1//ST8SIA5//ST6GALNAC6//MIA3//RND3//MAN1A1//MAN2A1//NSF//ST8SIA3//NDFIP2//TMED9//PPP6C//SYBU//CHST7//PMEPA1//MARCH4//RHEB//XYLT2//ACBD3//ST3GAL3//PLEKHA3//TMF1//APOO//B3GNT5//GNPTG//ST6GAL2//CHST1//B3GALT2//ST3GAL5//AP1S2//MCFD2//DSEL//MMGT1//ITM2B//HS2ST1//EDEM1//HS3ST3A1//SULF1//FAM91A1//RAB21//RAB38//SNAP25//C16ORF70//DPY30//GOLGA8A//MYO1B//KIF13A//CALN1//USP6NL//TMEM87A//SAR1B//UXS1//TBC1D20//STEAP2//IER3IP1//CASD1// |
| GO:0005794 | Golgi apparatus | Cellular component | 117 | 1501 | 970 | 18698 | 1.50254881625308 | 4.83317969906006e-06 | 0.00020758506807463 | 5.31576705736281 | 0.120618556701031 | GOLGA8A//CNIH1//ADAM10//STX6//RTN3//SEC23A//FUT9//SERINC3//CLCN5//PIKFYVE//RAB12//HS3ST5//ST6GALNAC3//GJA1//ARFIP1//RAB30//GOLGA3//GOLGA4//GRIA1//ST8SIA5//ST6GALNAC6//MIA3//RND3//MAN1A1//MAN2A1//NSF//ST8SIA3//NDFIP2//TMED9//PPP6C//SYBU//CHST7//PMEPA1//MARCH4//RHEB//XYLT2//ACBD3//ST3GAL3//PLEKHA3//TMF1//APOO//B3GNT5//GNPTG//ST6GAL2//CHST1//B3GALT2//ST3GAL5//AP1S2//MCFD2//DSEL//MMGT1//ITM2B//HS2ST1//EDEM1//HS3ST3A1//SULF1//OGN//WNT7A//HS3ST1//FAM91A1//RAB21//RAB38//SNAP25//C16ORF70//DPY30//TBC1D20//STEAP2//IER3IP1//CASD1//TMEM87A//SAR1B//UXS1//MYO1B//KIF13A//CALN1//USP6NL//TRAPPC8//TRIM3//ADCY3//STMN2//DUSP10//SCRG1//FGD4//UBXN2B//FGD5//ESCO2//EPHA4//ESR1//RAB3GAP1//MMD//GRB2//ZDHHC1//GRHL1//ITGA5//KDR//FIBIN//LDLR//LRP6//ACO1//MINK1//ERGIC2//C11ORF24//MAPK1//FAM20C//PTHLH//TFAP2A//EVI5//FYCO1//RNF128//KBTBD8//GLYR1//SGCE//CCDC126//PKDCC//TRIP10//CXCL14//LAPTM4A// |
| GO:0044428 | nuclear part | Cellular component | 287 | 4435 | 970 | 18698 | 1.24741710154697 | 9.49613934337927e-06 | 0.000388437318855371 | 5.02245292130529 | 0.295876288659794 | ZBTB18//JUN//HMGA2//PGRMC2//NUP35//WDFY3//UBXN4//CNEP1R1//NSMF//RNF180//NUP43//LBR//CLIP1//BNIP3//UBE2I//AGFG1//IPO5//CDK6//TOPORS//GDF11//STX6//CDK8//CDKN1A//IRF9//CEBPB//TACC2//POLR3G//KHDRBS3//PTGES3//MAP3K2//ZMYND11//TCERG1//COPS8//DNAJB4//FZD10//DUSP10//EGLN3//KLF12//ACOT7//TADA1//VPS37A//PTPDC1//CRKL//PDIK1L//PPP4R2//RNF38//SH3D19//CREBRF//ESCO2//GADD45A//DCP2//DNMT3B//DUSP5//E2F1//E2F3//E2F5//S1PR1//ZNF367//EGR2//ELAVL2//CC2D1B//ELK3//ESR1//ETS1//EZH1//FHL2//DZIP1//BAHD1//FOXC1//KDM2A//KLHDC10//FLII//CIC//FMR1//USP24//ZFPM2//SLC44A1//MORC3//NR5A2//ST6GALNAC3//GATA6//HBP1//GJA1//GOLGA3//MKNK2//GRB2//ATAD2//GTF2E1//GRHL1//NRBF2//NRG1//HIVEP2//HOXA3//HOXD1//HSBP1//IRF1//AR//JARID2//ZBTB34//MBNL1//MDM4//MEF2D//ATXN3//NR3C2//MYB//PPP1R12A//NEUROD1//NFIA//NFE2//NPAS2//NPAT//NR4A2//DCAF8//ACOX1//RLIM//KLF3//CTDSPL2//PIAS4//ARID4B//BRWD1//POU4F1//PPARG//CTSA//BNC2//ESRP1//CENPQ//UBE2W//FBXW7//PPP3R1//ELMOD1//PRKAA1//INTS8//NPLOC4//PRKACB//TRERF1//BDP1//DCAF6//MAPK1//FEM1C//PTEN//PTHLH//REXO1//TSHZ3//USP28//ZBTB4//MIER1//PTPN4//RRAGD//RAD21//RARB//ARID4A//RBBP7//RBBP8//PLEKHA1//RBL2//REST//REV3L//RGS3//RNF2//RNF4//ATXN1//SCN1A//PRDM16//SRSF2//SOX17//ZMAT3//CPEB1//SHOX//NABP1//SKP1//UBE2Z//BMPR2//SMARCD2//SNAPC1//SOX4//STAT3//STAT4//ZEB1//TEAD1//TFAP2A//TFAP2C//TGIF1//THRA//LDLRAD4//ZNF131//ZNF217//DEK//BAG6//CENPO//TBL1XR1//SNIP1//NR4A3//ADAM12//FOSL1//SGPP1//TCF7L1//ARID5B//PCGF5//LCOR//GLYR1//DPY30//MASTL//NUDCD1//USP45//CDC14A//RTCA//RUNX3//CBFB//CDC23//URI1//KAT2B//SQSTM1//CCNC//CCND2//RPS6KA4//BTAF1//CCNE2//ACBD5//WDR20//RPS6KA5//KLF4//TRIP10//HAND1//KIF23//CLOCK//KMT2B//GIT2//USP3//RBM25//RHEB//AKAP11//KLF6//DDX6//PPM1E//SERTAD3//MDFIC//MNX1//YPEL2//DNAJB9//NF2//NOVA1//ERGIC2//CMPK1//PIK3CB//CDCA7L//SLC14A1//TSG101//ZNF655//FXR1//PER2//COX7A2L//HAUS8//TMOD1//CFL2//CLIC4//TGFB1I1//TBC1D20//DUSP2//RANBP6//GNAQ//DNAJB12//CDK19//HIPK3//FLI1//CNOT7//BHLHE40//SESN1//NFIB//CC2D1A//PAK6//BTBD10//KLF2//H2AFZ//HAND2//SYCP3//BRMS1L//APPL1//DR1//FAM118B//CKAP4//CRY2//DENND1B//MEOX2//MPP1//SLC2A4RG//ANP32E//EPC2// |
| GO:0098588 | bounding membrane of organelle | Cellular component | 148 | 2040 | 970 | 18698 | 1.39847584394583 | 1.25893311587872e-05 | 0.0004915561575181 | 4.89999734231183 | 0.152577319587629 | CNIH1//ADAM10//STX6//RTN3//SEC23A//FUT9//SERINC3//CLCN5//PIKFYVE//RAB12//HS3ST5//ST6GALNAC3//GJA1//ARFIP1//RAB30//GOLGA3//GOLGA4//GRIA1//ST8SIA5//ST6GALNAC6//MIA3//RND3//MAN1A1//MAN2A1//NSF//ST8SIA3//NDFIP2//TMED9//PPP6C//SYBU//CHST7//PMEPA1//MARCH4//RHEB//XYLT2//ACBD3//ST3GAL3//PLEKHA3//TMF1//APOO//B3GNT5//GNPTG//ST6GAL2//CHST1//B3GALT2//ST3GAL5//AP1S2//MCFD2//DSEL//MMGT1//ITM2B//HS2ST1//EDEM1//HS3ST3A1//PLEKHF2//SBF2//IQGAP2//RAB9B//ORMDL3//CLCN6//VPS37A//ZNRF2//APPL1//STEAP2//ARHGAP1//LDLR//MYO1B//RAB23//RAB22A//KIF13A//SNX2//TSG101//ZFYVE21//LAMTOR3//WNT7A//WASL//ASPH//TBC1D20//ERGIC2//DRD1//TMEM17//EPS15//PKD2//RAB21//GOLGA8A//TMEM87A//SAR1B//UXS1//CALN1//USP6NL//CALCOCO2//ATG16L1//SLC17A7//LHFPL2//TMX3//CKAP4//RAP1B//ITGAV//SNAP25//RAP2C//ENPP4//EPHA4//SNX5//LRP6//LDLRAD4//ZFYVE9//MMD//MCOLN2//CHMP3//LAPTM4A//PLEKHB2//CHRM2//RAB38//ACSL1//ACOX1//PEX5L//TMEM135//ACBD5//BCL2L11//SLC44A1//MTX3//NME1//PMAIP1//MIEF1//MOAP1//BNIP3//SNN//NAPEPLD//GNAQ//OSTM1//SPHK2//GPR137B//VLDLR//AHNAK//CYBRD1//SLC32A1//SYT6//SLC17A6//C16ORF70//WDFY3//CTSA//RRAGD//NECAP1//DERL2//PSD//IER3IP1//CASD1//ARL6IP1// |
| GO:0120025 | plasma membrane bounded cell projection | Cellular component | 143 | 1980 | 970 | 18698 | 1.39217640320733 | 2.27121868757321e-05 | 0.000848250805489299 | 4.64374104709521 | 0.147422680412371 | CDK6//KLHL2//FGD4//FGD5//PALLD//CD2AP//SNX5//ITGA5//RAB22A//CLIP1//MSN//ADCY3//DYNLL2//LCA5//PKD2//CEP41//STMN2//LYPD6//CPEB2//SLC32A1//DCX//EPHA2//EPHA4//EPHA5//EPHA7//CPEB3//FMR1//NSMF//RGS17//GLRA1//GLRB//GPM6A//RHOC//NF2//POU4F1//SLC17A6//PTEN//KLHL14//KIF13A//CPEB1//BCL11B//SLC6A1//SNAP25//CPEB4//FOSL1//IQGAP2//WASF3//STX2//ENAH//SNX2//ACTC1//TSC1//WASL//MAPK1//DRD1//IQCJ-SCHIP1//MTPN//WDFY3//LPAR3//CADM2//SACS//TNFRSF21//KCNA4//NEFM//NEFH//NTF3//MINK1//MAP2K4//TGFB2//LRP8//FXR1//FEZ2//TRIM3//CHRM2//CTNND2//DMWD//ZDHHC5//NPTN//GNAQ//GRIA1//NRG1//P2RY1//BMPR2//BNIP3//STRN//WFS1//C16ORF70//TMEM185A//URI1//EPS8//MPP1//CDC14A//GJB2//CLIC4//ITGA6//MYO1B//TWF1//CNN3//LPAR1//PRKAR2B//PSD//ZNF804A//NFIB//SLC17A7//RNF38//DLC1//ITGAV//NME1//ARHGEF4//PLEK//SNTG1//PLEKHA1//SCRG1//FLRT1//SGCE//KIF5A//NSF//SLC12A5//DDX6//SQSTM1//GNB5//TOPORS//CETN1//ATG16L1//OTX2//TSHZ3//SLC6A14//CYBRD1//SCN1A//SPOCK1//RAB28//TMEM17//C5ORF30//NEDD1//DZIP1//NAPEPLD//STX6//PFN2//EPS15//PRKACB//CEP128//RAB21//SYBU// |
| GO:0012505 | endomembrane system | Cellular component | 283 | 4421 | 970 | 18698 | 1.2339266434566 | 2.69933426009806e-05 | 0.000966136720593431 | 4.56874333318055 | 0.291752577319588 | PGRMC2//NUP35//WDFY3//UBXN4//CNEP1R1//NSMF//RNF180//NUP43//LBR//CLIP1//BNIP3//UBE2I//STMN2//CLCN5//CRKL//DGKH//S1PR1//LPAR1//RAB12//RAB21//STEAP2//SNX5//GRB2//JAK1//KDR//RUFY2//ANKRD50//ARRDC3//SNX2//TSG101//ARRDC4//RTN3//CDS1//CRTAP//CKAP4//COL1A2//TBC1D20//UBXN2B//FGD5//VKORC1L1//TMEM64//SMIM14//EPHA4//PDZD2//SULF1//RAB38//LCLAT1//SERP1//GLRA1//ZDHHC1//ITGA5//FIBIN//SHISA2//LAMA3//LRP6//FAM19A1//MAN1A1//DNAJB9//ASPH//NCK1//ACO1//DERL2//IER3IP1//PKD2//TMX3//UGT1A10//UGT1A8//UGT1A7//UGT1A6//UGT1A9//NDFIP2//UGT1A4//UGT1A1//UGT1A3//TMED9//CTSA//DNAJB12//DUS2//TMEM100//NPLOC4//RGMA//PHTF2//KLHL14//STIM2//RTN1//MSMO1//HSPA13//ELOVL4//TMF1//WFS1//PTP4A1//RNF128//PLEKHF2//ELOVL7//CPEB4//CAMLG//B3GNT5//PPP1R15B//B3GALT2//SQSTM1//ORMDL3//EDEM1//ADAM10//STX6//TRIM3//FUT9//ADCY3//DUSP10//SCRG1//FGD4//ESCO2//ESR1//RAB3GAP1//GOLGA8A//MMD//GJA1//GOLGA3//GOLGA4//GRHL1//LDLR//MINK1//ERGIC2//C11ORF24//MAPK1//FAM20C//PTHLH//ACBD3//PLEKHA3//TFAP2A//EVI5//FYCO1//KBTBD8//GNPTG//GLYR1//DPY30//AP1S2//SGCE//CCDC126//PKDCC//TRIP10//MMGT1//ITM2B//CXCL14//LAPTM4A//EDN1//FCHO2//EPS15//NECAP1//EPN1//LRP12//SLC2A4//VLDLR//ADRB1//APPL1//MYO1B//NF2//RAB22A//ZFYVE9//DYNC1LI2//CHMP3//TBC1D12//GRIA1//TNF//TBC1D17//ARHGAP1//EPHA5//RASD1//STX2//NTF3//SNAP25//IGF1//AGFG1//IPO5//DUSP2//GATA6//RANBP6//GNAQ//CCND2//PIKFYVE//PMEPA1//LDLRAD4//VPS37A//MCOLN2//IQGAP2//RAB9B//IDH1//IMPDH1//SERPINI1//SLC32A1//SYT6//SLC17A7//SLC17A6//C16ORF70//LHFPL2//NHLRC2//TGFB2//VEGFB//GJC1//CNIH1//SEC23A//OSBPL5//PPM1L//RDH10//SPTSSA//DRD1//ACSL1//REEP3//SLC35D1//ARL6IP1//FLRT1//MIA3//ATXN3//NR3C2//SAR1B//JKAMP//PIGA//UGT1A5//RHEB//XYLT2//APOO//SCD5//SGPP1//GSG1//MCFD2//ITGAV//LAMTOR3//RAP1B//GNS//C6ORF120//TTR//CDC37L1//PLEKHB2//RAP2C//ENPP4//CLCN6//ZNRF2//RAB23//KIF13A//ZFYVE21//RRAGD//PSD//ANO8//WNT7A//KDELC1//MAP3K5//SERINC3//HS3ST5//ST6GALNAC3//ARFIP1//RAB30//ST8SIA5//ST6GALNAC6//RND3//MAN2A1//NSF//ST8SIA3//PPP6C//SYBU//CHST7//MARCH4//ST3GAL3//ST6GAL2//CHST1//ST3GAL5//DSEL//HS2ST1//HS3ST3A1//OGN//HS3ST1//FAM91A1//CASD1//TMEM87A//UXS1//CALN1//USP6NL//TRAPPC8//BCL2L11//CDC42EP3//DOCK9//CD274//DNAJC27//PDCD1LG2//RAB28// |
| GO:0044431 | Golgi apparatus part | Cellular component | 77 | 935 | 970 | 18698 | 1.58745906610067 | 3.78076118476753e-05 | 0.00129906954308612 | 4.4224207543786 | 0.0793814432989691 | CNIH1//ADAM10//STX6//RTN3//SEC23A//FUT9//SERINC3//CLCN5//PIKFYVE//RAB12//HS3ST5//ST6GALNAC3//GJA1//ARFIP1//RAB30//GOLGA3//GOLGA4//GRIA1//ST8SIA5//ST6GALNAC6//MIA3//RND3//MAN1A1//MAN2A1//NSF//ST8SIA3//NDFIP2//TMED9//PPP6C//SYBU//CHST7//PMEPA1//MARCH4//RHEB//XYLT2//ACBD3//ST3GAL3//PLEKHA3//TMF1//APOO//B3GNT5//GNPTG//ST6GAL2//CHST1//B3GALT2//ST3GAL5//AP1S2//MCFD2//DSEL//MMGT1//ITM2B//HS2ST1//EDEM1//HS3ST3A1//OGN//WNT7A//HS3ST1//GOLGA8A//TRAPPC8//RAB21//TMEM87A//SAR1B//UXS1//MYO1B//KIF13A//CALN1//USP6NL//TBC1D20//STEAP2//IER3IP1//CASD1//SULF1//FAM91A1//RAB38//SNAP25//C16ORF70//DPY30// |
| GO:0001726 | ruffle | Cellular component | 22 | 164 | 970 | 18698 | 2.58584360070405 | 4.09149461421689e-05 | 0.00135176687446627 | 4.38811801616512 | 0.022680412371134 | DLC1//FGD5//EPHA2//EPS8//ITGA5//ITGAV//NF2//NME1//ARHGEF4//PLEK//SNTG1//PSD//TWF1//PLEKHA1//CDK6//KLHL2//FGD4//PALLD//CD2AP//SNX5//RAB22A//CLIP1// |
| GO:0042995 | cell projection | Cellular component | 145 | 2055 | 970 | 18698 | 1.36012742368375 | 6.25209107154356e-05 | 0.00198909119646516 | 4.20397470442432 | 0.149484536082474 | CDK6//KLHL2//FGD4//FGD5//PALLD//CD2AP//SNX5//ITGA5//RAB22A//CLIP1//MSN//ADCY3//DYNLL2//LCA5//PKD2//CEP41//STMN2//LYPD6//CPEB2//SLC32A1//DCX//EPHA2//EPHA4//EPHA5//EPHA7//CPEB3//FMR1//NSMF//RGS17//GLRA1//GLRB//GPM6A//RHOC//NF2//POU4F1//SLC17A6//PTEN//KLHL14//KIF13A//CPEB1//BCL11B//SLC6A1//SNAP25//CPEB4//FOSL1//IQGAP2//WASF3//STX2//ENAH//SNX2//ACTC1//TSC1//WASL//MAPK1//DRD1//IQCJ-SCHIP1//MTPN//WDFY3//LPAR3//CADM2//SACS//TNFRSF21//KCNA4//NEFM//NEFH//NTF3//MINK1//MAP2K4//TGFB2//LRP8//FXR1//FEZ2//TRIM3//CHRM2//CTNND2//DMWD//ZDHHC5//NPTN//GNAQ//GRIA1//NRG1//P2RY1//BMPR2//BNIP3//STRN//WFS1//C16ORF70//TMEM185A//URI1//EPS8//MPP1//CNN3//LPAR1//PRKAR2B//PSD//ZNF804A//NFIB//SLC17A7//CDC14A//GJB2//CLIC4//ITGA6//MYO1B//TWF1//RNF38//DLC1//ITGAV//NME1//ARHGEF4//PLEK//SNTG1//PLEKHA1//SCRG1//FLRT1//SGCE//KIF5A//NSF//SLC12A5//DDX6//SQSTM1//GNB5//TOPORS//CETN1//ATG16L1//OTX2//TSHZ3//SLC6A14//CYBRD1//SCN1A//SPOCK1//RAB28//TMEM17//C5ORF30//NEDD1//DZIP1//NAPEPLD//STX6//PFN2//EPS15//PRKACB//CEP128//RAB21//SYBU//AGFG1//TRIP10// |
| GO:0043005 | neuron projection | Cellular component | 94 | 1224 | 970 | 18698 | 1.48036857354626 | 7.72233432774745e-05 | 0.00236910185269109 | 4.11225140012808 | 0.0969072164948454 | IQCJ-SCHIP1//MTPN//EPHA4//EPHA5//WDFY3//FMR1//LPAR3//CADM2//SACS//TNFRSF21//KCNA4//NEFM//NEFH//NTF3//MINK1//MAPK1//KIF13A//MAP2K4//SLC6A1//TGFB2//LRP8//FXR1//FEZ2//TRIM3//CHRM2//SLC32A1//CTNND2//DMWD//EPHA7//CPEB3//ZDHHC5//NSMF//NPTN//GLRA1//GLRB//GNAQ//GRIA1//NRG1//P2RY1//CPEB1//BMPR2//BNIP3//STRN//WFS1//C16ORF70//CPEB4//TMEM185A//URI1//EPS8//RHOC//MPP1//SCRG1//FLRT1//CDC14A//NFIB//SLC17A7//CNN3//LPAR1//GPM6A//PRKAR2B//PSD//PTEN//ZNF804A//SGCE//KIF5A//NSF//SLC12A5//GNB5//TOPORS//CETN1//RAB21//SYBU//STMN2//PALLD//OTX2//TSHZ3//SNAP25//TSC1//SCN1A//SPOCK1//STX6//PFN2//NAPEPLD//LYPD6//CPEB2//DCX//EPHA2//RGS17//NF2//POU4F1//SLC17A6//KLHL14//BCL11B//FOSL1// |
| GO:0036477 | somatodendritic compartment | Cellular component | 64 | 766 | 970 | 18698 | 1.61055153293316 | 0.000113805147538643 | 0.00337098695640325 | 3.94383809385422 | 0.065979381443299 | STMN2//CHRM2//CNN3//LPAR1//EPHA5//EPHA7//FMR1//GLRA1//GPM6A//GRIA1//AGFG1//KIF5A//LRP6//SERPINI1//PRKAR2B//SLC12A5//KLHL14//SCN1A//BMPR2//SOS1//STRN//TGFB2//LRP8//FXR1//ZNF804A//FLRT1//PDE10A//RBFOX3//CTNND2//DMWD//EPHA4//WDFY3//NSMF//NEUROG1//TMEM100//MAPK1//MAP2K4//TRIM3//SLC32A1//CPEB3//ZDHHC5//SACS//NPTN//GLRB//GNAQ//NRG1//NTF3//P2RY1//MINK1//KIF13A//CPEB1//BNIP3//WFS1//C16ORF70//CPEB4//TMEM185A//URI1//SGCE//KCNA4//PSD//PTEN//NSF//LDLR//SNAP25// |
| GO:0005667 | transcription factor complex | Cellular component | 35 | 345 | 970 | 18698 | 1.95556551621097 | 0.000121324762354172 | 0.00340138507036434 | 3.91605055068956 | 0.0360824742268041 | SOX17//SOX4//KLF4//SNAPC1//E2F3//NR5A2//NEUROD1//PPARG//RARB//STAT3//THRA//HAND1//BDP1//GTF2E1//E2F1//JUN//CEBPB//TEAD1//E2F5//ETS1//FOXF1//FOXF2//GATA6//NHLH2//NPAS2//NR4A2//PBX3//POU3F2//TRERF1//RBL2//ZEB1//NR4A3//TCF7L1//HAND2//CLOCK// |
| GO:0097458 | neuron part | Cellular component | 112 | 1533 | 970 | 18698 | 1.40831332674293 | 0.000122750799978224 | 0.00340138507036434 | 3.91097566885879 | 0.115463917525773 | GNB5//GNAQ//STX2//GRIA1//NTF3//SNAP25//ADAM10//CNN3//EPHA4//EPS8//CPEB3//FMR1//NSMF//NPTN//NEFH//NSF//P2RY1//MINK1//PSD//CPEB1//BMPR2//BNIP3//SOS1//SPOCK1//STRN//LRP8//CPEB4//FXR1//STMN2//LYPD6//CPEB2//SLC32A1//DCX//EPHA2//EPHA5//EPHA7//RGS17//GLRA1//GLRB//GPM6A//RHOC//NF2//POU4F1//SLC17A6//PTEN//KLHL14//KIF13A//BCL11B//SLC6A1//FOSL1//SYT6//SLC17A7//C16ORF70//TOPORS//CETN1//FLRT1//LDLR//NAPEPLD//ZNRF2//CHRM2//LPAR1//AGFG1//KIF5A//LRP6//SERPINI1//PRKAR2B//SLC12A5//SCN1A//TGFB2//ZNF804A//PDE10A//RBFOX3//CTNND2//DMWD//WDFY3//NEUROG1//TMEM100//MAPK1//MAP2K4//SCRG1//PPFIA1//SQSTM1//NEFM//KCNJ10//WNT7A//FCHO2//IQCJ-SCHIP1//MTPN//LPAR3//CADM2//SACS//TNFRSF21//KCNA4//FEZ2//TRIM3//ZDHHC5//NRG1//WFS1//TMEM185A//URI1//MPP1//CDC14A//SGCE//PALLD//OTX2//TSHZ3//TSC1//STX6//PFN2//RAB21//SYBU//NFIB// |
| GO:0032587 | ruffle membrane | Cellular component | 14 | 90 | 970 | 18698 | 2.99853379152348 | 0.000211288951016302 | 0.00567178777884386 | 3.67512321301921 | 0.0144329896907217 | DLC1//FGD5//EPHA2//EPS8//ITGA5//ITGAV//NF2//NME1//ARHGEF4//PLEK//SNTG1//PSD//TWF1//PLEKHA1// |
| GO:0005768 | endosome | Cellular component | 70 | 878 | 970 | 18698 | 1.53683394781955 | 0.000223785492416583 | 0.00582520418138924 | 3.65016807137177 | 0.0721649484536082 | STX6//TRIM3//FGD5//ADRB1//RAB21//RAB38//APPL1//STEAP2//GJA1//KDR//LDLR//LRP6//MYO1B//NF2//DERL2//MAPK1//RAB22A//ARRDC3//TSG101//PTP4A1//ARRDC4//MMGT1//ZFYVE9//DYNC1LI2//CHMP3//FYCO1//RNF128//SQSTM1//TBC1D12//GRIA1//TNF//TBC1D17//ARHGAP1//SLC2A4//CLIP1//PIKFYVE//EPHA4//EPS15//SNX5//PMEPA1//SNX2//LDLRAD4//PLEKHF2//VPS37A//MMD//MCOLN2//LAPTM4A//NDFIP2//RAB12//PLEKHB2//RAP2C//CLCN5//CLCN6//ZNRF2//RAB23//KIF13A//ZFYVE21//LAMTOR3//ITM2B//RRAGD//PSD//STMN2//CRKL//DGKH//S1PR1//LPAR1//GRB2//JAK1//RUFY2//ANKRD50// |
| GO:0031252 | cell leading edge | Cellular component | 36 | 385 | 970 | 18698 | 1.8024581603963 | 0.000476826620784123 | 0.0120468843309871 | 3.32163950633637 | 0.0371134020618557 | CDK6//KLHL2//FGD4//FGD5//PALLD//CD2AP//SNX5//ITGA5//RAB22A//CLIP1//IQGAP2//WASF3//STMN2//EPHA2//STX2//NF2//PKD2//ENAH//SNX2//ACTC1//TSC1//WASL//NRG1//ITGAV//DLC1//EPS8//NME1//ARHGEF4//PLEK//SNTG1//PSD//TWF1//PLEKHA1//SGCE//GRIA1//SLK// |
| GO:0005769 | early endosome | Cellular component | 32 | 334 | 970 | 18698 | 1.84683005123773 | 0.000636541252035162 | 0.0153844968807722 | 3.19617344597623 | 0.0329896907216495 | PIKFYVE//EPHA4//EPS15//RAB21//APPL1//SNX5//GRIA1//LRP6//PMEPA1//SNX2//TSG101//LDLRAD4//PLEKHF2//MMGT1//ZFYVE9//STX6//TRIM3//FGD5//ADRB1//RAB38//STEAP2//GJA1//KDR//LDLR//MYO1B//NF2//DERL2//MAPK1//RAB22A//ARRDC3//PTP4A1//ARRDC4// |
| GO:0030425 | dendrite | Cellular component | 48 | 569 | 970 | 18698 | 1.62611925425326 | 0.000644751906528288 | 0.0153844968807722 | 3.19060736498143 | 0.0494845360824742 | SGCE//KIF5A//MAPK1//MAP2K4//CNN3//LPAR1//EPHA4//FMR1//GPM6A//GRIA1//KCNA4//PRKAR2B//PSD//PTEN//STRN//CPEB4//FXR1//ZNF804A//NSF//SLC12A5//SLC32A1//CPEB3//NSMF//TRIM3//CHRM2//CTNND2//DMWD//EPHA5//EPHA7//ZDHHC5//SACS//NPTN//GLRA1//GLRB//GNAQ//NRG1//NTF3//P2RY1//MINK1//KIF13A//CPEB1//BMPR2//BNIP3//WFS1//LRP8//C16ORF70//TMEM185A//URI1// |
| GO:0097447 | dendritic tree | Cellular component | 48 | 570 | 970 | 18698 | 1.62326641345632 | 0.000669802831031358 | 0.0155502873474577 | 3.17405302117552 | 0.0494845360824742 | TRIM3//CHRM2//SLC32A1//CTNND2//DMWD//EPHA4//EPHA5//EPHA7//CPEB3//FMR1//ZDHHC5//NSMF//SACS//NPTN//GLRA1//GLRB//GNAQ//GRIA1//NRG1//NTF3//P2RY1//MINK1//KIF13A//CPEB1//BMPR2//BNIP3//STRN//WFS1//LRP8//C16ORF70//CPEB4//TMEM185A//URI1//SGCE//KIF5A//MAPK1//MAP2K4//CNN3//LPAR1//GPM6A//KCNA4//PRKAR2B//PSD//PTEN//FXR1//ZNF804A//NSF//SLC12A5// |
| GO:0005925 | focal adhesion | Cellular component | 36 | 393 | 970 | 18698 | 1.76576690013378 | 0.000696530290872385 | 0.0157452505226152 | 3.15705999212921 | 0.0371134020618557 | ADAM10//DLC1//TADA1//CNN3//EPHA2//FHL2//RAB21//PALLD//FLII//FLRT1//TES//GJA1//ITGA6//ITGA5//ITGAV//JAK1//RND3//MARCKS//MSN//PPP1R12A//LIMA1//ENAH//DCAF6//MAPK1//TWF1//ACTC1//TGFB1I1//YWHAG//AHNAK//ZFYVE21//PPFIA1//LAMTOR3//ADAM9//LMLN//KIF23//GIT2// |
| GO:0005912 | adherens junction | Cellular component | 45 | 528 | 970 | 18698 | 1.64286551077788 | 0.000760766486928674 | 0.0163513992180186 | 3.11874862706765 | 0.0463917525773196 | CNN3//DSC2//CADM2//CDH20//TMOD3//ITGA6//BMPR2//GJA1//ADAM10//DLC1//TADA1//EPHA2//FHL2//RAB21//PALLD//FLII//FLRT1//TES//ITGA5//ITGAV//JAK1//RND3//MARCKS//MSN//PPP1R12A//LIMA1//ENAH//DCAF6//MAPK1//TWF1//ACTC1//TGFB1I1//YWHAG//AHNAK//ZFYVE21//PPFIA1//LAMTOR3//ADAM9//LMLN//KIF23//GIT2//CTNND2//EFNA5//DLL1//NF2// |
| GO:0044297 | cell body | Cellular component | 44 | 513 | 970 | 18698 | 1.6533269025944 | 0.000761415563120771 | 0.0163513992180186 | 3.1183782505734 | 0.045360824742268 | STMN2//CHRM2//CNN3//LPAR1//EPHA5//EPHA7//FMR1//GLRA1//GPM6A//GRIA1//AGFG1//KIF5A//LRP6//SERPINI1//PRKAR2B//SLC12A5//KLHL14//SCN1A//BMPR2//SOS1//STRN//TGFB2//LRP8//FXR1//ZNF804A//FLRT1//PDE10A//RBFOX3//CTNND2//DMWD//EPHA4//WDFY3//NSMF//NEUROG1//TMEM100//MAPK1//MAP2K4//RDH10//GJB2//GNAQ//NRG1//NF2//P2RY1//ACTC1// |
| GO:0005924 | cell-substrate adherens junction | Cellular component | 36 | 396 | 970 | 18698 | 1.75238987816307 | 0.000799527552225665 | 0.016751077252728 | 3.09716656559705 | 0.0371134020618557 | ADAM10//DLC1//TADA1//CNN3//EPHA2//FHL2//RAB21//PALLD//FLII//FLRT1//TES//GJA1//ITGA6//ITGA5//ITGAV//JAK1//RND3//MARCKS//MSN//PPP1R12A//LIMA1//ENAH//DCAF6//MAPK1//TWF1//ACTC1//TGFB1I1//YWHAG//AHNAK//ZFYVE21//PPFIA1//LAMTOR3//ADAM9//LMLN//KIF23//GIT2// |
| GO:0098805 | whole membrane | Cellular component | 113 | 1630 | 970 | 18698 | 1.3363316678262 | 0.000831808022083142 | 0.0170124545468909 | 3.07997689554882 | 0.116494845360825 | PLEKHF2//SBF2//IQGAP2//RAB9B//ORMDL3//CLCN5//CLCN6//VPS37A//PIKFYVE//ZNRF2//APPL1//STEAP2//ARHGAP1//LDLR//MYO1B//RAB23//PMEPA1//RAB22A//KIF13A//SNX2//TSG101//ZFYVE21//LAMTOR3//ITM2B//GJA1//GRIA1//WNT7A//WASL//RAB38//ATG16L1//ULK2//CALCOCO2//CNIH1//SEC23A//SAR1B//MCFD2//SLC17A7//LHFPL2//TMX3//CKAP4//RAP1B//ADAM10//ITGAV//SNAP25//RAP2C//ENPP4//EPHA4//EPS15//RAB21//SNX5//LRP6//LDLRAD4//MMGT1//ZFYVE9//MMD//MCOLN2//CHMP3//LAPTM4A//RAB12//PLEKHB2//CHRM2//ACSL1//ACOX1//PEX5L//TMEM135//ACBD5//BCL2L11//SLC44A1//MTX3//NME1//PMAIP1//MIEF1//MOAP1//BNIP3//SNN//GNAQ//OSTM1//NSF//SPHK2//RHEB//GPR137B//VLDLR//AHNAK//CYBRD1//AP1S2//SLC32A1//SYT6//SLC17A6//C16ORF70//NDFIP2//WDFY3//CTSA//RRAGD//NECAP1//PSD//DLC1//SPRED1//EFNA5//MAPK1//BMPR2//TGFBR2//LRP8//LYPD6//CRK//S1PR1//SULF1//DLL1//RGMB//KDR//PRKAR2B//SLC2A4//TNF//STX6// |
| GO:0030054 | cell junction | Cellular component | 90 | 1247 | 970 | 18698 | 1.39123173968039 | 0.000855778257835827 | 0.0170956633367669 | 3.06763875153037 | 0.0927835051546392 | PIKFYVE//PDZD2//CD2AP//FLRT1//CLIC4//GRB2//ITGA5//NCK1//PKD2//TWF1//RAP1B//CNN3//DSC2//CADM2//CDH20//TMOD3//ITGA6//BMPR2//GJC1//GJA1//FRMD6//RAP2C//AHNAK//CTNND2//EFNA5//DLL1//NF2//CLDN11//STRN//SCN1A//ADAM10//DLC1//TADA1//EPHA2//FHL2//RAB21//PALLD//FLII//TES//ITGAV//JAK1//RND3//MARCKS//MSN//PPP1R12A//LIMA1//ENAH//DCAF6//MAPK1//ACTC1//TGFB1I1//YWHAG//ZFYVE21//PPFIA1//LAMTOR3//ADAM9//LMLN//KIF23//GIT2//GJB2//CHRM2//CHRM5//LYPD6//CBLN4//SYT6//ESCO2//DCP2//EPHA4//EPS8//ZNRF2//CPEB3//FMR1//DSTYK//NSMF//RGS17//GLRA1//GLRB//GRIA1//KDR//NFIA//MINK1//RAB23//PAK6//SLC17A7//SLC17A6//DSCAML1//CPEB1//SNAP25//CPEB4//PLAA// |
| GO:0008287 | protein serine/threonine phosphatase complex | Cellular component | 9 | 50 | 970 | 18698 | 3.46973195876289 | 0.000961926860991555 | 0.0183283936096155 | 3.016857947791 | 0.00927835051546392 | IER5//PPP2CA//STRN//NCK1//PPP1R15B//PPP3R1//PPP4R2//CNEP1R1//PPP1R12A// |
| GO:1903293 | phosphatase complex | Cellular component | 9 | 50 | 970 | 18698 | 3.46973195876289 | 0.000961926860991555 | 0.0183283936096155 | 3.016857947791 | 0.00927835051546392 | IER5//PPP2CA//STRN//NCK1//PPP1R15B//PPP3R1//PPP4R2//CNEP1R1//PPP1R12A// |
| GO:0030055 | cell-substrate junction | Cellular component | 36 | 401 | 970 | 18698 | 1.73053963030568 | 0.00100117314467231 | 0.0183283936096155 | 2.99949080836171 | 0.0371134020618557 | ITGA6//ADAM10//DLC1//TADA1//CNN3//EPHA2//FHL2//RAB21//PALLD//FLII//FLRT1//TES//GJA1//ITGA5//ITGAV//JAK1//RND3//MARCKS//MSN//PPP1R12A//LIMA1//ENAH//DCAF6//MAPK1//TWF1//ACTC1//TGFB1I1//YWHAG//AHNAK//ZFYVE21//PPFIA1//LAMTOR3//ADAM9//LMLN//KIF23//GIT2// |
| GO:0030014 | CCR4-NOT complex | Cellular component | 5 | 16 | 970 | 18698 | 6.02384020618557 | 0.0010051134610475 | 0.0183283936096155 | 2.9977849106559 | 0.00515463917525773 | CNOT7//TOB1//CPEB3//CNOT6L//CNOT6// |
| GO:0032279 | asymmetric synapse | Cellular component | 24 | 232 | 970 | 18698 | 1.99409882687522 | 0.00102417100496105 | 0.0183283936096155 | 2.98962752352571 | 0.0247422680412371 | ADAM10//CNN3//EPHA4//EPS8//CPEB3//FMR1//NSMF//NPTN//GRIA1//NEFH//NSF//P2RY1//MINK1//PSD//CPEB1//BMPR2//BNIP3//SOS1//SPOCK1//STRN//LRP8//CPEB4//FXR1//CHRM2// |
| GO:0030424 | axon | Cellular component | 45 | 538 | 970 | 18698 | 1.6123289771203 | 0.00111622634655715 | 0.0195681312590325 | 2.95224773094196 | 0.0463917525773196 | NFIB//SLC17A7//STMN2//EPS8//PALLD//FMR1//OTX2//TSHZ3//SNAP25//TSC1//CPEB4//ZNF804A//NRG1//SCN1A//SPOCK1//STX6//PFN2//CHRM2//EPHA4//GPM6A//GRIA1//SLC32A1//RAB21//SYBU//IQCJ-SCHIP1//MTPN//EPHA5//WDFY3//LPAR3//CADM2//SACS//TNFRSF21//KCNA4//NEFM//NEFH//NTF3//MINK1//MAPK1//KIF13A//MAP2K4//SLC6A1//TGFB2//LRP8//FXR1//FEZ2// |
| GO:0044463 | cell projection part | Cellular component | 97 | 1377 | 970 | 18698 | 1.35787944807553 | 0.00118697444806227 | 0.0199923735467743 | 2.9255586299797 | 0.1 | TRIM3//CHRM2//SLC32A1//CTNND2//DMWD//EPHA4//EPHA5//EPHA7//CPEB3//FMR1//ZDHHC5//NSMF//SACS//NPTN//GLRA1//GLRB//GNAQ//GRIA1//NRG1//NTF3//P2RY1//MINK1//KIF13A//CPEB1//BMPR2//BNIP3//STRN//WFS1//LRP8//C16ORF70//CPEB4//TMEM185A//URI1//EPS8//CNN3//LPAR1//GPM6A//KCNA4//PRKAR2B//PSD//PTEN//FXR1//ZNF804A//NSF//SLC12A5//SCRG1//FLRT1//EPHA2//ITGAV//SLC6A14//CYBRD1//NF2//MSN//DLC1//FGD5//ITGA5//NME1//ARHGEF4//PLEK//SNTG1//TWF1//PLEKHA1//SGCE//NAPEPLD//DRD1//TMEM17//EPS15//PKD2//KIF5A//MAPK1//MAP2K4//RAB21//SYBU//STMN2//PALLD//OTX2//TSHZ3//SNAP25//TSC1//SCN1A//SPOCK1//STX6//PFN2//DDX6//GNB5//ATG16L1//TOPORS//CETN1//RAB28//C5ORF30//NEDD1//DZIP1//CEP41//SQSTM1//DYNLL2//PRKACB//CEP128// |
| GO:0120038 | plasma membrane bounded cell projection part | Cellular component | 97 | 1377 | 970 | 18698 | 1.35787944807553 | 0.00118697444806227 | 0.0199923735467743 | 2.9255586299797 | 0.1 | TRIM3//CHRM2//SLC32A1//CTNND2//DMWD//EPHA4//EPHA5//EPHA7//CPEB3//FMR1//ZDHHC5//NSMF//SACS//NPTN//GLRA1//GLRB//GNAQ//GRIA1//NRG1//NTF3//P2RY1//MINK1//KIF13A//CPEB1//BMPR2//BNIP3//STRN//WFS1//LRP8//C16ORF70//CPEB4//TMEM185A//URI1//EPS8//CNN3//LPAR1//GPM6A//KCNA4//PRKAR2B//PSD//PTEN//FXR1//ZNF804A//NSF//SLC12A5//SCRG1//FLRT1//EPHA2//ITGAV//SLC6A14//CYBRD1//NF2//MSN//DLC1//FGD5//ITGA5//NME1//ARHGEF4//PLEK//SNTG1//TWF1//PLEKHA1//DRD1//TMEM17//EPS15//PKD2//STMN2//PALLD//OTX2//TSHZ3//SNAP25//TSC1//SCN1A//SPOCK1//STX6//PFN2//RAB21//SYBU//DDX6//GNB5//ATG16L1//KIF5A//RAB28//C5ORF30//TOPORS//NEDD1//DZIP1//CEP41//SQSTM1//DYNLL2//PRKACB//CEP128//SGCE//NAPEPLD//MAPK1//MAP2K4//CETN1// |
| GO:0098984 | neuron to neuron synapse | Cellular component | 24 | 235 | 970 | 18698 | 1.9686422461066 | 0.00122329224441098 | 0.0202078468836352 | 2.91246977765444 | 0.0247422680412371 | CHRM2//ADAM10//CNN3//EPHA4//EPS8//CPEB3//FMR1//NSMF//NPTN//GRIA1//NEFH//NSF//P2RY1//MINK1//PSD//CPEB1//BMPR2//BNIP3//SOS1//SPOCK1//STRN//LRP8//CPEB4//FXR1// |
| GO:0070161 | anchoring junction | Cellular component | 45 | 546 | 970 | 18698 | 1.58870510932367 | 0.00149872745233727 | 0.0242906958784474 | 2.82427733760396 | 0.0463917525773196 | CTNND2//EFNA5//DLL1//NF2//DSC2//CNN3//CADM2//CDH20//TMOD3//ITGA6//BMPR2//GJA1//ADAM10//DLC1//TADA1//EPHA2//FHL2//RAB21//PALLD//FLII//FLRT1//TES//ITGA5//ITGAV//JAK1//RND3//MARCKS//MSN//PPP1R12A//LIMA1//ENAH//DCAF6//MAPK1//TWF1//ACTC1//TGFB1I1//YWHAG//AHNAK//ZFYVE21//PPFIA1//LAMTOR3//ADAM9//LMLN//KIF23//GIT2// |
| GO:0030175 | filopodium | Cellular component | 13 | 98 | 970 | 18698 | 2.55705869976857 | 0.00163224281174954 | 0.0259647513943121 | 2.7872152353131 | 0.0134020618556701 | ITGAV//NF2//FMR1//IQGAP2//FGD4//EPHA4//GPM6A//ITGA6//MYO1B//MSN//ENAH//TWF1//ACTC1// |
| GO:0014069 | postsynaptic density | Cellular component | 23 | 227 | 970 | 18698 | 1.95310413733594 | 0.00169812708987469 | 0.0265216576400429 | 2.77002980975413 | 0.0237113402061856 | GRIA1//ADAM10//CNN3//EPHA4//EPS8//CPEB3//FMR1//NSMF//NPTN//NEFH//NSF//P2RY1//MINK1//PSD//CPEB1//BMPR2//BNIP3//SOS1//SPOCK1//STRN//LRP8//CPEB4//FXR1// |
| GO:0099572 | postsynaptic specialization | Cellular component | 23 | 229 | 970 | 18698 | 1.9360464592806 | 0.00190409286507871 | 0.0292074244839752 | 2.72031187432983 | 0.0237113402061856 | ADAM10//CNN3//EPHA4//EPS8//CPEB3//FMR1//NSMF//NPTN//GRIA1//NEFH//NSF//P2RY1//MINK1//PSD//CPEB1//BMPR2//BNIP3//SOS1//SPOCK1//STRN//LRP8//CPEB4//FXR1// |
| GO:0010008 | endosome membrane | Cellular component | 38 | 451 | 970 | 18698 | 1.62416622854139 | 0.00230817800368183 | 0.0347846474589946 | 2.63673070199992 | 0.0391752577319588 | VPS37A//TSG101//CHMP3//SNX5//PIKFYVE//EPHA4//EPS15//RAB21//APPL1//GRIA1//LRP6//PMEPA1//SNX2//LDLRAD4//PLEKHF2//MMGT1//ZFYVE9//MMD//MCOLN2//LAPTM4A//LDLR//RAB12//PLEKHB2//RAP2C//NDFIP2//LAMTOR3//PSD//CLCN5//CLCN6//ZNRF2//STEAP2//ARHGAP1//MYO1B//RAB23//RAB22A//KIF13A//ZFYVE21//ITM2B// |
| GO:0016234 | inclusion body | Cellular component | 11 | 80 | 970 | 18698 | 2.65048969072165 | 0.00272571172353046 | 0.0403687305260804 | 2.56452007788516 | 0.011340206185567 | EPS15//KLHL14//PRDM16//SQSTM1//EDEM1//ATXN3//ATXN1//NEFM//NEFH//WDFY3//BAG5// |
| GO:0043204 | perikaryon | Cellular component | 14 | 118 | 970 | 18698 | 2.28701729861961 | 0.00319591676975852 | 0.0465303814444503 | 2.4954045394035 | 0.0144329896907217 | PDE10A//RBFOX3//CTNND2//DMWD//EPHA4//WDFY3//FMR1//NSMF//GLRA1//NEUROG1//SERPINI1//TMEM100//MAPK1//MAP2K4// |
| GO:0043025 | neuronal cell body | Cellular component | 37 | 449 | 970 | 18698 | 1.5884692214084 | 0.00378475329011106 | 0.05418505127009 | 2.42196242481264 | 0.0381443298969072 | FLRT1//PDE10A//RBFOX3//CTNND2//DMWD//EPHA4//WDFY3//FMR1//NSMF//GLRA1//NEUROG1//SERPINI1//TMEM100//MAPK1//MAP2K4//STMN2//CHRM2//CNN3//LPAR1//EPHA5//EPHA7//GPM6A//GRIA1//AGFG1//KIF5A//LRP6//PRKAR2B//SLC12A5//KLHL14//SCN1A//BMPR2//SOS1//STRN//TGFB2//LRP8//FXR1//ZNF804A// |
| GO:0098794 | postsynapse | Cellular component | 37 | 450 | 970 | 18698 | 1.58493928980527 | 0.00392484151171747 | 0.0552694894846772 | 2.40617787572796 | 0.0381443298969072 | CNN3//LPAR1//EPHA4//FMR1//GPM6A//GRIA1//KCNA4//PRKAR2B//PSD//PTEN//STRN//CPEB4//FXR1//ZNF804A//CHRM2//CHRM5//EPHA7//CPEB3//NSMF//GLRA1//GLRB//P2RY1//MINK1//CPEB1//ADAM10//EPS8//NPTN//NEFH//NSF//BMPR2//BNIP3//SOS1//SPOCK1//LRP8//RAB3GAP1//SLC17A7//SNAP25// |
| GO:0005815 | microtubule organizing center | Cellular component | 53 | 704 | 970 | 18698 | 1.4511978678538 | 0.00421248292883298 | 0.0583632715462505 | 2.37546184610367 | 0.054639175257732 | MZT1//CDK6//DCTN6//CETN1//NEDD1//VPS37A//DYNLL2//PPP4R2//DYNC1LI2//E2F1//CLIC4//MARCKS//PPP1R12A//NME1//RAB23//PRKACB//PRKAR2B//MDM1//SLAIN2//RRAGD//CLIP1//KIF13A//CAPRIN2//TFAP2A//CEP97//CALM2//PCGF5//MASTL//CDC14A//KAT2B//HAUS8//CAMK2N2//KIF23//CEP41//TOPORS//DZIP1//PKD2//RAB28//CEP128//FRY//TACC2//AKAP11//LCA5//FLII//LATS2//YTHDF2//CC2D1A//PXK//C7ORF43//MAPK1//TSG101//EVI5//FAM110B// |
| GO:0017053 | transcriptional repressor complex | Cellular component | 11 | 85 | 970 | 18698 | 2.49457853244391 | 0.0043920798914432 | 0.0598856607420589 | 2.35732976871794 | 0.011340206185567 | BAHD1//APPL1//RBBP7//JUN//RLIM//MIER1//ARID4A//RBBP8//REST//PRDM16//TBL1XR1// |
| GO:0005856 | cytoskeleton | Cellular component | 136 | 2126 | 970 | 18698 | 1.23310219084288 | 0.00530678922220849 | 0.070758716344433 | 2.2751681615603 | 0.14020618556701 | CORO2B//CDC42EP3//CFL2//IQGAP2//ZNF268//KLHL2//CNN3//CRK//PALLD//CD2AP//CLIC4//ARHGAP6//MARCKS//PPP1R12A//LIMA1//CENPQ//TMEM63B//RAB22A//TWF1//KLHL14//AHNAK//CALD1//WASL//STK17B//TACC2//PPP2CA//MAP7D1//SLAIN2//MID1IP1//CLIP1//NSMF//MPP1//TMOD1//NEFM//ZNF131//SESTD1//DLC1//NF2//TMOD3//DCTN6//MYO1B//ACTC1//TSC1//MTPN//DYNLL2//KAT2B//FRY//AKAP11//LCA5//DZIP1//FLII//LATS2//YTHDF2//CC2D1A//PXK//C7ORF43//MAPK1//TSG101//EVI5//CEP97//FAM110B//MZT1//PTP4A1//KBTBD8//KIF23//DCX//DYNC1LI2//DPYSL2//REEP3//WDR47//RASSF3//KIF5A//SYBU//MDM1//KIF13A//CCT6A//HAUS8//RANBP9//LRP8//GJA1//KRT27//NME1//NEDD1//TOPORS//CETN1//CEP128//CALM2//CDC14A//DDX6//BCL2L11//ATG16L1//CDK6//VPS37A//PPP4R2//E2F1//RAB23//PRKACB//PRKAR2B//RRAGD//CAPRIN2//TFAP2A//PCGF5//MASTL//CAMK2N2//CEP41//TBL1XR1//SERP1//NEFH//PKD2//RAB28//MAPRE3//HECW2//ZNF804A//CALCOCO2//WASF3//CKAP4//PTPN21//FGD4//SLAIN1//FRMD6//FGD5//KLHL3//MYLIP//HSBP1//JAK1//MSN//PFN2//SNTG1//ENAH//PTPN4//SNAP25//TGFB1I1//RNF128//CCDC6//SGCE//TRIP10// |
| GO:0031901 | early endosome membrane | Cellular component | 15 | 138 | 970 | 18698 | 2.09524876736889 | 0.00535426840790238 | 0.070758716344433 | 2.27129986154858 | 0.0154639175257732 | RAB21//PIKFYVE//EPHA4//EPS15//APPL1//SNX5//GRIA1//LRP6//PMEPA1//SNX2//TSG101//LDLRAD4//PLEKHF2//MMGT1//ZFYVE9// |
| GO:0031256 | leading edge membrane | Cellular component | 17 | 165 | 970 | 18698 | 1.98604186191815 | 0.00543833615559949 | 0.070780769055454 | 2.26453395121527 | 0.0175257731958763 | EPHA2//ITGAV//DLC1//FGD5//EPS8//ITGA5//NF2//NME1//ARHGEF4//PLEK//SNTG1//PSD//TWF1//PLEKHA1//NRG1//SGCE//GRIA1// |
| GO:0044440 | endosomal part | Cellular component | 39 | 496 | 970 | 18698 | 1.51567592284669 | 0.00655277881313468 | 0.0840124925445178 | 2.18357449126198 | 0.0402061855670103 | CLCN5//CLCN6//VPS37A//PIKFYVE//ZNRF2//APPL1//STEAP2//ARHGAP1//LDLR//MYO1B//RAB23//PMEPA1//RAB22A//KIF13A//SNX2//TSG101//ZFYVE21//LAMTOR3//ITM2B//SNX5//RRAGD//EPHA4//EPS15//RAB21//GRIA1//LRP6//LDLRAD4//PLEKHF2//MMGT1//ZFYVE9//MMD//MCOLN2//CHMP3//LAPTM4A//RAB12//PLEKHB2//RAP2C//PSD//NDFIP2// |
| GO:0005905 | clathrin-coated pit | Cellular component | 9 | 67 | 970 | 18698 | 2.58935220803201 | 0.0074982591426466 | 0.0947206559343151 | 2.12503955427501 | 0.00927835051546392 | EPS15//FCHO2//NECAP1//EPN1//LRP12//LDLR//SLC2A4//VLDLR//AP1S2// |
| GO:0005776 | autophagosome | Cellular component | 11 | 92 | 970 | 18698 | 2.30477364410578 | 0.00798258071300378 | 0.0992712946093779 | 2.09785668155332 | 0.011340206185567 | CALCOCO2//ATG16L1//SQSTM1//WDFY3//RAB12//TBC1D12//NRBF2//RAB23//FYCO1//PLEKHF2//TBC1D17// |
| GO:0034399 | nuclear periphery | Cellular component | 14 | 131 | 970 | 18698 | 2.06006138348942 | 0.00808962819866875 | 0.0992712946093779 | 2.09207143821184 | 0.0144329896907217 | NUP35//CEBPB//CFL2//MORC3//CLIC4//NSMF//ATXN3//MYB//PIAS4//RAD21//ATXN1//TGFB1I1//RANBP6//IPO5// |
| GO:0044798 | nuclear transcription factor complex | Cellular component | 18 | 187 | 970 | 18698 | 1.85547163570208 | 0.00862974567342831 | 0.102744707995803 | 2.0640020031555 | 0.0185567010309278 | SNAPC1//E2F3//NR5A2//NEUROD1//PPARG//RARB//STAT3//THRA//HAND1//GTF2E1//E2F1//JUN//CEBPB//TEAD1//BDP1//SOX17//SOX4//KLF4// |
| GO:0045202 | synapse | Cellular component | 62 | 880 | 970 | 18698 | 1.35810215557638 | 0.00876976282074277 | 0.102744707995803 | 2.05701215201999 | 0.0639175257731959 | EPHA4//EPHA7//NRG1//SPOCK1//SLC17A7//SLC17A6//SLC32A1//NPTN//GLRA1//GLRB//CHRM2//ADAM10//CNN3//EPS8//CPEB3//FMR1//NSMF//GRIA1//NEFH//NSF//P2RY1//MINK1//PSD//CPEB1//BMPR2//BNIP3//SOS1//STRN//LRP8//CPEB4//FXR1//STX2//NTF3//SNAP25//STX6//SYT6//C16ORF70//ZNRF2//FOSL1//PFN2//LPAR1//GPM6A//KCNA4//PRKAR2B//PTEN//ZNF804A//CHRM5//PPFIA1//KCNJ10//WNT7A//RAB3GAP1//FCHO2//LYPD6//CPEB2//CBLN4//CADM2//RGS17//ITGA5//LRP6//ENAH//DSCAML1//PLAA// |
| GO:0005783 | endoplasmic reticulum | Cellular component | 119 | 1859 | 970 | 18698 | 1.23393133432785 | 0.00883143750636652 | 0.102744707995803 | 2.05396859990253 | 0.122680412371134 | CKAP4//EPHA5//RASD1//EDN1//ASPH//PIGA//ARL6IP1//DERL2//ADAM10//CRTAP//COL1A2//MIA3//DNAJB9//FAM20C//ANO8//WFS1//WNT7A//KDELC1//GJC1//CNIH1//RTN3//CDS1//SEC23A//OSBPL5//TBC1D20//PPM1L//VKORC1L1//RDH10//SPTSSA//DRD1//SMIM14//ACSL1//REEP3//RAB3GAP1//RAB21//SLC35D1//UBXN4//FLRT1//LCLAT1//GJA1//SERP1//RNF180//GRIA1//SHISA2//ATXN3//NR3C2//IER3IP1//SAR1B//ERGIC2//JKAMP//PKD2//TMX3//UGT1A10//UGT1A8//UGT1A7//UGT1A6//UGT1A5//UGT1A9//UGT1A4//UGT1A1//UGT1A3//TMED9//KLHL14//STIM2//RHEB//MSMO1//XYLT2//APOO//SCD5//ELOVL7//SGPP1//GSG1//MCFD2//LBR//DNAJB12//RTN1//ELOVL4//EDEM1//NPLOC4//ORMDL3//MMGT1//SYT6//MAP3K5//UBXN2B//FGD5//TMEM64//EPHA4//PDZD2//SULF1//RAB38//GLRA1//ZDHHC1//ITGA5//KDR//FIBIN//LAMA3//LRP6//FAM19A1//MAN1A1//NCK1//ACO1//NDFIP2//CTSA//DUS2//TMEM100//RGMA//PHTF2//BNIP3//HSPA13//TMF1//PTP4A1//RNF128//PLEKHF2//CPEB4//CAMLG//B3GNT5//PPP1R15B//B3GALT2//SQSTM1// |
| GO:0031974 | membrane-enclosed lumen | Cellular component | 302 | 5188 | 970 | 18698 | 1.12209698829178 | 0.00909033504968687 | 0.102744707995803 | 2.04142010934695 | 0.311340206185567 | IDH1//IMPDH1//SERPINI1//FASTK//PABPC5//ATXN3//BCAT2//ADAM10//CRTAP//CKAP4//COL1A2//MIA3//DNAJB9//FAM20C//ANO8//WFS1//WNT7A//KDELC1//OGN//HS3ST1//GNS//MAPK1//CHCHD10//NLN//THOP1//ACOX1//C6ORF120//CTSA//TTR//PPT2//EDN1//CDC37L1//IGF1//NHLRC2//TGFB2//VEGFB//ZBTB18//JUN//HMGA2//CDK6//TOPORS//GDF11//STX6//CDK8//CDKN1A//IRF9//CEBPB//TACC2//POLR3G//KHDRBS3//PTGES3//MAP3K2//ZMYND11//TCERG1//COPS8//DNAJB4//FZD10//DUSP10//EGLN3//KLF12//ACOT7//TADA1//NUP35//VPS37A//PTPDC1//CRKL//PDIK1L//PPP4R2//RNF38//SH3D19//CREBRF//ESCO2//GADD45A//DCP2//DNMT3B//DUSP5//E2F1//E2F3//E2F5//S1PR1//ZNF367//EGR2//ELAVL2//CC2D1B//ELK3//ESR1//ETS1//EZH1//FHL2//DZIP1//BAHD1//FOXC1//KDM2A//KLHDC10//FLII//CIC//FMR1//USP24//ZFPM2//SLC44A1//MORC3//NR5A2//ST6GALNAC3//NSMF//GATA6//HBP1//GJA1//GOLGA3//MKNK2//GRB2//ATAD2//GTF2E1//GRHL1//NRBF2//NRG1//HIVEP2//HOXA3//HOXD1//HSBP1//IRF1//AR//JARID2//ZBTB34//MBNL1//MDM4//MEF2D//NR3C2//MYB//PPP1R12A//NEUROD1//NFIA//NFE2//NPAS2//NPAT//NR4A2//DCAF8//RLIM//KLF3//CTDSPL2//PIAS4//ARID4B//BRWD1//POU4F1//PPARG//BNC2//ESRP1//CENPQ//UBE2W//FBXW7//PPP3R1//ELMOD1//PRKAA1//INTS8//NPLOC4//PRKACB//TRERF1//BDP1//DCAF6//FEM1C//PTEN//PTHLH//REXO1//TSHZ3//USP28//ZBTB4//MIER1//PTPN4//RRAGD//RAD21//RARB//ARID4A//RBBP7//RBBP8//PLEKHA1//RBL2//REST//REV3L//RGS3//RNF2//RNF4//ATXN1//SCN1A//PRDM16//SRSF2//SOX17//ZMAT3//CPEB1//SHOX//NABP1//SKP1//UBE2Z//BMPR2//SMARCD2//SNAPC1//BNIP3//SOX4//STAT3//STAT4//ZEB1//TEAD1//TFAP2A//TFAP2C//TGIF1//THRA//UBE2I//LDLRAD4//ZNF131//ZNF217//DEK//BAG6//CENPO//TBL1XR1//SNIP1//NR4A3//ADAM12//FOSL1//SGPP1//TCF7L1//ARID5B//PCGF5//LCOR//GLYR1//DPY30//MASTL//NUDCD1//USP45//CDC14A//RTCA//RUNX3//CBFB//CDC23//URI1//KAT2B//SQSTM1//CCNC//CCND2//RPS6KA4//BTAF1//CCNE2//ACBD5//WDR20//RPS6KA5//KLF4//TRIP10//HAND1//KIF23//CLOCK//KMT2B//GIT2//USP3//AKAP11//KLF6//DDX6//PPM1E//WDFY3//SERTAD3//MDFIC//MNX1//IPO5//YPEL2//NF2//NOVA1//ERGIC2//CMPK1//PIK3CB//CDCA7L//SLC14A1//TSG101//ZNF655//FXR1//PER2//COX7A2L//RANBP6//CFL2//CLIC4//TGFB1I1//KLF2//H2AFZ//HAND2//SYCP3//ANP32E//APPL1//BRMS1L//DR1//FAM118B//CDK19//HIPK3//FLI1//CNOT7//BHLHE40//CRY2//DENND1B//MEOX2//MPP1//SLC2A4RG//RBM25//EPC2//SESN1//NFIB//CC2D1A//PAK6//BTBD10// |
| GO:0043233 | organelle lumen | Cellular component | 302 | 5188 | 970 | 18698 | 1.12209698829178 | 0.00909033504968687 | 0.102744707995803 | 2.04142010934695 | 0.311340206185567 | SERPINI1//FASTK//PABPC5//ATXN3//BCAT2//ADAM10//CRTAP//CKAP4//COL1A2//MIA3//DNAJB9//FAM20C//ANO8//WFS1//WNT7A//KDELC1//OGN//HS3ST1//IDH1//GNS//IMPDH1//MAPK1//C6ORF120//CTSA//TTR//PPT2//EDN1//ACOX1//CHCHD10//NLN//THOP1//CDC37L1//IGF1//NHLRC2//TGFB2//VEGFB//ZBTB18//JUN//HMGA2//CDK6//TOPORS//GDF11//STX6//CDK8//CDKN1A//IRF9//CEBPB//TACC2//POLR3G//KHDRBS3//PTGES3//MAP3K2//ZMYND11//TCERG1//COPS8//DNAJB4//FZD10//DUSP10//EGLN3//KLF12//ACOT7//TADA1//NUP35//VPS37A//PTPDC1//CRKL//PDIK1L//PPP4R2//RNF38//SH3D19//CREBRF//ESCO2//GADD45A//DCP2//DNMT3B//DUSP5//E2F1//E2F3//E2F5//S1PR1//ZNF367//EGR2//ELAVL2//CC2D1B//ELK3//ESR1//ETS1//EZH1//FHL2//DZIP1//BAHD1//FOXC1//KDM2A//KLHDC10//FLII//CIC//FMR1//USP24//ZFPM2//SLC44A1//MORC3//NR5A2//ST6GALNAC3//NSMF//GATA6//HBP1//GJA1//GOLGA3//MKNK2//GRB2//ATAD2//GTF2E1//GRHL1//NRBF2//NRG1//HIVEP2//HOXA3//HOXD1//HSBP1//IRF1//AR//JARID2//ZBTB34//MBNL1//MDM4//MEF2D//NR3C2//MYB//PPP1R12A//NEUROD1//NFIA//NFE2//NPAS2//NPAT//NR4A2//DCAF8//RLIM//KLF3//CTDSPL2//PIAS4//ARID4B//BRWD1//POU4F1//PPARG//BNC2//ESRP1//CENPQ//UBE2W//FBXW7//PPP3R1//ELMOD1//PRKAA1//INTS8//NPLOC4//PRKACB//TRERF1//BDP1//DCAF6//FEM1C//PTEN//PTHLH//REXO1//TSHZ3//USP28//ZBTB4//MIER1//PTPN4//RRAGD//RAD21//RARB//ARID4A//RBBP7//RBBP8//PLEKHA1//RBL2//REST//REV3L//RGS3//RNF2//RNF4//ATXN1//SCN1A//PRDM16//SRSF2//SOX17//ZMAT3//CPEB1//SHOX//NABP1//SKP1//UBE2Z//BMPR2//SMARCD2//SNAPC1//BNIP3//SOX4//STAT3//STAT4//ZEB1//TEAD1//TFAP2A//TFAP2C//TGIF1//THRA//UBE2I//LDLRAD4//ZNF131//ZNF217//DEK//BAG6//CENPO//TBL1XR1//SNIP1//NR4A3//ADAM12//FOSL1//SGPP1//TCF7L1//ARID5B//PCGF5//LCOR//GLYR1//DPY30//MASTL//NUDCD1//USP45//CDC14A//RTCA//RUNX3//CBFB//CDC23//URI1//KAT2B//SQSTM1//CCNC//CCND2//RPS6KA4//BTAF1//CCNE2//ACBD5//WDR20//RPS6KA5//KLF4//TRIP10//HAND1//KIF23//CLOCK//KMT2B//GIT2//USP3//AKAP11//KLF6//DDX6//PPM1E//WDFY3//SERTAD3//MDFIC//MNX1//IPO5//YPEL2//NF2//NOVA1//ERGIC2//CMPK1//PIK3CB//CDCA7L//SLC14A1//TSG101//ZNF655//FXR1//PER2//COX7A2L//RANBP6//CFL2//CLIC4//TGFB1I1//KLF2//H2AFZ//HAND2//SYCP3//ANP32E//APPL1//BRMS1L//DR1//FAM118B//CDK19//HIPK3//FLI1//CNOT7//BHLHE40//CRY2//DENND1B//MEOX2//MPP1//SLC2A4RG//RBM25//EPC2//SESN1//NFIB//CC2D1A//PAK6//BTBD10// |
| GO:0070013 | intracellular organelle lumen | Cellular component | 302 | 5188 | 970 | 18698 | 1.12209698829178 | 0.00909033504968687 | 0.102744707995803 | 2.04142010934695 | 0.311340206185567 | FASTK//PABPC5//ATXN3//BCAT2//ADAM10//CRTAP//CKAP4//COL1A2//MIA3//DNAJB9//FAM20C//ANO8//WFS1//WNT7A//KDELC1//OGN//HS3ST1//SERPINI1//IDH1//GNS//IMPDH1//MAPK1//C6ORF120//CTSA//TTR//PPT2//EDN1//ACOX1//CHCHD10//NLN//THOP1//CDC37L1//IGF1//NHLRC2//TGFB2//VEGFB//ZBTB18//JUN//HMGA2//CDK6//TOPORS//GDF11//STX6//CDK8//CDKN1A//IRF9//CEBPB//TACC2//POLR3G//KHDRBS3//PTGES3//MAP3K2//ZMYND11//TCERG1//COPS8//DNAJB4//FZD10//DUSP10//EGLN3//KLF12//ACOT7//TADA1//NUP35//VPS37A//PTPDC1//CRKL//PDIK1L//PPP4R2//RNF38//SH3D19//CREBRF//ESCO2//GADD45A//DCP2//DNMT3B//DUSP5//E2F1//E2F3//E2F5//S1PR1//ZNF367//EGR2//ELAVL2//CC2D1B//ELK3//ESR1//ETS1//EZH1//FHL2//DZIP1//BAHD1//FOXC1//KDM2A//KLHDC10//FLII//CIC//FMR1//USP24//ZFPM2//SLC44A1//MORC3//NR5A2//ST6GALNAC3//NSMF//GATA6//HBP1//GJA1//GOLGA3//MKNK2//GRB2//ATAD2//GTF2E1//GRHL1//NRBF2//NRG1//HIVEP2//HOXA3//HOXD1//HSBP1//IRF1//AR//JARID2//ZBTB34//MBNL1//MDM4//MEF2D//NR3C2//MYB//PPP1R12A//NEUROD1//NFIA//NFE2//NPAS2//NPAT//NR4A2//DCAF8//RLIM//KLF3//CTDSPL2//PIAS4//ARID4B//BRWD1//POU4F1//PPARG//BNC2//ESRP1//CENPQ//UBE2W//FBXW7//PPP3R1//ELMOD1//PRKAA1//INTS8//NPLOC4//PRKACB//TRERF1//BDP1//DCAF6//FEM1C//PTEN//PTHLH//REXO1//TSHZ3//USP28//ZBTB4//MIER1//PTPN4//RRAGD//RAD21//RARB//ARID4A//RBBP7//RBBP8//PLEKHA1//RBL2//REST//REV3L//RGS3//RNF2//RNF4//ATXN1//SCN1A//PRDM16//SRSF2//SOX17//ZMAT3//CPEB1//SHOX//NABP1//SKP1//UBE2Z//BMPR2//SMARCD2//SNAPC1//BNIP3//SOX4//STAT3//STAT4//ZEB1//TEAD1//TFAP2A//TFAP2C//TGIF1//THRA//UBE2I//LDLRAD4//ZNF131//ZNF217//DEK//BAG6//CENPO//TBL1XR1//SNIP1//NR4A3//ADAM12//FOSL1//SGPP1//TCF7L1//ARID5B//PCGF5//LCOR//GLYR1//DPY30//MASTL//NUDCD1//USP45//CDC14A//RTCA//RUNX3//CBFB//CDC23//URI1//KAT2B//SQSTM1//CCNC//CCND2//RPS6KA4//BTAF1//CCNE2//ACBD5//WDR20//RPS6KA5//KLF4//TRIP10//HAND1//KIF23//CLOCK//KMT2B//GIT2//USP3//AKAP11//KLF6//DDX6//PPM1E//WDFY3//SERTAD3//MDFIC//MNX1//IPO5//YPEL2//NF2//NOVA1//ERGIC2//CMPK1//PIK3CB//CDCA7L//SLC14A1//TSG101//ZNF655//FXR1//PER2//COX7A2L//RANBP6//CFL2//CLIC4//TGFB1I1//KLF2//H2AFZ//HAND2//SYCP3//ANP32E//APPL1//BRMS1L//DR1//FAM118B//CDK19//HIPK3//FLI1//CNOT7//BHLHE40//CRY2//DENND1B//MEOX2//MPP1//SLC2A4RG//RBM25//EPC2//SESN1//NFIB//CC2D1A//PAK6//BTBD10// |
| GO:0005789 | endoplasmic reticulum membrane | Cellular component | 72 | 1050 | 970 | 18698 | 1.32180265095729 | 0.00921228788794794 | 0.102770847996718 | 2.0356324986069 | 0.0742268041237113 | PIGA//ASPH//NPLOC4//MAP3K5//DERL2//SPTSSA//ORMDL3//ARL6IP1//LBR//DNAJB12//RTN1//ELOVL4//WFS1//ELOVL7//EDEM1//PKD2//MMGT1//GJC1//CNIH1//RTN3//CDS1//SEC23A//CKAP4//OSBPL5//TBC1D20//PPM1L//VKORC1L1//RDH10//DRD1//SMIM14//ACSL1//REEP3//RAB3GAP1//RAB21//SLC35D1//UBXN4//FLRT1//LCLAT1//GJA1//SERP1//RNF180//GRIA1//MIA3//SHISA2//DNAJB9//ATXN3//NR3C2//IER3IP1//SAR1B//ERGIC2//JKAMP//TMX3//UGT1A10//UGT1A8//UGT1A7//UGT1A6//UGT1A5//UGT1A9//UGT1A4//UGT1A1//UGT1A3//TMED9//KLHL14//STIM2//RHEB//MSMO1//XYLT2//APOO//SCD5//SGPP1//GSG1//MCFD2// |
| GO:0043198 | dendritic shaft | Cellular component | 6 | 36 | 970 | 18698 | 3.2127147766323 | 0.00983317016923957 | 0.10768748923058 | 2.00730644503168 | 0.00618556701030928 | LPAR1//EPHA4//NSF//PRKAR2B//SLC12A5//ZNF804A// |
| GO:0098827 | endoplasmic reticulum subcompartment | Cellular component | 72 | 1054 | 970 | 18698 | 1.31678632211115 | 0.010016141522266 | 0.10768748923058 | 1.99929954776134 | 0.0742268041237113 | GJC1//CNIH1//RTN3//CDS1//SEC23A//CKAP4//OSBPL5//TBC1D20//PPM1L//VKORC1L1//RDH10//SPTSSA//DRD1//SMIM14//ACSL1//REEP3//RAB3GAP1//RAB21//SLC35D1//UBXN4//ARL6IP1//FLRT1//LCLAT1//GJA1//SERP1//RNF180//GRIA1//MIA3//SHISA2//DNAJB9//ATXN3//NR3C2//ASPH//DERL2//IER3IP1//SAR1B//ERGIC2//JKAMP//PIGA//PKD2//TMX3//UGT1A10//UGT1A8//UGT1A7//UGT1A6//UGT1A5//UGT1A9//UGT1A4//UGT1A1//UGT1A3//TMED9//KLHL14//STIM2//RHEB//MSMO1//XYLT2//WFS1//APOO//SCD5//ELOVL7//SGPP1//GSG1//MCFD2//NPLOC4//MAP3K5//ORMDL3//LBR//DNAJB12//RTN1//ELOVL4//EDEM1//MMGT1// |
| GO:0042175 | nuclear outer membrane-endoplasmic reticulum membrane network | Cellular component | 73 | 1071 | 970 | 18698 | 1.3138833540289 | 0.0100291026058747 | 0.10768748923058 | 1.99873792547935 | 0.0752577319587629 | GJC1//CNIH1//RTN3//CDS1//SEC23A//CKAP4//OSBPL5//TBC1D20//PPM1L//VKORC1L1//RDH10//SPTSSA//DRD1//SMIM14//ACSL1//REEP3//RAB3GAP1//RAB21//SLC35D1//UBXN4//ARL6IP1//FLRT1//LCLAT1//GJA1//SERP1//RNF180//GRIA1//MIA3//SHISA2//DNAJB9//ATXN3//NR3C2//ASPH//DERL2//IER3IP1//SAR1B//ERGIC2//JKAMP//PIGA//PKD2//TMX3//UGT1A10//UGT1A8//UGT1A7//UGT1A6//UGT1A5//UGT1A9//UGT1A4//UGT1A1//UGT1A3//TMED9//KLHL14//STIM2//RHEB//MSMO1//XYLT2//WFS1//APOO//SCD5//ELOVL7//SGPP1//GSG1//MCFD2//CNEP1R1//NPLOC4//MAP3K5//ORMDL3//LBR//DNAJB12//RTN1//ELOVL4//EDEM1//MMGT1// |
| GO:0030285 | integral component of synaptic vesicle membrane | Cellular component | 4 | 18 | 970 | 18698 | 4.2836197021764 | 0.0122881623824793 | 0.130315203537651 | 1.91051305827051 | 0.00412371134020619 | STX6//SLC32A1//SLC17A7//SLC17A6// |
| GO:0016605 | PML body | Cellular component | 11 | 99 | 970 | 18698 | 2.1418098510882 | 0.0135220287226502 | 0.141651496009226 | 1.86895814589766 | 0.011340206185567 | HIPK3//TOPORS//WDFY3//MORC3//MKNK2//NFE2//PIAS4//PTEN//RNF4//UBE2I//SQSTM1// |
| GO:0048471 | perinuclear region of cytoplasm | Cellular component | 48 | 668 | 970 | 18698 | 1.3851225384283 | 0.0141209912940429 | 0.146143753272083 | 1.85013481472746 | 0.0494845360824742 | ADAM10//SYT6//STX6//CALCOCO2//CDKN1A//SEC23A//COPS8//SERINC3//CKAP4//STMN2//MTPN//PIKFYVE//EPHA5//FMR1//CD2AP//FLRT1//CLIC4//FBXL5//GJB2//NANOS1//INHBB//KIF5A//ARHGAP1//MYO1B//MSN//NF2//NME1//RASD1//NDFIP2//PPARG//TMEM100//PRKACB//PRKAR2B//TWF1//SLC2A4//STK33//SNAP25//TSC1//RNF128//CPEB4//FXR1//CALN1//RHPN2//PER2//TRIP10//BAG5//PPM1F//SLK// |
| GO:0031090 | organelle membrane | Cellular component | 178 | 2950 | 970 | 18698 | 1.16311165472654 | 0.0146475359414676 | 0.149788492544294 | 1.83423542766679 | 0.183505154639175 | PIKFYVE//RAB21//APPL1//GRB2//NCK1//SLC2A4//TBC1D20//NUP35//DUSP2//WDFY3//CNEP1R1//NSMF//GATA6//RANBP6//GNAQ//IPO5//LBR//DNAJB12//CCND2//ATXN3//TMEM135//BNIP3//ZNRF2//FLRT1//CLIC4//SNX5//PKD2//GPRC5C//CLIP1//YWHAG//FZD6//AP1S2//ULK2//MICU3//PRKAR2B//STAT3//SLC25A32//COX7A2L//BCL2L11//EPHA4//ACSL1//SLC44A1//GJA1//MTX3//NME1//PMAIP1//MIEF1//MOAP1//SNN//CNIH1//ADAM10//STX6//RTN3//SEC23A//FUT9//SERINC3//CLCN5//RAB12//HS3ST5//ST6GALNAC3//ARFIP1//RAB30//GOLGA3//GOLGA4//GRIA1//ST8SIA5//ST6GALNAC6//MIA3//RND3//MAN1A1//MAN2A1//NSF//ST8SIA3//NDFIP2//TMED9//PPP6C//SYBU//CHST7//PMEPA1//MARCH4//RHEB//XYLT2//ACBD3//ST3GAL3//PLEKHA3//TMF1//APOO//B3GNT5//GNPTG//ST6GAL2//CHST1//B3GALT2//ST3GAL5//MCFD2//DSEL//MMGT1//ITM2B//HS2ST1//EDEM1//HS3ST3A1//PLEKHF2//SBF2//IQGAP2//RAB9B//ORMDL3//CLCN6//VPS37A//STEAP2//ARHGAP1//LDLR//MYO1B//RAB23//RAB22A//KIF13A//SNX2//TSG101//ZFYVE21//LAMTOR3//WNT7A//WASL//ASPH//ERGIC2//DRD1//TMEM17//EPS15//GOLGA8A//TMEM87A//SAR1B//UXS1//CALN1//USP6NL//CALCOCO2//ATG16L1//MMD//MCOLN2//OSTM1//SPHK2//GPR137B//VLDLR//AHNAK//CYBRD1//ZFYVE9//LAPTM4A//SLC32A1//SYT6//SLC17A7//SLC17A6//C16ORF70//LHFPL2//TMX3//CKAP4//RAP1B//ITGAV//SNAP25//RAP2C//ENPP4//LRP6//LDLRAD4//CHMP3//PLEKHB2//RAB38//CHRM2//ACOX1//PEX5L//ACBD5//NAPEPLD//COX7A2//CTSA//RRAGD//NECAP1//DERL2//PSD//IER3IP1//CASD1//COX8C//IMMP2L//ARL6IP1//RDH10// |
| GO:0005884 | actin filament | Cellular component | 11 | 101 | 970 | 18698 | 2.09939777482903 | 0.0155411838795717 | 0.157057375912378 | 1.80851590105753 | 0.011340206185567 | IQGAP2//CD2AP//PKD2//ACTC1//PALLD//ARHGAP6//MYO1B//TWF1//TMOD1//TSC1//WASL// |
| GO:0043292 | contractile fiber | Cellular component | 20 | 230 | 970 | 18698 | 1.67619901389511 | 0.0167846211466964 | 0.16765104145363 | 1.77508845696325 | 0.0206185567010309 | TMOD3//TWF1//TMOD1//CALD1//NPNT//MMP2//ACTC1//CALM2//SQSTM1//AHNAK//FXR1//CFL2//FHL2//PALLD//PPP1R12A//SCN1A//RPL15//KAT2B//GJA1//DEK// |
| GO:0005795 | Golgi stack | Cellular component | 14 | 144 | 970 | 18698 | 1.87408361970218 | 0.0176151523979412 | 0.172702846383715 | 1.75411359515668 | 0.0144329896907217 | RAB30//GOLGA8A//MAN2A1//FUT9//RAB21//TMEM87A//GOLGA3//SAR1B//ST3GAL3//UXS1//ST6GAL2//SULF1//NSF//MARCH4// |
| GO:0030027 | lamellipodium | Cellular component | 17 | 187 | 970 | 18698 | 1.75238987816307 | 0.0176924918297636 | 0.172702846383715 | 1.75221099627601 | 0.0175257731958763 | EPHA2//ITGAV//IQGAP2//WASF3//STMN2//KLHL2//FGD4//FGD5//STX2//PALLD//NF2//PKD2//ENAH//SNX2//ACTC1//TSC1//WASL// |
| GO:0000307 | cyclin-dependent protein kinase holoenzyme complex | Cellular component | 6 | 41 | 970 | 18698 | 2.82092029167714 | 0.0182489269239884 | 0.174328007915079 | 1.73876266790296 | 0.00618556701030928 | CCNY//CCND2//CCNE2//CDK6//CDKN1A//CCNC// |
| GO:0043228 | non-membrane-bounded organelle | Cellular component | 241 | 4125 | 970 | 18698 | 1.12620256169947 | 0.0184678099188268 | 0.174328007915079 | 1.733584604103 | 0.248453608247423 | DYNC1LI2//FBXO28//MTBP//NUP43//PPP1R12A//CLIP1//KAT2B//PALLD//GJA1//DEK//SERP1//NCK1//RPL15//ZMYND11//MYT1L//FMR1//HMGB3//ZFR//ZBTB4//RAD21//RBBP8//CLOCK//CDK8//CDKN1A//AKAP11//KLF6//DDX6//E2F5//PPM1E//WDFY3//GOLGA3//GRB2//SERTAD3//MDFIC//MNX1//IPO5//YPEL2//DNAJB9//ATXN3//NF2//NOVA1//ACOX1//ERGIC2//CMPK1//PIK3CB//BRWD1//FBXW7//CDCA7L//TRERF1//RBL2//REV3L//ATXN1//ZMAT3//SLC14A1//SNAPC1//TSG101//ZNF655//FXR1//PCGF5//RUNX3//PER2//CCND2//COX7A2L//HAND1//CKAP4//RDH10//RAB3GAP1//RAP1B//TMEM135//TSC1//LMLN//TOPORS//CETN1//NEDD1//CEP128//DZIP1//MDM1//CEP41//MZT1//MAP7D1//PTP4A1//EVI5//KBTBD8//KIF23//CALCOCO2//CDC42EP3//WASF3//PTPN21//FGD4//SLAIN1//FRMD6//FGD5//DCX//KLHL3//MYLIP//TMOD3//HSBP1//JAK1//MSN//NEFH//PFN2//SNTG1//ENAH//MAPK1//PTPN4//SNAP25//TGFB1I1//TMOD1//RNF128//CALD1//CCDC6//SGCE//TRIP10//DCTN6//CENPO//TWF1//NPNT//ZBTB18//JUN//HMGA2//ESCO2//PKD2//HECW2//CDC14A//CORO2B//CFL2//IQGAP2//ZNF268//KLHL2//CNN3//CRK//CD2AP//CLIC4//ARHGAP6//MARCKS//LIMA1//CENPQ//TMEM63B//RAB22A//KLHL14//AHNAK//WASL//STK17B//TACC2//PPP2CA//SLAIN2//MID1IP1//NSMF//MPP1//DLC1//NEFM//ZNF131//SESTD1//DCP2//PAN3//CNOT7//YTHDF2//CPEB1//SQSTM1//MBNL1//TDRD5//USP3//CEBPB//RNF2//FRY//LATS2//CALM2//HAUS8//TBL1XR1//MYO1B//ACTC1//MTPN//DYNLL2//LCA5//FLII//CC2D1A//PXK//C7ORF43//CEP97//FAM110B//DPYSL2//REEP3//WDR47//RASSF3//KIF5A//SYBU//KIF13A//CCT6A//RANBP9//LRP8//MMP2//KRT27//NME1//NABP1//KLF2//POLR3G//E2F1//ESR1//H2AFZ//IRF1//AR//MEF2D//POU4F1//SMARCD2//STAT3//KLF4//HAND2//SYCP3//UBE2I//ANP32E//FOXC1//APPL1//RBBP7//BRMS1L//FHL2//SCN1A//PTGES3//DPY30//BAHD1//MAF//GLYR1//SESN1//NFIB//PAK6//BTBD10//BCL2L11//ATG16L1//CDK6//VPS37A//PPP4R2//RAB23//PRKACB//PRKAR2B//RRAGD//CAPRIN2//TFAP2A//MASTL//CAMK2N2//RAB28//MAPRE3//ZNF804A// |
| GO:0043232 | intracellular non-membrane-bounded organelle | Cellular component | 241 | 4125 | 970 | 18698 | 1.12620256169947 | 0.0184678099188268 | 0.174328007915079 | 1.733584604103 | 0.248453608247423 | DYNC1LI2//FBXO28//MTBP//NUP43//PPP1R12A//CLIP1//KAT2B//PALLD//GJA1//DEK//SERP1//NCK1//RPL15//ZMYND11//MYT1L//FMR1//HMGB3//ZFR//ZBTB4//RAD21//RBBP8//CLOCK//CDK8//CDKN1A//AKAP11//KLF6//DDX6//E2F5//PPM1E//WDFY3//GOLGA3//GRB2//SERTAD3//MDFIC//MNX1//IPO5//YPEL2//DNAJB9//ATXN3//NF2//NOVA1//ACOX1//ERGIC2//CMPK1//PIK3CB//BRWD1//FBXW7//CDCA7L//TRERF1//RBL2//REV3L//ATXN1//ZMAT3//SLC14A1//SNAPC1//TSG101//ZNF655//FXR1//PCGF5//RUNX3//PER2//CCND2//COX7A2L//HAND1//CKAP4//RDH10//RAB3GAP1//RAP1B//TMEM135//TSC1//LMLN//TOPORS//CETN1//NEDD1//CEP128//DZIP1//MDM1//CEP41//MZT1//MAP7D1//PTP4A1//EVI5//KBTBD8//KIF23//CALCOCO2//CDC42EP3//WASF3//PTPN21//FGD4//SLAIN1//FRMD6//FGD5//DCX//KLHL3//MYLIP//TMOD3//HSBP1//JAK1//MSN//NEFH//PFN2//SNTG1//ENAH//MAPK1//PTPN4//SNAP25//TGFB1I1//TMOD1//RNF128//CALD1//CCDC6//SGCE//TRIP10//DCTN6//CENPO//TWF1//NPNT//ZBTB18//JUN//HMGA2//PKD2//HECW2//CDC14A//CORO2B//CFL2//IQGAP2//ZNF268//KLHL2//CNN3//CRK//CD2AP//CLIC4//ARHGAP6//MARCKS//LIMA1//CENPQ//TMEM63B//RAB22A//KLHL14//AHNAK//WASL//STK17B//TACC2//PPP2CA//SLAIN2//MID1IP1//NSMF//MPP1//NEFM//ZNF131//SESTD1//DCP2//YTHDF2//USP3//ESCO2//DLC1//PAN3//CNOT7//CPEB1//SQSTM1//MBNL1//TDRD5//CEBPB//RNF2//FRY//LATS2//CALM2//HAUS8//TBL1XR1//MYO1B//ACTC1//MTPN//DYNLL2//LCA5//FLII//CC2D1A//PXK//C7ORF43//CEP97//FAM110B//DPYSL2//REEP3//WDR47//RASSF3//KIF5A//SYBU//KIF13A//CCT6A//RANBP9//LRP8//MMP2//KRT27//NME1//NABP1//KLF2//POLR3G//E2F1//ESR1//H2AFZ//IRF1//AR//MEF2D//POU4F1//SMARCD2//STAT3//KLF4//HAND2//SYCP3//UBE2I//ANP32E//FOXC1//APPL1//RBBP7//BRMS1L//FHL2//SCN1A//PTGES3//DPY30//BAHD1//MAF//GLYR1//SESN1//NFIB//PAK6//BTBD10//BCL2L11//ATG16L1//CDK6//VPS37A//PPP4R2//RAB23//PRKACB//PRKAR2B//RRAGD//CAPRIN2//TFAP2A//MASTL//CAMK2N2//RAB28//MAPRE3//ZNF804A// |
| GO:0045178 | basal part of cell | Cellular component | 7 | 53 | 970 | 18698 | 2.54592491733126 | 0.0190227159141995 | 0.177614271416276 | 1.72072747781155 | 0.00721649484536083 | EPS15//ITGA6//MET//PKD2//BMPR2//EDN1//CLDN11// |
| GO:0015629 | actin cytoskeleton | Cellular component | 36 | 487 | 970 | 18698 | 1.42494125616546 | 0.0209979517783866 | 0.19394882341542 | 1.67782306596737 | 0.0371134020618557 | PALLD//TMOD3//TMOD1//DCTN6//ARHGAP6//MYO1B//TWF1//ACTC1//TSC1//WASL//MTPN//DYNLL2//DLC1//NF2//MARCKS//KAT2B//LIMA1//IQGAP2//CD2AP//PKD2//CALD1//CORO2B//CDC42EP3//CFL2//ZNF268//KLHL2//CNN3//CRK//CLIC4//PPP1R12A//CENPQ//TMEM63B//RAB22A//KLHL14//AHNAK//STK17B// |
| GO:0016363 | nuclear matrix | Cellular component | 11 | 106 | 970 | 18698 | 2.00036957790313 | 0.0215720929184278 | 0.197132210818399 | 1.66610771772406 | 0.011340206185567 | CEBPB//CFL2//MORC3//CLIC4//NSMF//ATXN3//MYB//PIAS4//RAD21//ATXN1//TGFB1I1// |
| GO:0000791 | euchromatin | Cellular component | 5 | 32 | 970 | 18698 | 3.01192010309278 | 0.0233454867974574 | 0.208893470406416 | 1.63179706577921 | 0.00515463917525773 | NSMF//H2AFZ//JUN//KLF4//RNF2// |
| GO:0008305 | integrin complex | Cellular component | 5 | 32 | 970 | 18698 | 3.01192010309278 | 0.0233454867974574 | 0.208893470406416 | 1.63179706577921 | 0.00515463917525773 | ITGA6//NPNT//ITGAV//ITGB8//ITGA5// |
| GO:0044456 | synapse part | Cellular component | 51 | 740 | 970 | 18698 | 1.32850097520201 | 0.0236282575837972 | 0.209244054273008 | 1.62656830332078 | 0.0525773195876289 | STX2//GRIA1//NTF3//SNAP25//SLC32A1//SYT6//SLC17A7//SLC17A6//C16ORF70//FMR1//PPFIA1//KCNJ10//WNT7A//ZNF804A//RAB3GAP1//PSD//ZNRF2//NPTN//FOSL1//CHRM2//CHRM5//EPHA4//EPHA7//CPEB3//NSMF//GLRA1//GLRB//P2RY1//MINK1//PTEN//CPEB1//STRN//CPEB4//FCHO2//STX6//PFN2//CNN3//LPAR1//GPM6A//KCNA4//PRKAR2B//FXR1//ADAM10//EPS8//NEFH//NSF//BMPR2//BNIP3//SOS1//SPOCK1//LRP8// |
| GO:0005868 | cytoplasmic dynein complex | Cellular component | 4 | 22 | 970 | 18698 | 3.50477975632615 | 0.0249354859605914 | 0.218567167756612 | 1.60318216352155 | 0.00412371134020619 | BCL2L11//TOPORS//DYNLL2//DYNC1LI2// |
| GO:0098590 | plasma membrane region | Cellular component | 70 | 1069 | 970 | 18698 | 1.26224528174515 | 0.0258957573235671 | 0.224691470110547 | 1.58677138348228 | 0.0721649484536082 | EPS15//ITGA6//MET//PKD2//BMPR2//STX2//SLC41A1//DSTYK//KCNJ10//LDLR//MSN//P2RY1//SLC14A1//SLC4A4//ADAM9//GJA1//DLL1//PRKAA1//PTEN//FZD6//RAB21//RHOC//NF2//LIMA1//PSD//MASTL//EPHA2//ITGAV//SLC6A14//CYBRD1//DLC1//FGD5//EPS8//ITGA5//NME1//ARHGEF4//PLEK//SNTG1//TWF1//PLEKHA1//DRD1//TMEM17//SPRED1//EFNA5//LRP6//MAPK1//TGFBR2//LRP8//GRIA1//ZNRF2//FMR1//NPTN//SNAP25//FOSL1//CHRM2//CHRM5//EPHA4//EPHA7//CPEB3//NSMF//GLRA1//GLRB//MINK1//CPEB1//STRN//CPEB4//FCHO2//NRG1//SGCE//NAPEPLD// |
| GO:0098858 | actin-based cell projection | Cellular component | 17 | 197 | 970 | 18698 | 1.66343607724109 | 0.0277349834266916 | 0.238243507635281 | 1.55697208930195 | 0.0175257731958763 | IQGAP2//CLIC4//MSN//FGD4//EPHA4//GPM6A//ITGA6//MYO1B//ENAH//TWF1//ACTC1//EPS8//RHOC//MPP1//FMR1//ITGAV//NF2// |
| GO:0043197 | dendritic spine | Cellular component | 14 | 153 | 970 | 18698 | 1.76384340677852 | 0.0280639950352001 | 0.238682888467692 | 1.55185050514506 | 0.0144329896907217 | GRIA1//FMR1//CNN3//LPAR1//EPHA4//GPM6A//KCNA4//PRKAR2B//PSD//PTEN//STRN//CPEB4//FXR1//ZNF804A// |
| GO:0001891 | phagocytic cup | Cellular component | 4 | 23 | 970 | 18698 | 3.35239802779023 | 0.0289798736952544 | 0.244055995139446 | 1.53790351167277 | 0.00412371134020619 | SNX5//TNF//TRIP10//ARHGAP12// |
| GO:0000407 | phagophore assembly site | Cellular component | 5 | 34 | 970 | 18698 | 2.83474833232262 | 0.0296450132944528 | 0.247233654562475 | 1.52804835055747 | 0.00515463917525773 | ATG16L1//ULK2//TRAPPC8//PLEKHF2//SQSTM1// |
| GO:0031985 | Golgi cisterna | Cellular component | 11 | 112 | 970 | 18698 | 1.89320692194404 | 0.0308883699833473 | 0.252804286576832 | 1.51020500933908 | 0.011340206185567 | GOLGA8A//MAN2A1//FUT9//RAB21//TMEM87A//GOLGA3//SAR1B//ST3GAL3//UXS1//ST6GAL2//RAB30// |
| GO:0044309 | neuron spine | Cellular component | 14 | 155 | 970 | 18698 | 1.74108413701363 | 0.0309015717003113 | 0.252804286576832 | 1.51001943114431 | 0.0144329896907217 | CNN3//LPAR1//EPHA4//FMR1//GPM6A//GRIA1//KCNA4//PRKAR2B//PSD//PTEN//STRN//CPEB4//FXR1//ZNF804A// |
| GO:1902911 | protein kinase complex | Cellular component | 10 | 99 | 970 | 18698 | 1.94709986462564 | 0.0325690166227643 | 0.26074816437943 | 1.48719535416938 | 0.0103092783505155 | PRKAA1//CDK6//CDKN1A//CCNC//CCND2//ACVR1//TGFBR2//CCNY//CCNE2//MAP3K5// |
| GO:0015630 | microtubule cytoskeleton | Cellular component | 74 | 1152 | 970 | 18698 | 1.23823382016037 | 0.0326645422287458 | 0.26074816437943 | 1.48592342377213 | 0.0762886597938144 | FRY//TACC2//AKAP11//LCA5//DZIP1//FLII//LATS2//YTHDF2//CC2D1A//PXK//C7ORF43//MAPK1//TSG101//EVI5//CEP97//FAM110B//MZT1//MAP7D1//PTP4A1//KBTBD8//KIF23//DYNLL2//DCX//DYNC1LI2//DPYSL2//REEP3//WDR47//RASSF3//KIF5A//SYBU//MDM1//MID1IP1//CLIP1//KIF13A//CCT6A//HAUS8//RANBP9//LRP8//CDK6//DCTN6//CETN1//NEDD1//VPS37A//PPP4R2//E2F1//CLIC4//MARCKS//PPP1R12A//NME1//RAB23//PRKACB//PRKAR2B//SLAIN2//RRAGD//CAPRIN2//TFAP2A//CALM2//PCGF5//MASTL//CDC14A//KAT2B//CAMK2N2//CEP41//TOPORS//PKD2//RAB28//HECW2//TBL1XR1//SERP1//ZNF804A//BCL2L11//CEP128//PPP2CA//MAPRE3// |
| GO:0009925 | basal plasma membrane | Cellular component | 5 | 35 | 970 | 18698 | 2.75375552282769 | 0.0331589639349577 | 0.26074816437943 | 1.47939904761393 | 0.00515463917525773 | EPS15//ITGA6//MET//PKD2//BMPR2// |
| GO:0098636 | protein complex involved in cell adhesion | Cellular component | 5 | 35 | 970 | 18698 | 2.75375552282769 | 0.0331589639349577 | 0.26074816437943 | 1.47939904761393 | 0.00515463917525773 | ITGA5//ITGAV//ITGB8//ITGA6//NPNT// |
| GO:0005719 | nuclear euchromatin | Cellular component | 4 | 24 | 970 | 18698 | 3.2127147766323 | 0.0333903353687279 | 0.26074816437943 | 1.47637921891938 | 0.00412371134020619 | NSMF//H2AFZ//JUN//KLF4// |
| GO:0005802 | trans-Golgi network | Cellular component | 17 | 202 | 970 | 18698 | 1.62226191691334 | 0.0341225639184115 | 0.2640656072605 | 1.46695834404074 | 0.0175257731958763 | STX6//ARFIP1//MYO1B//KIF13A//CALN1//AP1S2//USP6NL//FAM91A1//RAB12//RAB21//RAB38//RAB30//GOLGA4//MARCH4//SNAP25//C16ORF70//DPY30// |
| GO:0031410 | cytoplasmic vesicle | Cellular component | 137 | 2279 | 970 | 18698 | 1.15877645738997 | 0.0346463769328873 | 0.265725337369198 | 1.46034217404852 | 0.141237113402062 | STMN2//CLCN5//CRKL//DGKH//S1PR1//LPAR1//RAB12//RAB21//STEAP2//SNX5//GRB2//JAK1//KDR//RUFY2//ANKRD50//ARRDC3//CLIP1//SNX2//TSG101//ARRDC4//ADAM10//EDN1//TMED9//PLEKHF2//CD2AP//RAB9B//STX6//TRIM3//FGD5//ADRB1//RAB38//APPL1//GJA1//LDLR//LRP6//MYO1B//NF2//DERL2//MAPK1//RAB22A//PTP4A1//MMGT1//ZFYVE9//DYNC1LI2//CHMP3//FYCO1//RNF128//SQSTM1//TBC1D12//GRIA1//TNF//TBC1D17//ARHGAP1//IER3IP1//SLC2A4//IGF1//ITGAV//RAB23//FCHO2//DENND1B//STX2//NTF3//SNAP25//CKAP4//PIKFYVE//EPHA4//EPS15//PMEPA1//LDLRAD4//VPS37A//MMD//MCOLN2//LAPTM4A//NDFIP2//ITM2B//CNIH1//SEC23A//SAR1B//MCFD2//IQGAP2//ORMDL3//IDH1//IMPDH1//SERPINI1//WNT7A//WASL//SLC32A1//SYT6//SLC17A7//SLC17A6//C16ORF70//CHRM2//LHFPL2//TMX3//NHLRC2//TGFB2//VEGFB//LAMTOR3//RAP1B//GNS//C6ORF120//CTSA//TTR//CDC37L1//PLEKHB2//RAP2C//ENPP4//CLCN6//ZNRF2//KIF13A//ZFYVE21//NECAP1//FLRT1//CLIC4//PKD2//GPRC5C//YWHAG//FZD6//AP1S2//ULK2//RRAGD//PSD//CALCOCO2//SPRED1//DSC2//TRAPPC8//RGS17//DLL1//NRBF2//AGFG1//ITGA5//RHOC//RND3//CHIC1//SYBU//ANP32E//USP6NL// |
| GO:0097708 | intracellular vesicle | Cellular component | 137 | 2282 | 970 | 18698 | 1.15725308781409 | 0.0358951387789952 | 0.272866585939441 | 1.44496436324367 | 0.141237113402062 | CALCOCO2//SPRED1//DSC2//TRAPPC8//FLRT1//RGS17//DLL1//NRBF2//AGFG1//ITGA5//RHOC//RND3//LRP6//NTF3//CHMP3//CHIC1//SYBU//ANP32E//WASL//USP6NL//STMN2//CLCN5//CRKL//DGKH//S1PR1//LPAR1//RAB12//RAB21//STEAP2//SNX5//GRB2//JAK1//KDR//RUFY2//ANKRD50//ARRDC3//CLIP1//SNX2//TSG101//ARRDC4//ADAM10//EDN1//TMED9//PLEKHF2//CD2AP//RAB9B//STX6//TRIM3//FGD5//ADRB1//RAB38//APPL1//GJA1//LDLR//MYO1B//NF2//DERL2//MAPK1//RAB22A//PTP4A1//MMGT1//ZFYVE9//DYNC1LI2//FYCO1//RNF128//SQSTM1//SLC2A4//TBC1D12//GRIA1//TNF//TBC1D17//ARHGAP1//IER3IP1//STX2//SNAP25//IGF1//ITGAV//RAB23//FCHO2//DENND1B//CKAP4//PIKFYVE//EPHA4//EPS15//PMEPA1//LDLRAD4//VPS37A//MMD//MCOLN2//LAPTM4A//NDFIP2//ITM2B//CNIH1//SEC23A//SAR1B//MCFD2//IQGAP2//ORMDL3//IDH1//IMPDH1//SERPINI1//WNT7A//SLC32A1//SYT6//SLC17A7//SLC17A6//C16ORF70//CHRM2//LHFPL2//TMX3//NHLRC2//TGFB2//VEGFB//LAMTOR3//RAP1B//GNS//C6ORF120//CTSA//TTR//CDC37L1//PLEKHB2//RAP2C//ENPP4//CLCN6//ZNRF2//KIF13A//ZFYVE21//RRAGD//PSD//NECAP1//CLIC4//PKD2//GPRC5C//YWHAG//FZD6//AP1S2//ULK2// |
| GO:0060077 | inhibitory synapse | Cellular component | 3 | 15 | 970 | 18698 | 3.85525773195876 | 0.039684485703442 | 0.299026080870673 | 1.40137924362103 | 0.00309278350515464 | SLC32A1//NPTN//GLRA1// |
| GO:0031253 | cell projection membrane | Cellular component | 25 | 332 | 970 | 18698 | 1.45152776052664 | 0.0401311053034022 | 0.299761908309761 | 1.39651887862754 | 0.0257731958762887 | EPHA2//ITGAV//SLC6A14//CYBRD1//NF2//MSN//DLC1//FGD5//EPS8//ITGA5//NME1//ARHGEF4//PLEK//SNTG1//PSD//TWF1//PLEKHA1//DRD1//TMEM17//EPS15//PKD2//NRG1//SGCE//GRIA1//NAPEPLD// |
| GO:0031312 | extrinsic component of organelle membrane | Cellular component | 5 | 37 | 970 | 18698 | 2.60490387294511 | 0.040934831638016 | 0.303129486009101 | 1.38790699149235 | 0.00515463917525773 | SNX5//GOLGA3//PLEKHF2//WDFY3//PSD// |
| GO:0031519 | PcG protein complex | Cellular component | 6 | 50 | 970 | 18698 | 2.31315463917526 | 0.0437023271572997 | 0.320857256650602 | 1.35949543614939 | 0.00618556701030928 | EZH1//JARID2//RBBP7//RNF2//SKP1//PCGF5// |
| GO:0005770 | late endosome | Cellular component | 19 | 240 | 970 | 18698 | 1.52603951890034 | 0.0445935754559204 | 0.324626112852844 | 1.35072770507753 | 0.0195876288659794 | GJA1//SLC2A4//TSG101//VPS37A//PIKFYVE//MMD//MCOLN2//CHMP3//LAPTM4A//NDFIP2//LAMTOR3//DYNC1LI2//LDLR//DERL2//MAPK1//RAB22A//FYCO1//RNF128//SQSTM1// |
| GO:0000775 | chromosome, centromeric region | Cellular component | 16 | 194 | 970 | 18698 | 1.58979700286959 | 0.0454814753110727 | 0.328307456237071 | 1.34216545627397 | 0.0164948453608247 | DYNC1LI2//FBXO28//MTBP//NUP43//PPP1R12A//CLIP1//KAT2B//CEBPB//DCTN6//CENPO//ESCO2//FMR1//SYCP3//PPP2CA//CENPQ//RAD21// |
| GO:0032580 | Golgi cisterna membrane | Cellular component | 9 | 91 | 970 | 18698 | 1.9064461311884 | 0.046252103708899 | 0.331087975716202 | 1.33486850923006 | 0.00927835051546392 | FUT9//RAB21//GOLGA8A//TMEM87A//GOLGA3//SAR1B//ST3GAL3//UXS1//ST6GAL2// |
| GO:0032155 | cell division site part | Cellular component | 7 | 64 | 970 | 18698 | 2.10834407216495 | 0.0472432196826543 | 0.335387815763637 | 1.32566051235485 | 0.00721649484536083 | MZT1//RAB21//RHOC//NF2//LIMA1//PSD//MASTL// |
| GO:0005901 | caveola | Cellular component | 8 | 78 | 970 | 18698 | 1.97705524715834 | 0.0487685872224351 | 0.34109895457623 | 1.31185982530627 | 0.00824742268041237 | DLC1//SPRED1//EFNA5//LRP6//MAPK1//BMPR2//TGFBR2//LRP8// |
| GO:0098563 | intrinsic component of synaptic vesicle membrane | Cellular component | 4 | 27 | 970 | 18698 | 2.8557464681176 | 0.0488418759172017 | 0.34109895457623 | 1.3112076640383 | 0.00412371134020619 | STX6//SLC32A1//SLC17A7//SLC17A6// |
